# Supplementary material for: Continuous-Flow Synthesis of Arylthio-Cyclopropyl Carbonyl Compounds
Source: Molecules. 2022 Nov 16;27(22):7943. doi: 10.3390/molecules27227943 (PMC9699303; doi:10.3390/molecules27227943)
Supplement: Supplementary file 1 [file molecules-27-07943-s001.zip › molecules-1982802-supplementary.pdf]

molecules

Supporting Materials

Continuous-Flow Synthesis of Arylthio-Cyclopropyl Carbonyl Compounds

Davide Moi [1], Maria Chiara Cabua [1], Viktoria Velichko [1], Andrea Cocco [1], Annalisa Chiappone [1], Rita Mocci [1], Stefania Porcu [2], Monica Piras [3], Stefano Bianco [4], Fabio Pesciaioli [5,*] and Francesco Secci [1,*]

Table of contents

1. General remarks S2
   1.1. Catalyst milling procedure and column filling S2
   1.2. Catalyst loading and continuous flow parameters S3
   1.3. Continuous Flow system scheme S3
2. Grinded amberlyst 35 thermogravimetric analysis S4
3. ATR-FTIR analysis of grinded AR-35 S5
4. Brunauer–Emmett–Teller (BET) surface are analysis of AR-35 S6
5. Scanning electron microscopy analysis of grinded AR-35 S8
6. AR-35 columns performance testing S10
7. ^{1}H and ^{13}C NMR characterization of compounds 3-7 S12
8. References and notes S25
9. Full BET original analysis reports

1. General remarks

Unless stated otherwise, respectively the synthesis of compounds **3a-j** were performed at room temperature using a continuous flow system constituted by an AR-35 packed column, two syringe pumps KD scientific Legato Syringe (or two HPLC pumps, Hitachi Elite la Chrom D2130) and a collecting flask. Synthesis of compounds **4**-**8** were performed at the indicated temperatures in a round bottom flask equipped with a stirring bar. Commercially available reagents were used as received unless otherwise noted. The resins used in this work were purchased from Sigma Aldrich (Amberlyst 15 and Amberlyst 35) and used after ball mill grinding. Al the organic solvents used in these reactions have to be considered HPLC grade. ^{1}H NMR spectra were recorded on 400 and 500 MHz Varian spectrometers at 300.15 K using $CDCl_3$ (ref. 7.27 ppm), as a solvent. ^{13}C NMR were recorded at 101 MHz and 126 MHz (ref. $CDCl_3$ 77.00 ppm) at 300.15 K using $CDCl_3$, as solvent. Chemical shifts (d) are given in ppm. Coupling constant values (*J*) are reported in Hz. Infrared spectra were recorded on a FT-IR Bruker Equinox-55 spectrophotometer and are reported in wavenumbers (cm^{-1}). Low Mass Spectra Analysis were recorded on an Agilent-HP GC-MS (E.I. 70eV). High Resolution Mass Spectra (HRMS) were obtained using a Bruker High Resolution Mass Spectrometer in fast atom bombardment (FAB^+) ionization mode. Melting points were determined with a Büchi M-560. Analytical thin layer chromatography was performed using 0.25 mm Aldrich silica gel 60-F plates. Flash chromatography was performed using Merk 70-200 mesh silica gel. Yields refer to chromatography and spectroscopically pure materials or using a Biotage Selekt Flash Chromatography instrument.

1.1. Catalyst milling procedure and column filling

Amberlyst 15 and amberlyst 35 (1.0g) was loaded into a zirconia SmartSnap™ grinding jar (15 mL) equipped with 1 ball (Ø = 8 mm). The jar was sealed and shaken for 15 minutes at a frequency of 20 Hz using a FormTech FTS-1000 Shaker Mill® apparatus.

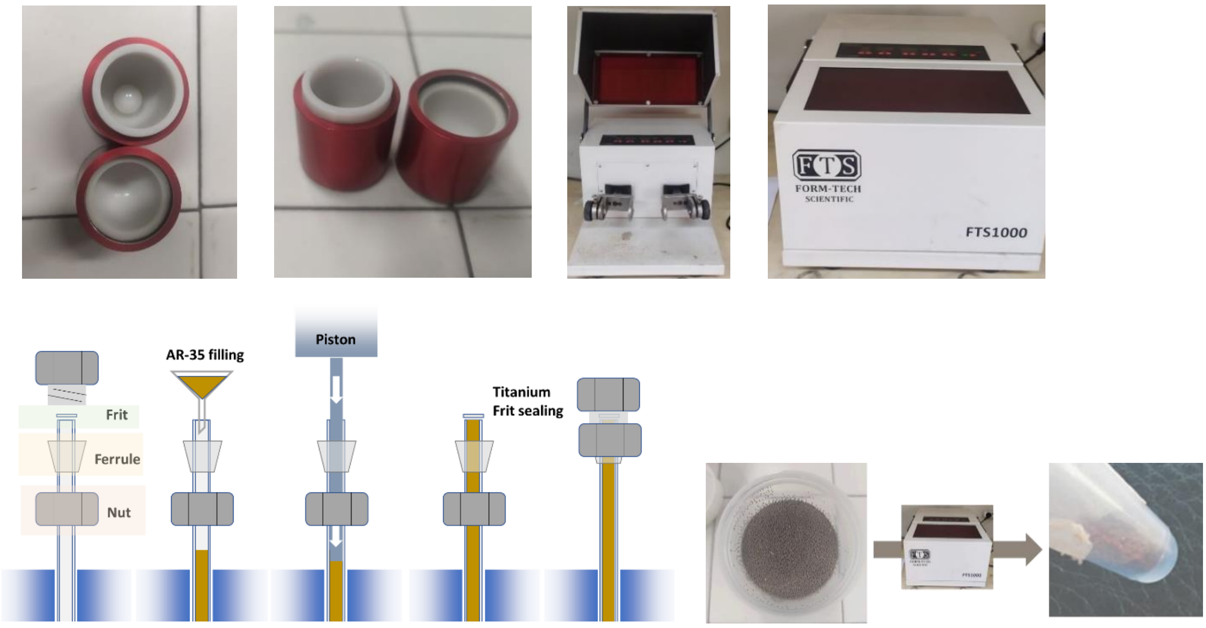

Figure S1. Process of grinding, filling and closure of the columns used for the synthesis of arylthio cyclopropyl carbaldehydes

After grinding, the powder was dried under vacuum at 50°C and filled portionwise (500 mg) in the column by using a funnel. The resin introduced into the column is pressed through an oil piston reaching an exerted pressure of 500 kg/cm^2. The system is then sealed with a porous titanium frit and subsequently closed by tightening the column. To keep the columns away from moisture before they are used for the first time, they are closed with HPLC column caps and stored in a desiccator.

1.2. Catalyst loading and continuous flow parameters

- 5 cm columns (internal diam. 0.8 cm). Total amount or loaded AR-35 4.0 g
- 10 cm columns (internal diam. 0.8 cm). Total amount of loaded AR-35 8.0g
- 15 cm columns (internal diam. 0.8 cm). Total amount of loaded AR-35, ca. 12g

In this work the void volume V0 is defined as the volume of the liquid phase within the column and can be converted from the residence time (τ_{res}) and the mobile phase flow rate (Fr).[1]

$V0$ [cm^3] = τ_{res} [min] x Flow rate [mL/min]

$V0_{15\ \mathrm{cm\ column}}$ [cm^3] = 5.6 min Fr [0.5 mL/min] = 2.8

1.3. Continuous Flow system scheme

For 15 cm columns packed with 12 g of AR-35.
The description of single parts (HPLC pumps, Raman probe etc. are reported in the general methods).

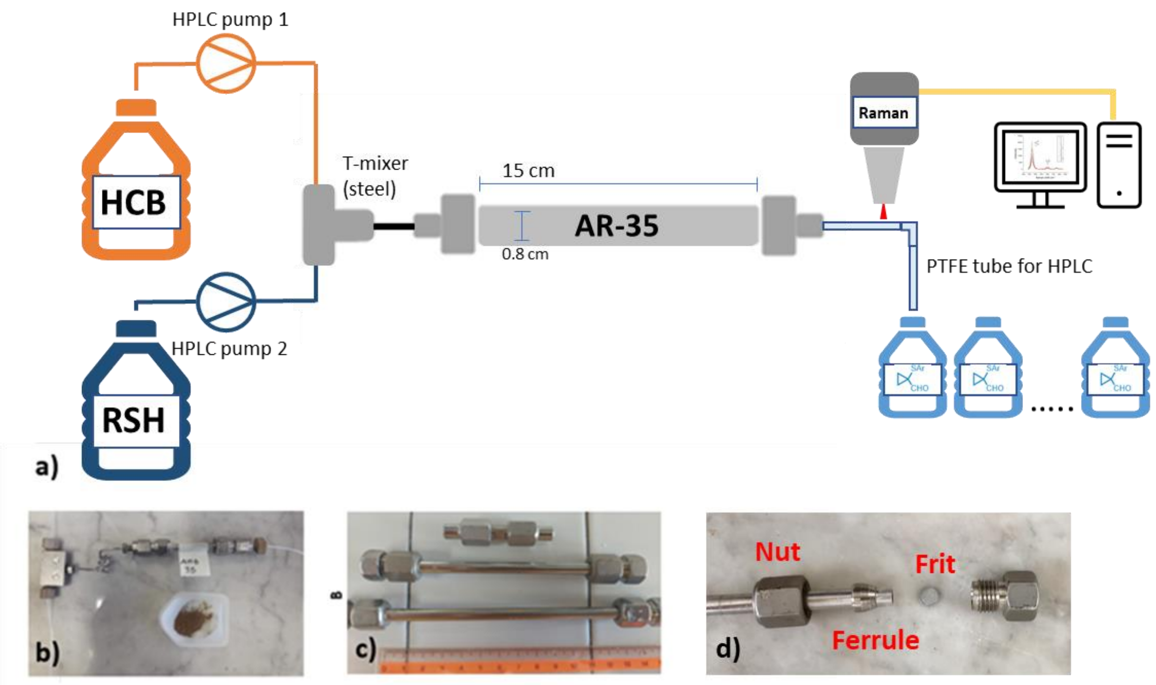

Figure S2. a) Representative diagram of the continuous flow system; b) 5 cm stainless steel column packed with AR-35; c) 2, 10 and 15 cm AR-35 packed columns; d) column components.

2. Grinded amberlyst 35 thermogravimetric analysis

TGA was performed with a NETZSCH TG 209F1 Libra instrument. The samples were dried overnight at 60°C. The test was performed under nitrogen flux imposing a heat ramp at 10°C/min up to 100, followed by 15 min of isothermal treatment (100°C) and then a second ramp up to 700 °C[2] (10°C/min) (figure S3)

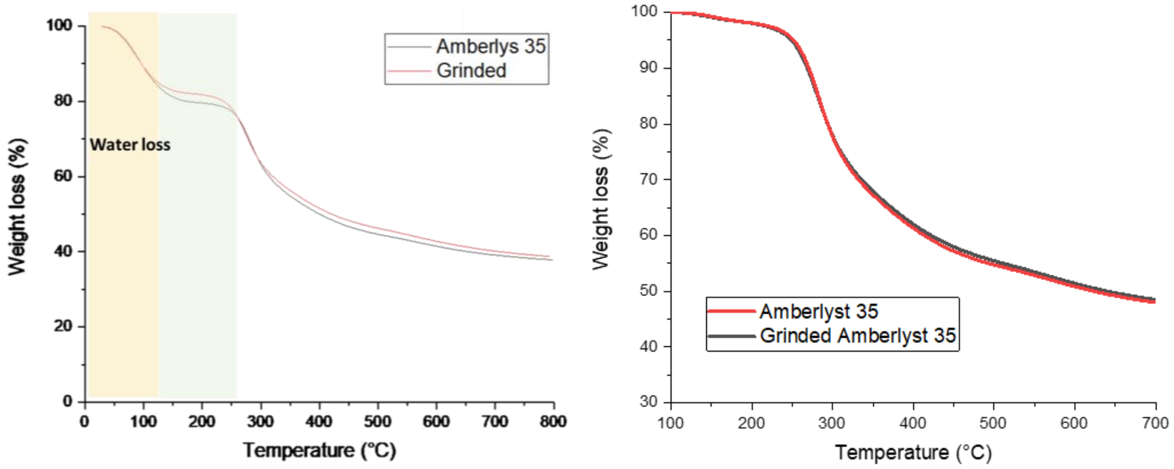

Figure S3. Comparison of TGA experiments between neat AR-35 and grinded AR-35 pre and post water removal by eating the sample.

From these analyses, it seems that the grinding process does not influence the thermal stability of the material. The grinded material presents a slightly higher weight loss during the first heating ramp up to 100°C that is linked to a higher water absorption before the measurement (figure S3).

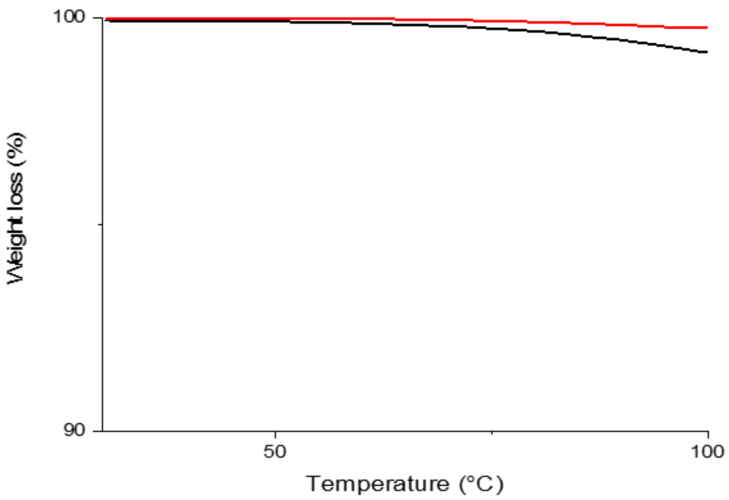

Figure S4. Comparison of TGA experiments between neat AR-35 and grinded AR-35

3. ATR-FTIR analysis of grinded AR-35

Attenuated total reflectance-infrared spectroscopy (ATR-FTIR) was performed with a Thermo Scientific Nicolet iS50 FTIR Spectrometer equipped with a diamond crystal ATR accessory. Thirty-two ATR spectra were collected with a resolution of 4 cm^{-1} in the range of 4000–500 cm^{-1}. The peak at 2923 cm^{-1} was due to the aliphatic C single bond H stretching absorbance of methyl group in the main chain and in aromatic rings[3] (figure S4).

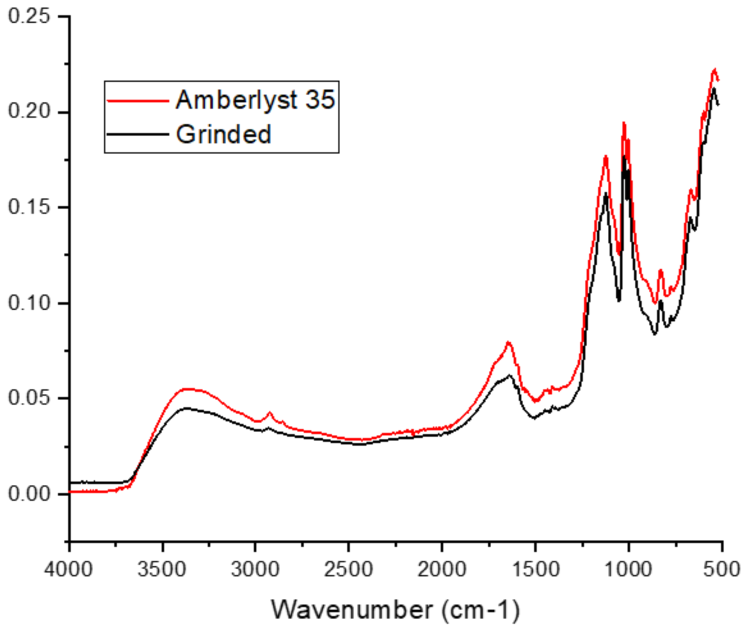

Figure S5. ATR-FTIR collected spectra of neat AR-35 and grinded AR-35

4. Brunauer–Emmett–Teller (BET) surface are analysis of AR-35

BET measurements were performed with a ASAP 2020 Plus Micrometrics instrument. An adsorptive analysis of N_2 was performed at 77 K (see attached document).

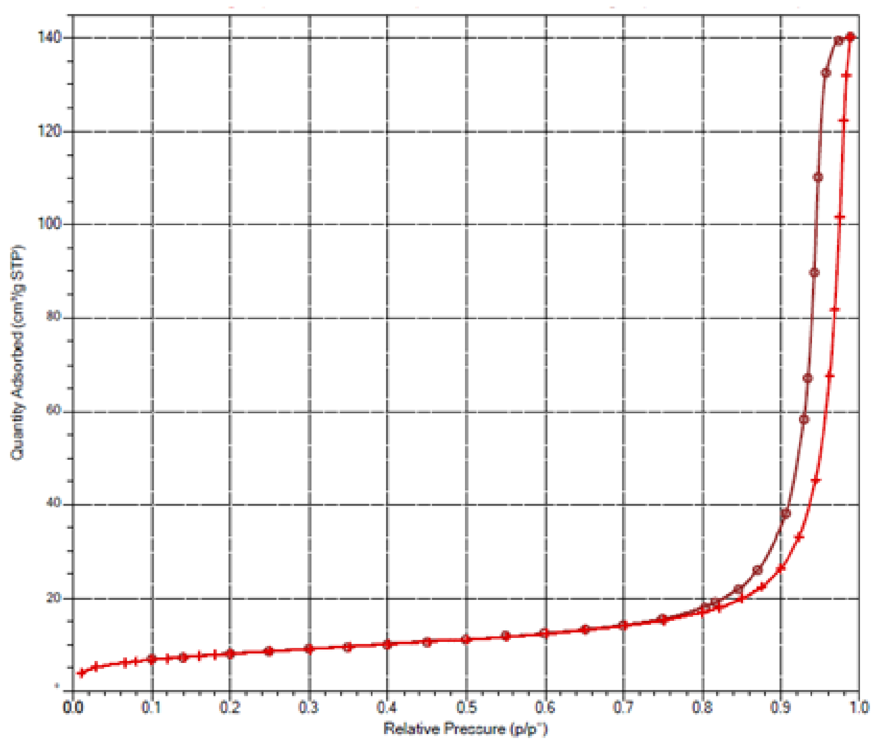

Figure S6. BET Isotherm Linear Plot Neat Amberlyst 35. Neat Amberlyst 35 BET surface area 28,6015 m²/g, grinded sample BET surface area 4,8426 m²/g

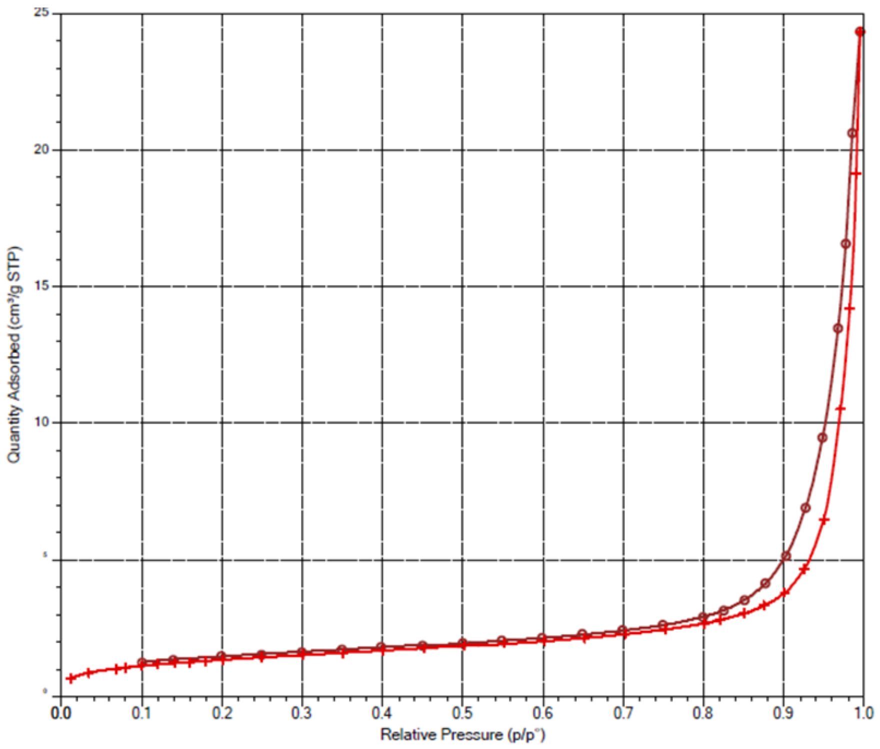

Figure S7. BET Isotherm Linear Plot grinded Amberlyst 35

EDX spectroscopy analysis was performed using a Bruker nano analytics probe for the characterization of AR-35, coupled to STEM.

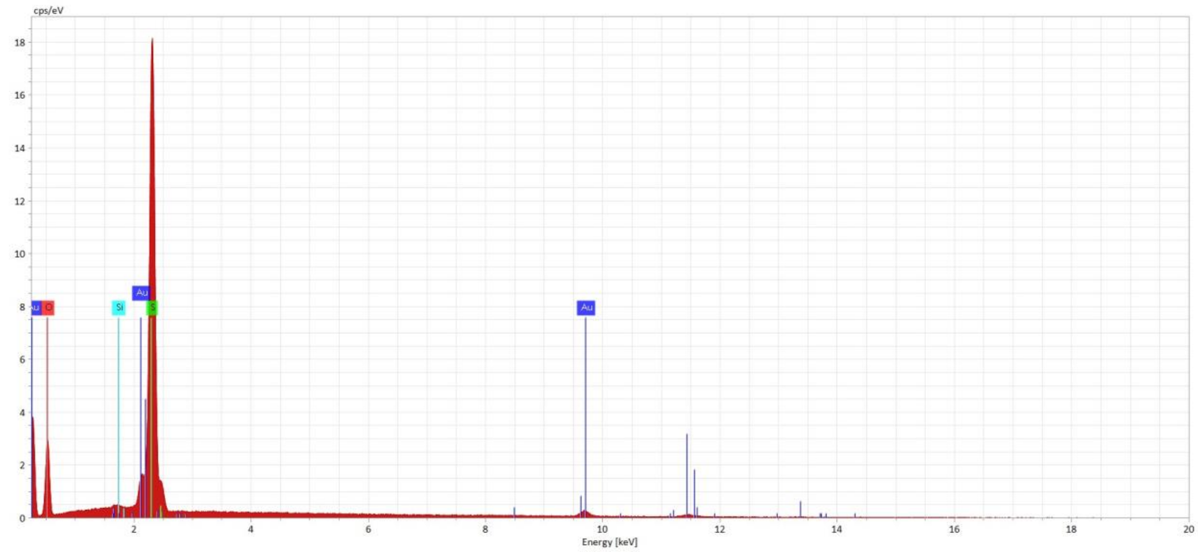

Figure S8. EDX (energy dispersive x-ray spectroscopy) analysis of a grinded AR-35 sample. Analysis of the surface material during the STEM analysis process and elements abundance. The higher peak is related to the amount of sulphur (as sulfonic acid) which is present in the amberlyst 35.

5. Scanning electron microscopy analysis of grinded AR-35

SEM was used to observe the microstructure surface of the sample. The sample was lying on a double-sided tape and thin strips of conductive copper sheet were placed in some places. Subsequently was spattering gold to ensure good electrical conductivity. Finally the sample was observed by Zeiss Sigma 300 SEM electron microscope (FEG Oberkochen, Germany) in backscattered (AsB) mode.[4]

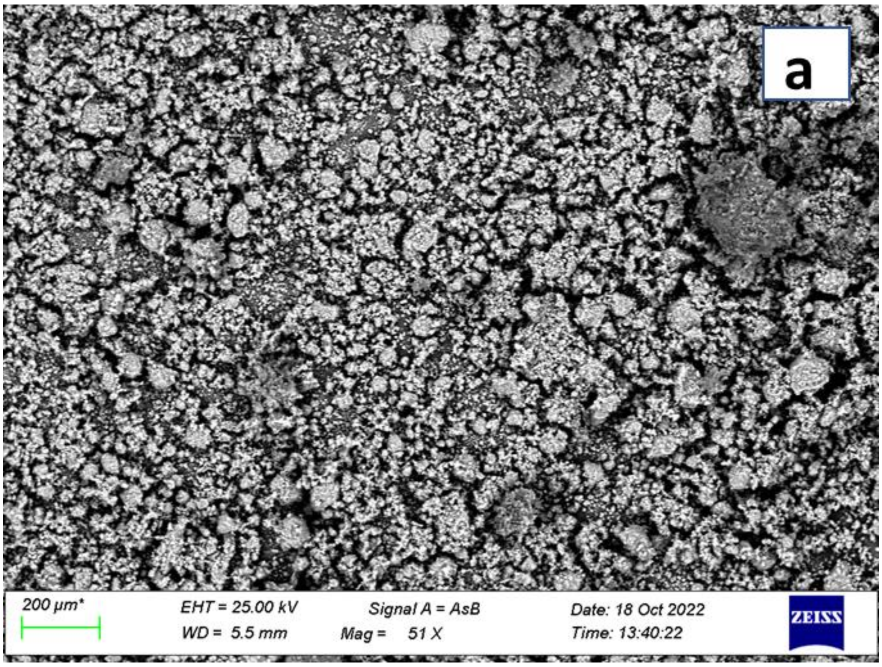

Figure S9. (51X) Microstructure overview of sample AR-35 after ball mill grinding. To better analyse the structures of the sample AsB backscattered method was used.

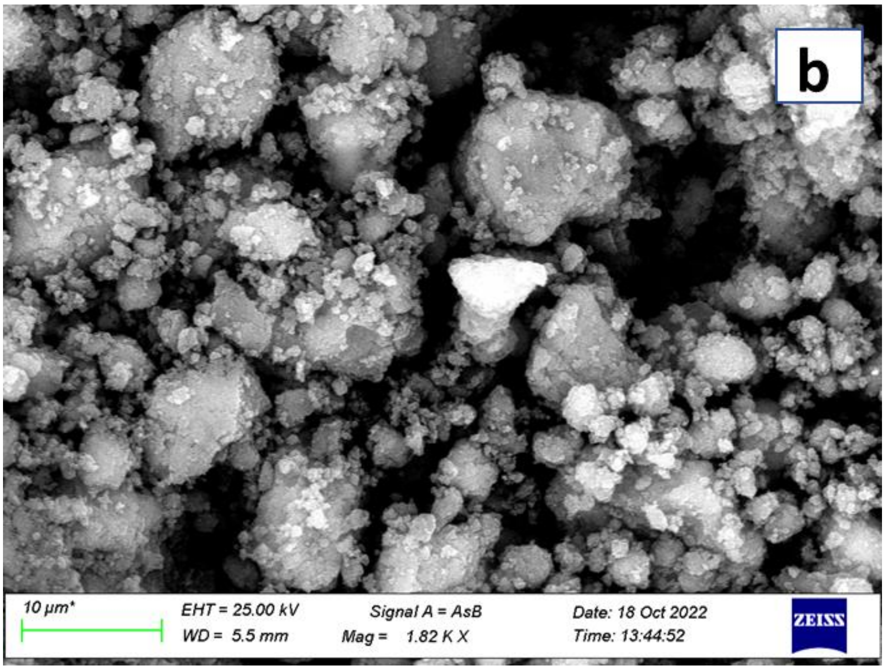

Figure S10. (1800X) Microstructure overview of sample AR-35. After ball mill grinding. To better analyse the structures of the sample AsB backscattered method was used.

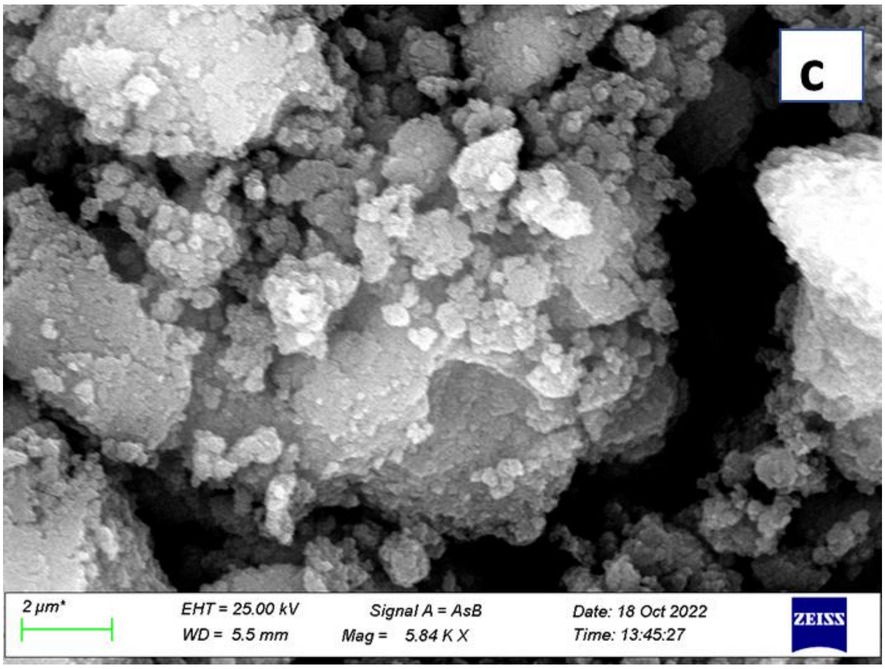

Figure S11. (5800X) The image shows a detail about the field displayed in figure S9.

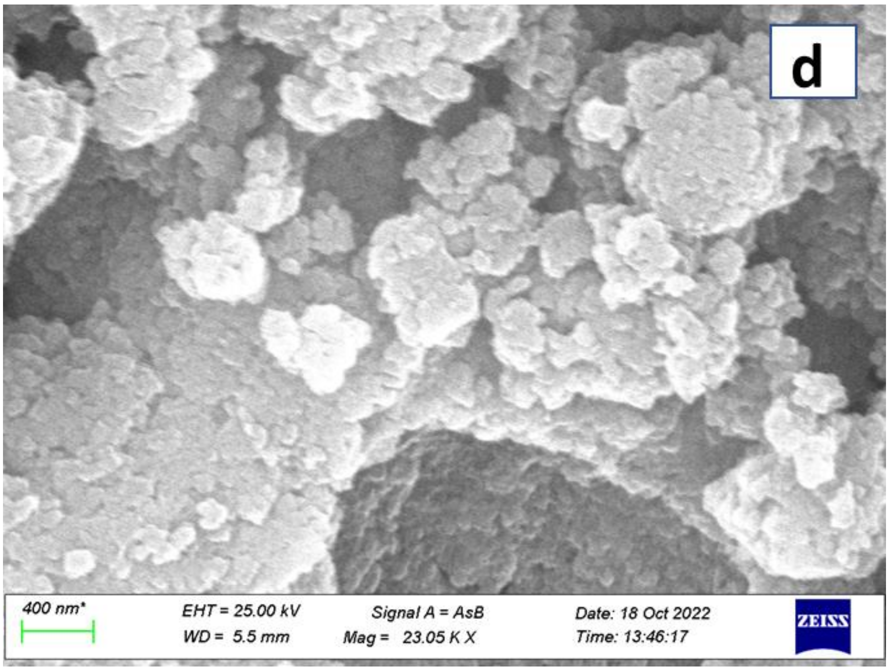

Figure S12. (23000) The image shows a detail about the field displayed in figure S10.

6. AR-35 columns performance testing

In order to evaluate the reuse and reconditioning of the columns packed with AR-35, once used for 10 hours, they were connected to an acid corrosion resistant HPLC pump which dispensed 50 ml of a 10% solution. in THF 1mL / min (900 psi) of methane sulfonic acid. Then, pure THF was flushed for 60 minutes. After this treatment, the columns were used to evaluate their catalytic performance in two other runs.

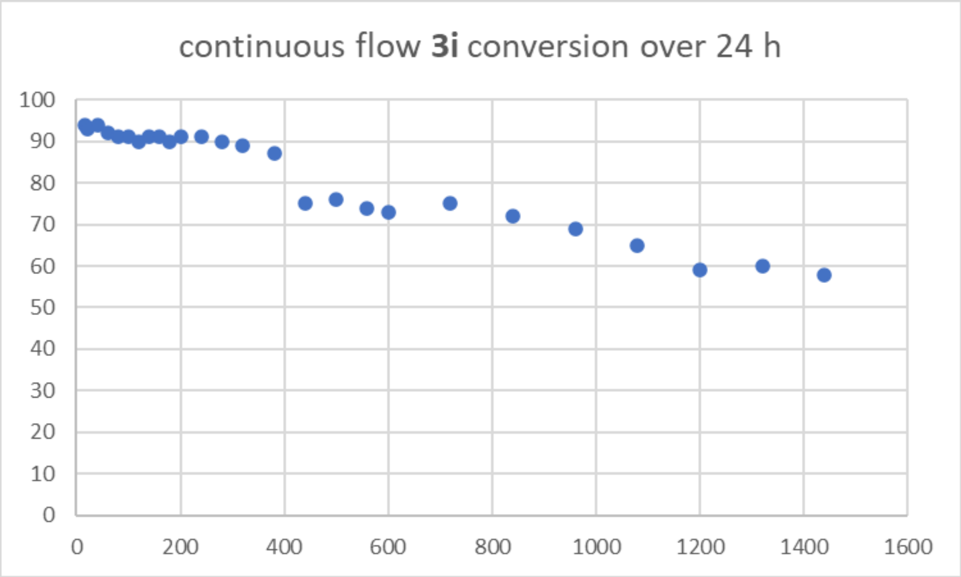

Figure S13. determination of the conversion of 3i under continuous flow conditions using fleshly grinded AR-35.

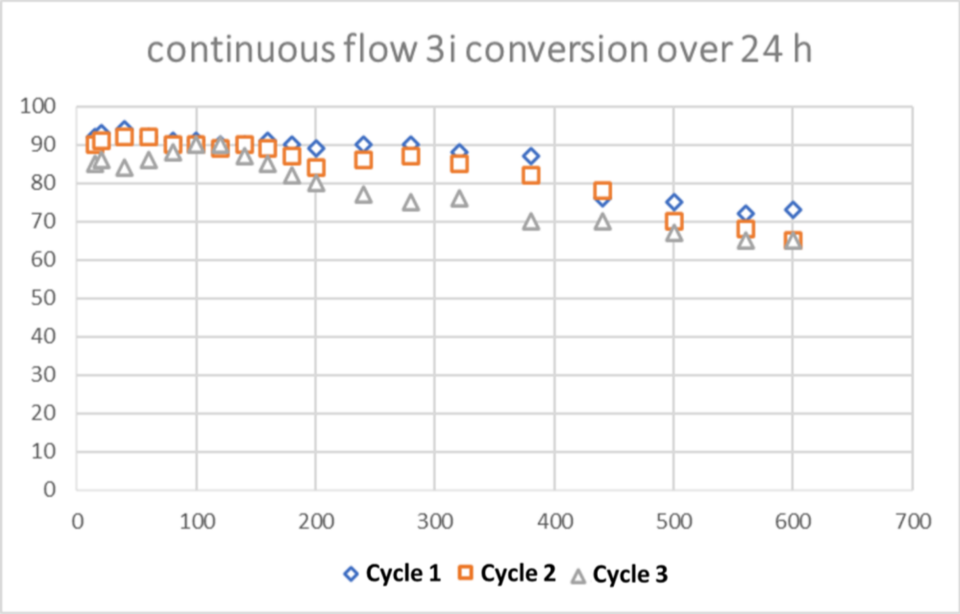

Figure S14. Comparison of the conversions for compound **3i** under continuous flow conditions using fleshly grinded AR-35 over ten hours (cycle 1, blue), reconditioned AR-35 (cycles 2 orange, and cycle 3, grey).

TON and TOF calculation

The TON was calculated taking into consideration experiments lasting 24h in which the conversion of **1b** to **3i** (M.W. 206g / mol) ranged from> 93% to approx. 70-75%. In these experiments an average quantity of product equal to 32 g was obtained, which correspond to about 155 mmol/24h.[5]

$$\text{TON} = \frac{mmol\ \text{of}\ \mathbf{3i}}{(acid\ sites\ by\ gram\ of\ catalyst)\ x\ (gram\ of\ catalyst)} = \frac{155\ mmol\ \text{of}\ \mathbf{3i}}{63.60} = 2.43$$

$$\text{TOF} = \frac{\text{TON}}{Time\ (min)} = \frac{2.43}{1440} = = 0.0017$$

7. NMR characterization of compounds 3-7

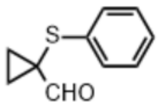

3a 1-(phenylthio)cyclopropanecarbaldehyde

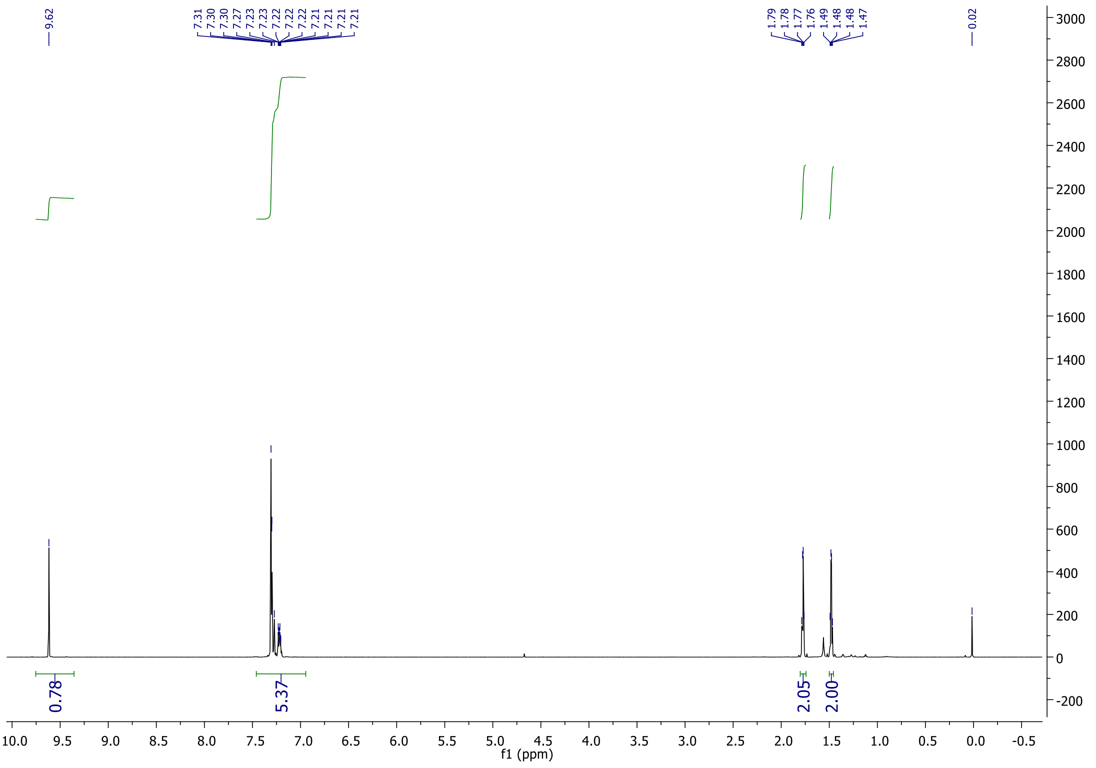

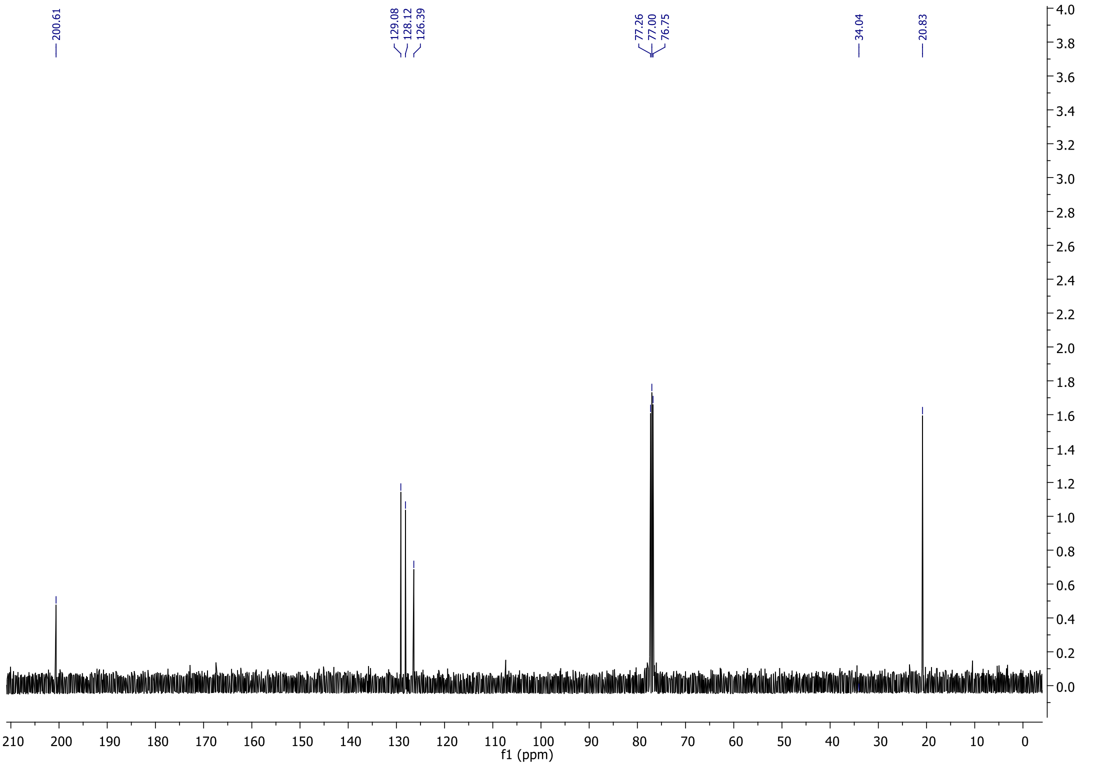

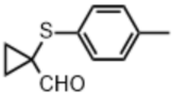

3b 1-(*p*-tolylthio)cyclopropanecarbaldehyde

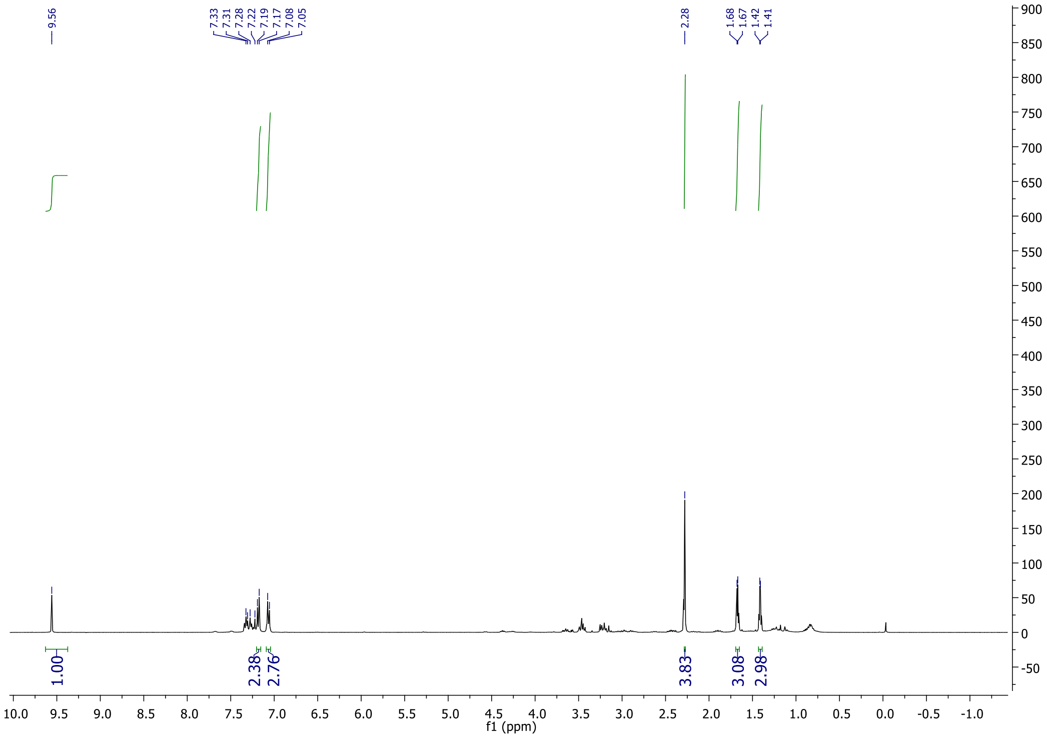

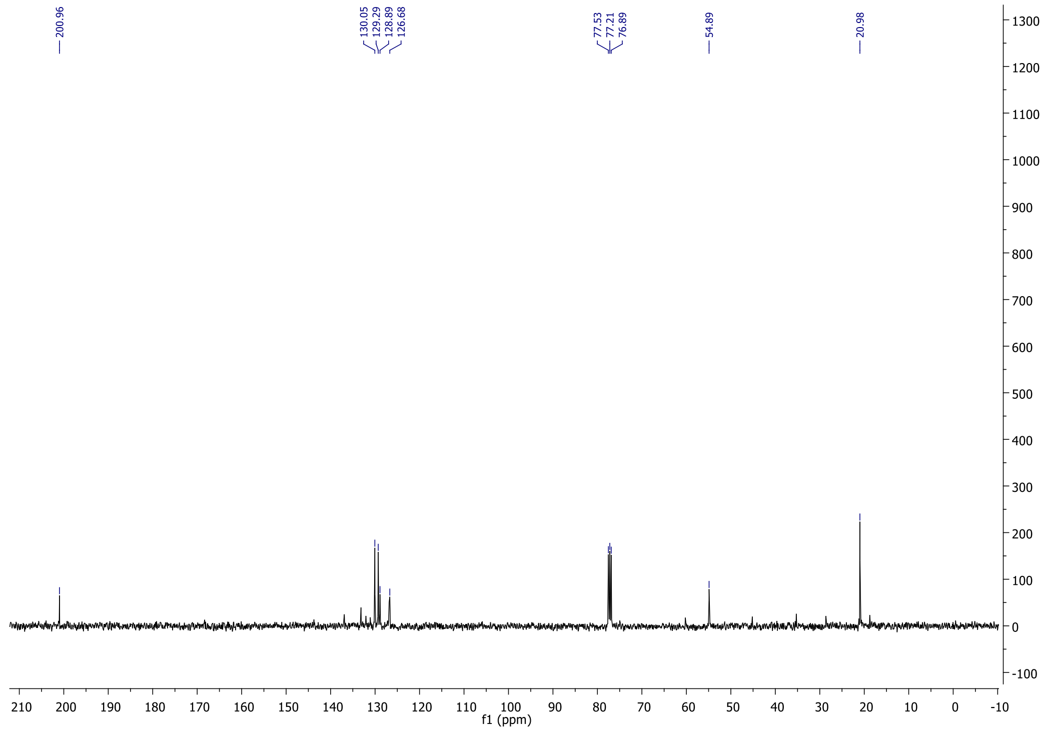

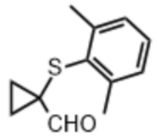

3c 1-((2,6-dimethylphenyl)thio)cyclopropanecarbaldehyde

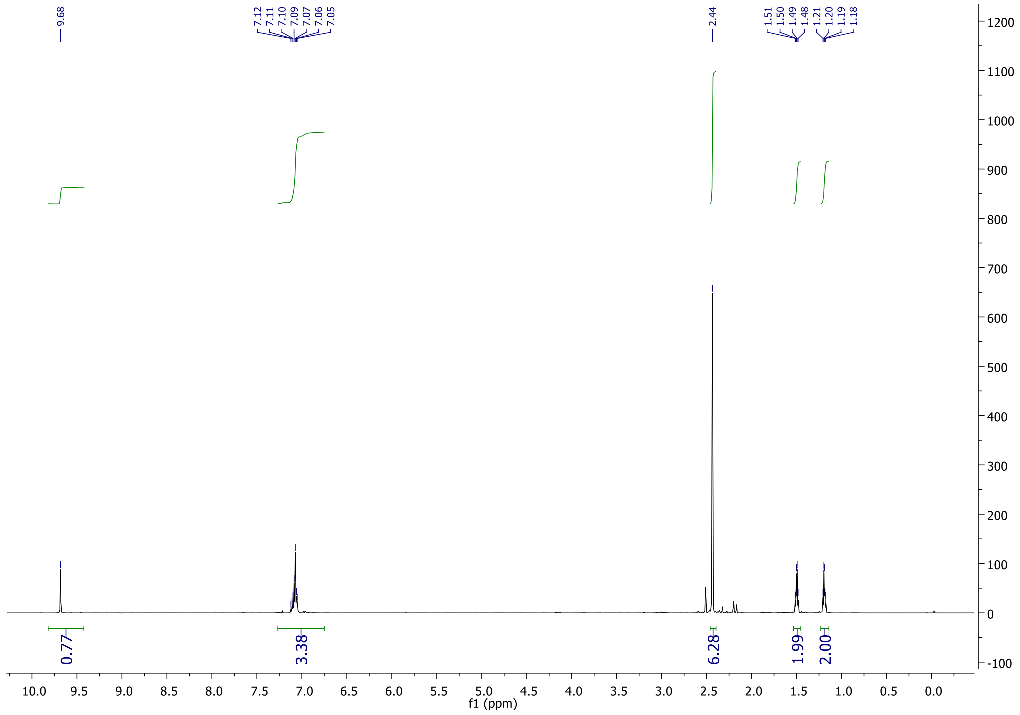

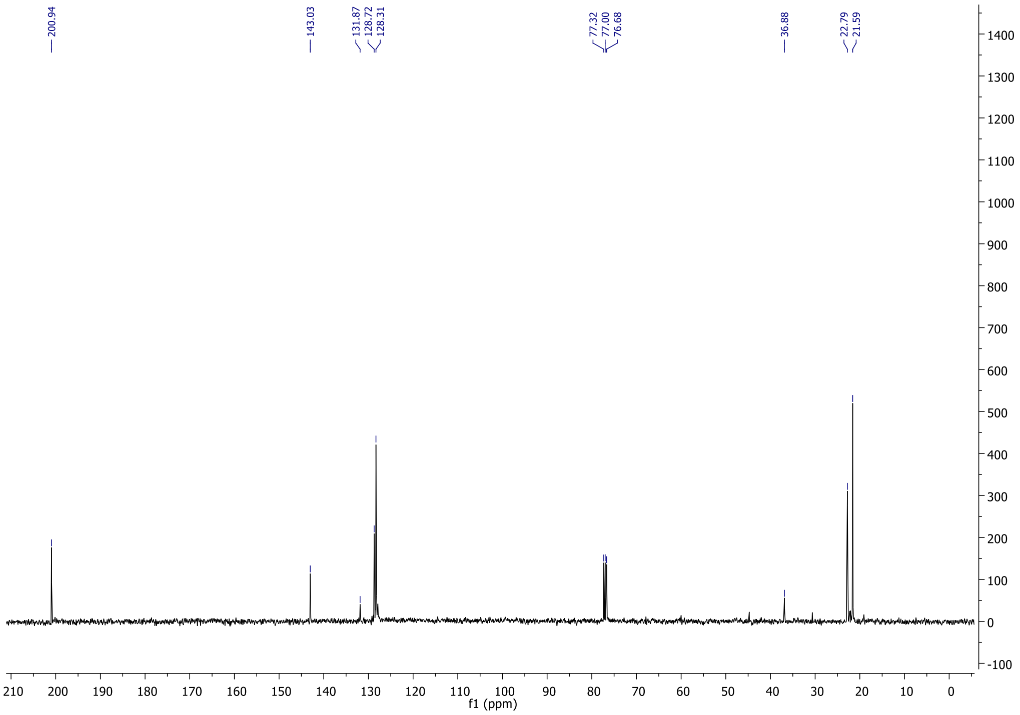

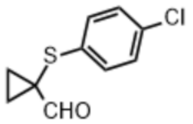

3d 1-((4-chlorophenyl)thio)cyclopropanecarbaldehyde

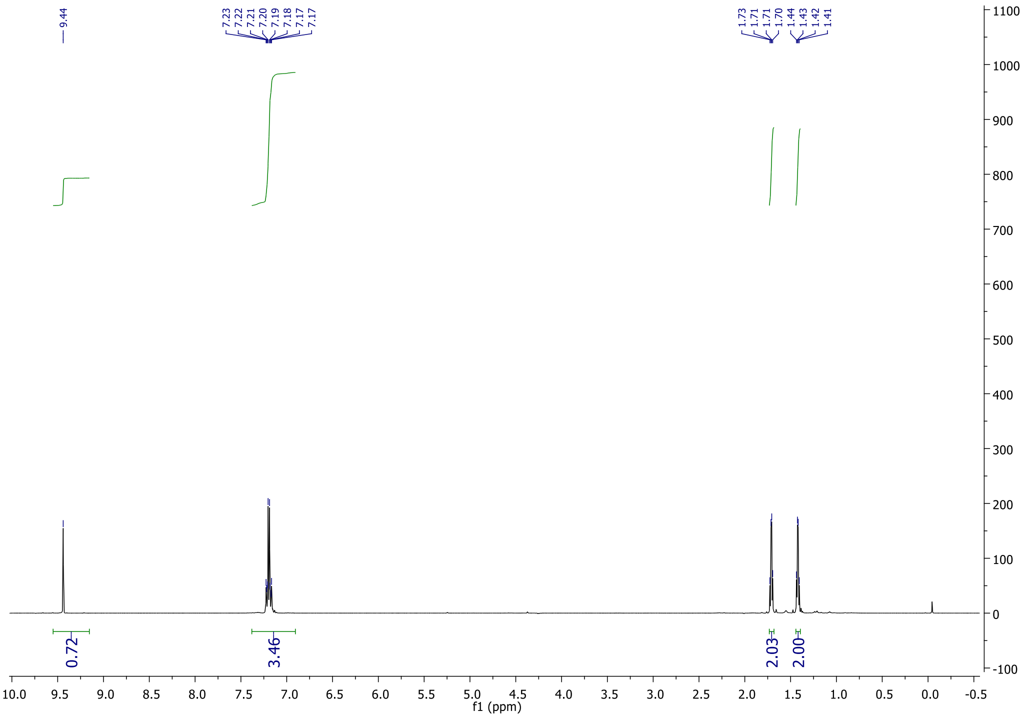

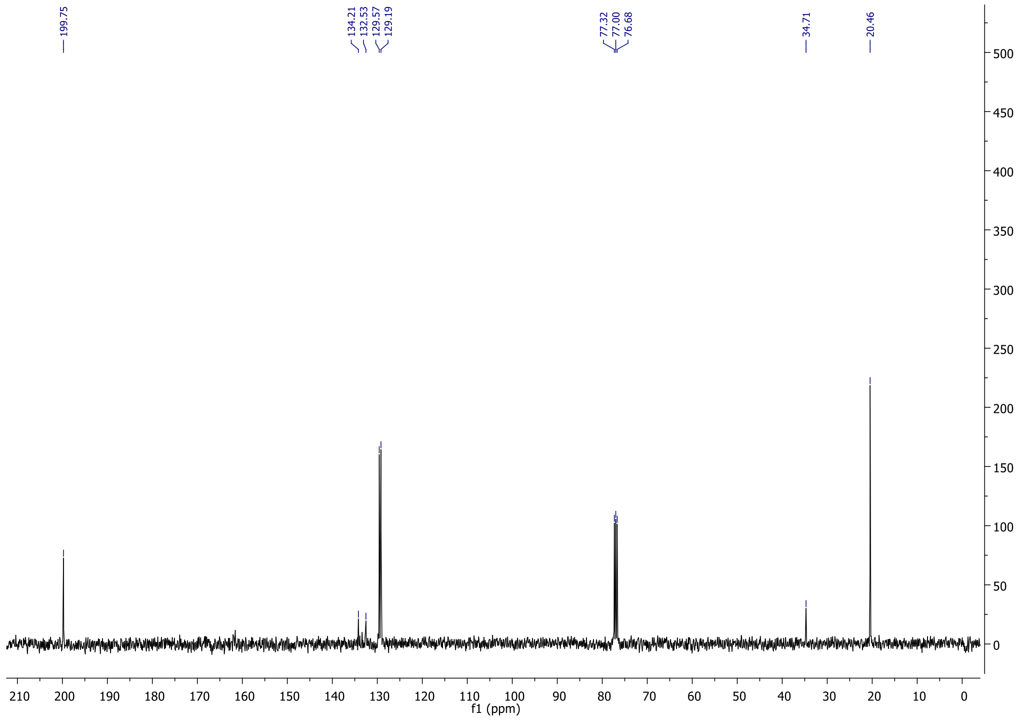

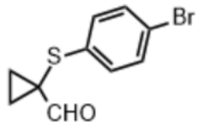

3e 1-((4-bromophenyl)thio)cyclopropanecarbaldehyde

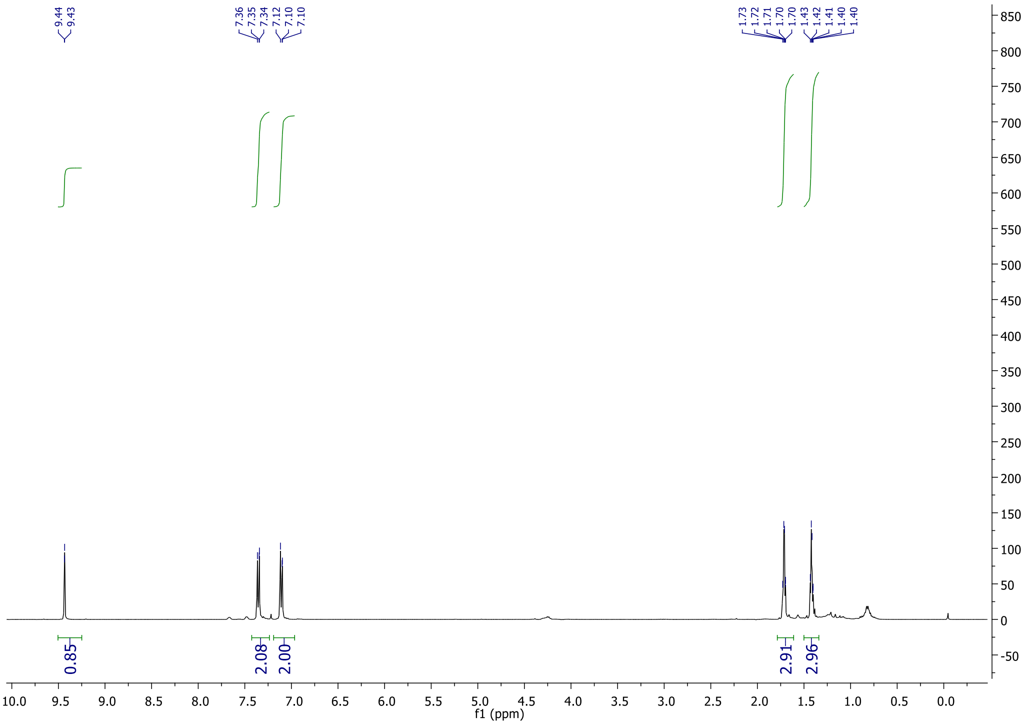

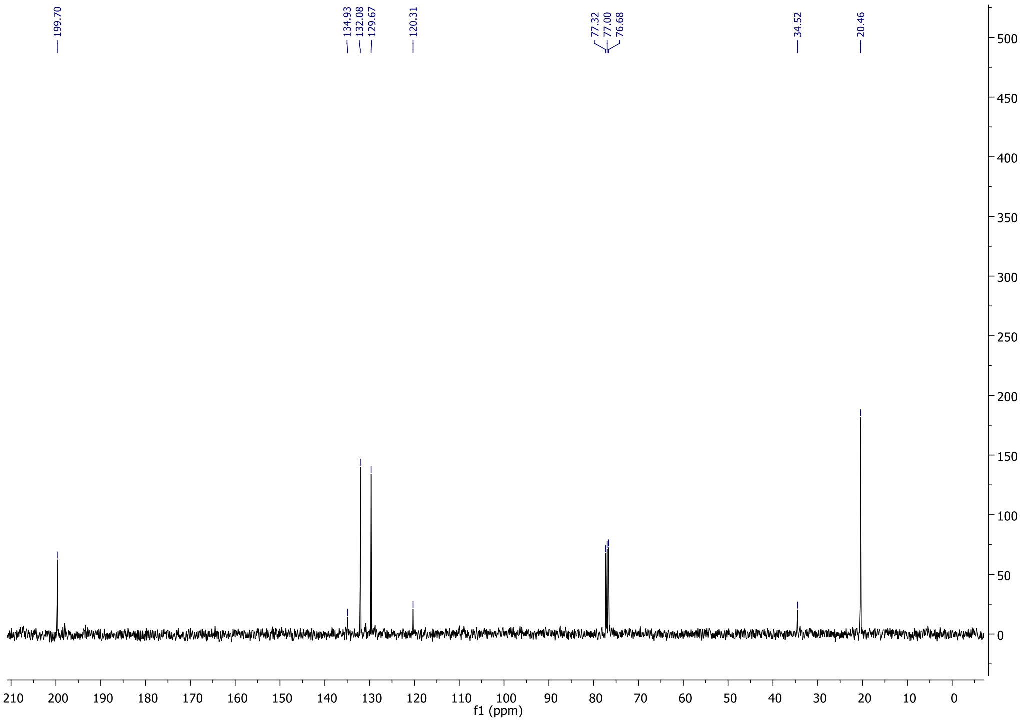

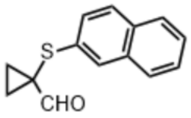

3f 1-(naphthalen-2-ylthio)cyclopropanecarbaldehyde

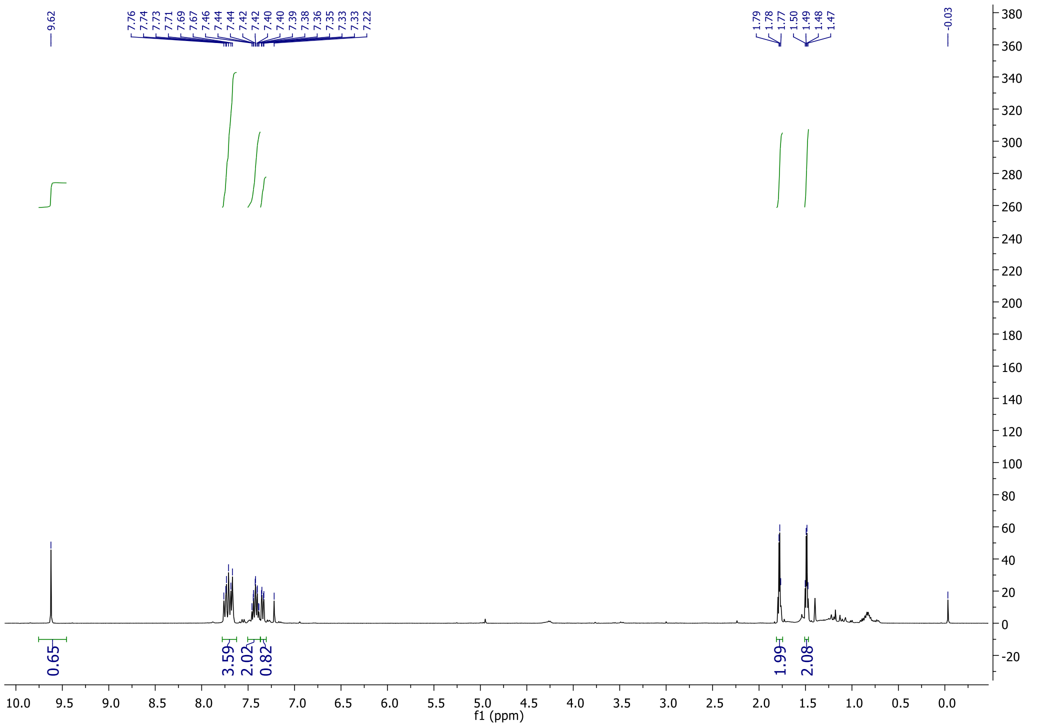

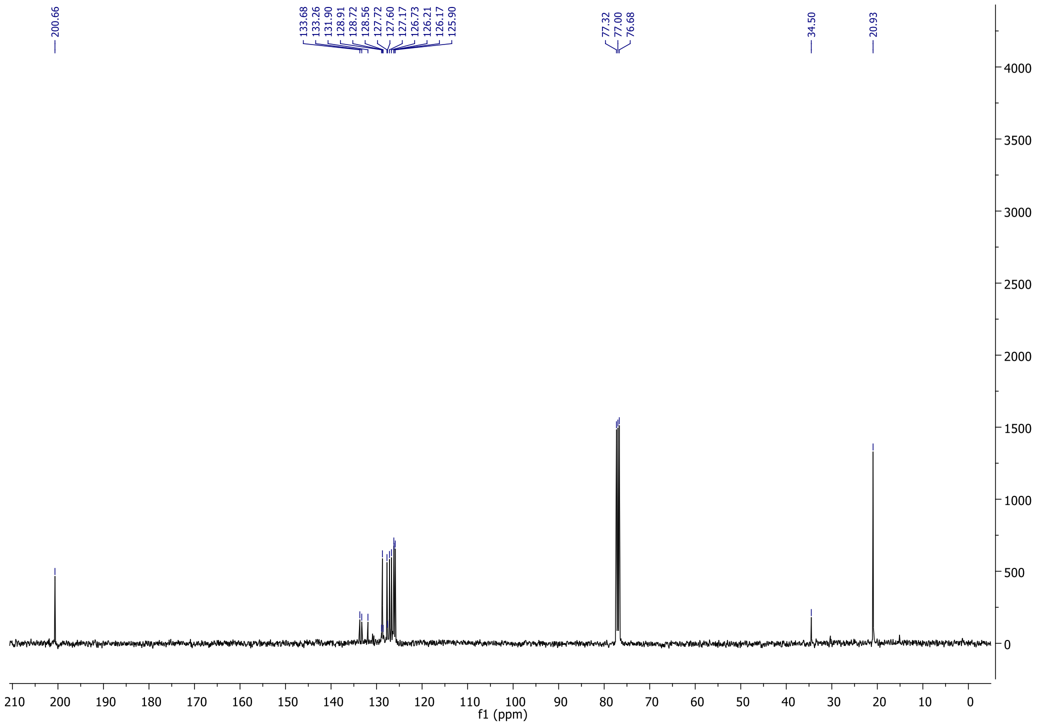

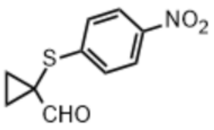

3g 1-((4-nitrophenyl)thio)cyclopropanecarbaldehyde

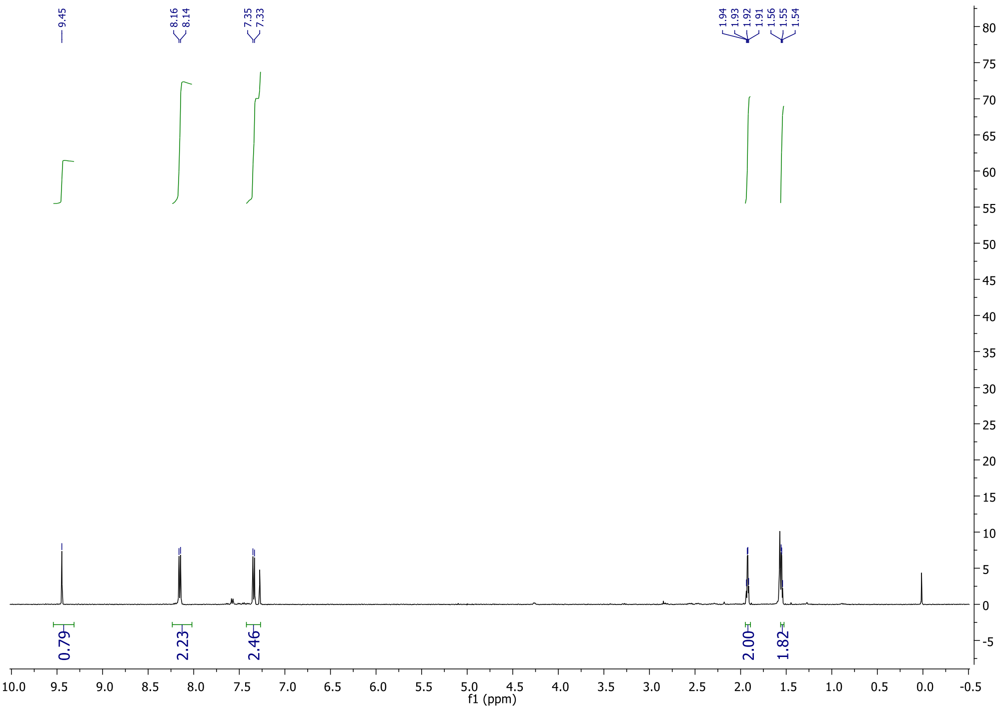

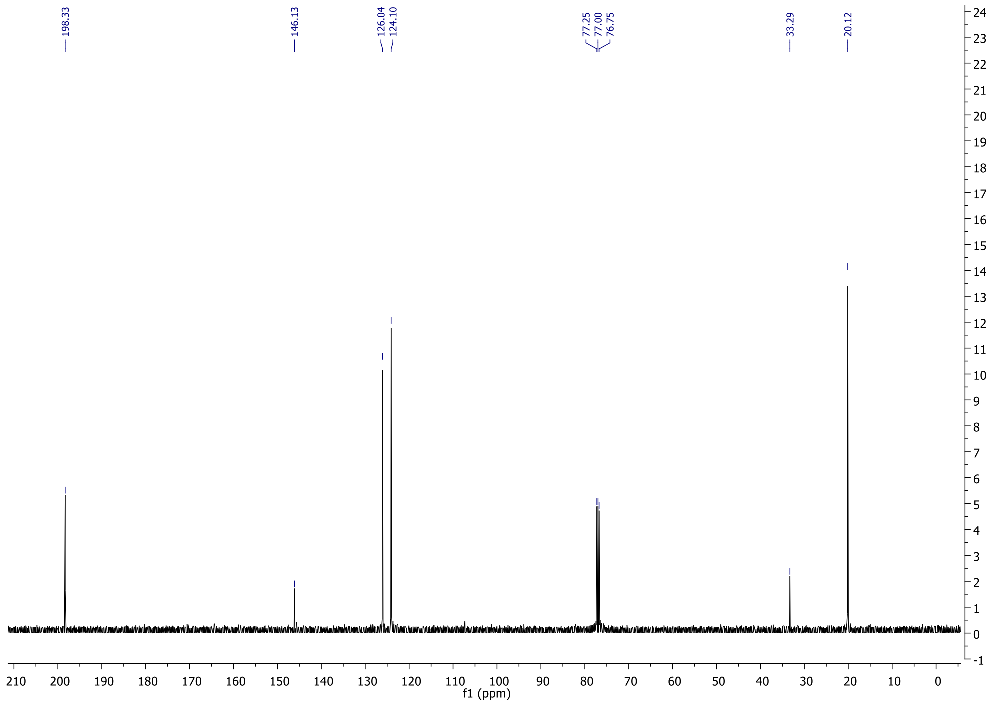

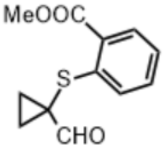

3h methyl 2-((1-formylcyclopropyl)thio)benzoate

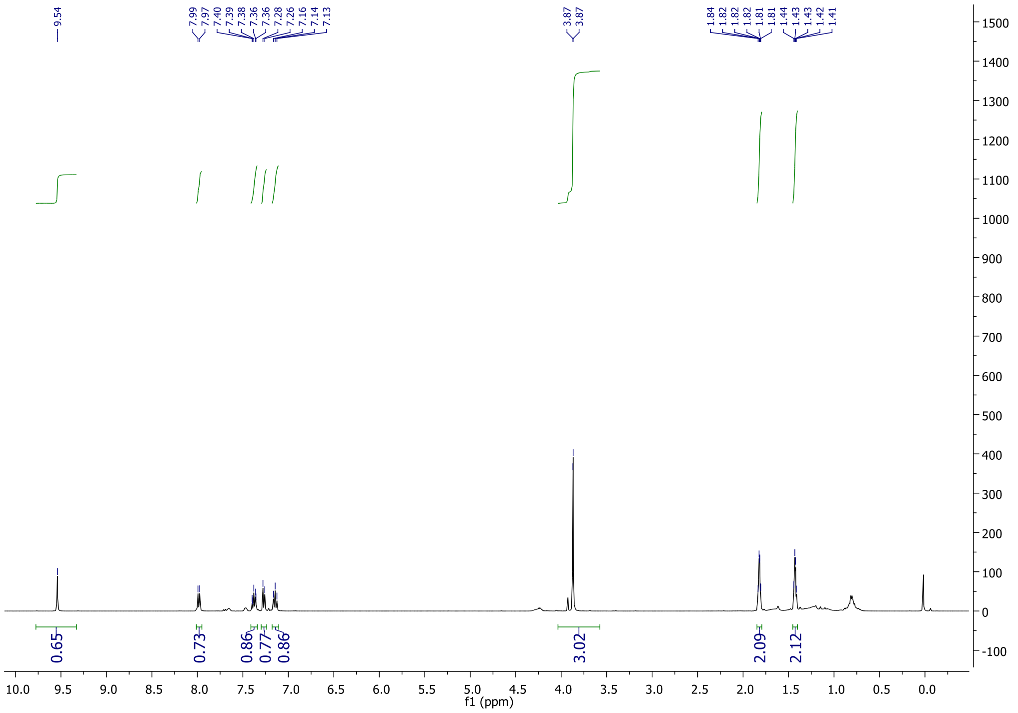

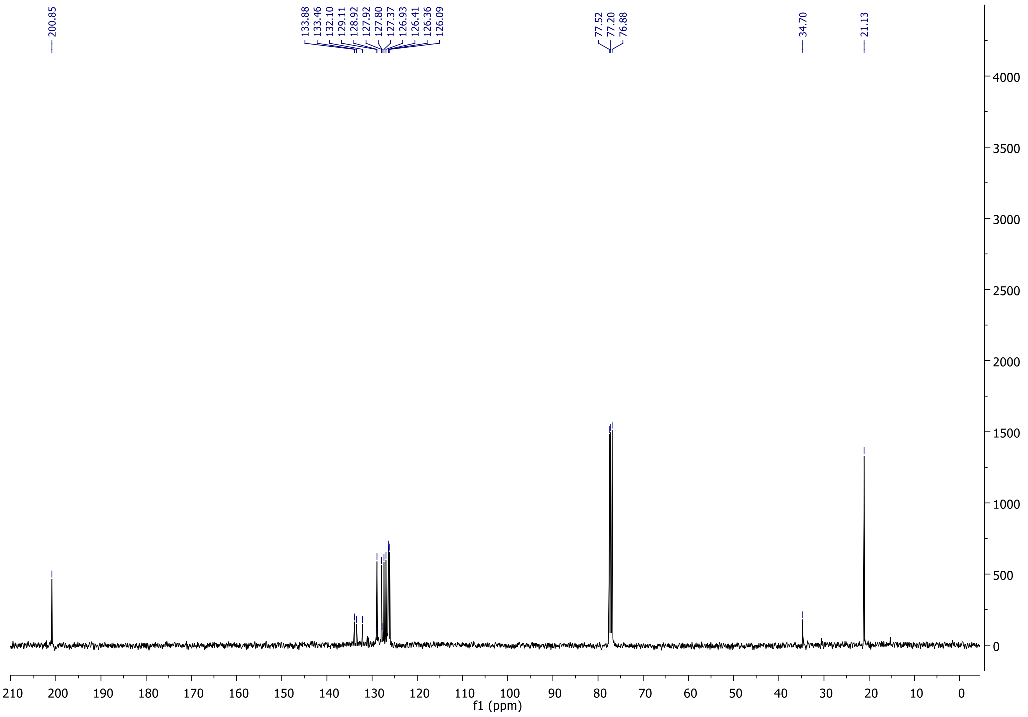

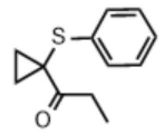

3j 1-(1-(phenylthio)cyclopropyl)propan-1-one

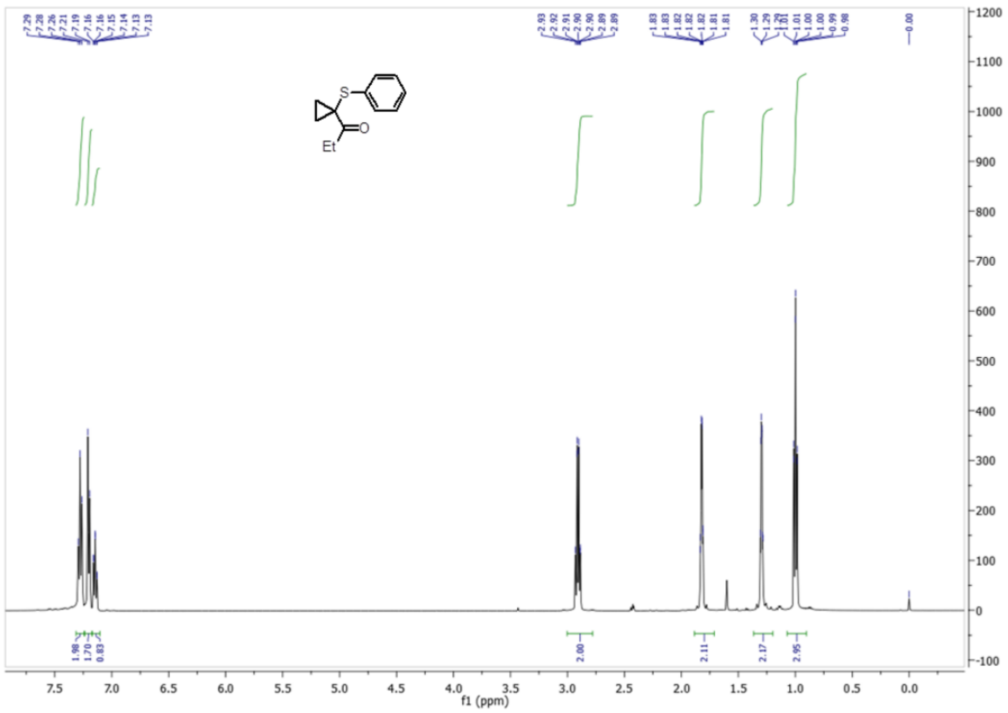

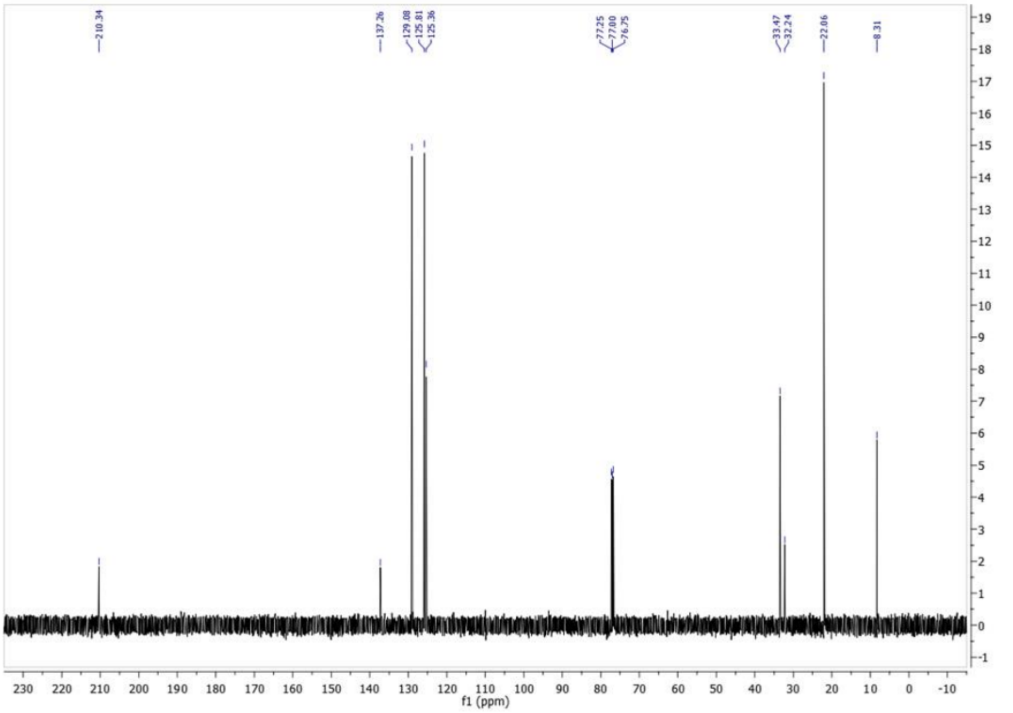

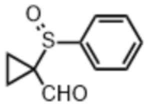

4. 1-(phenylsulfinyl)cyclopropanecarbaldehyde

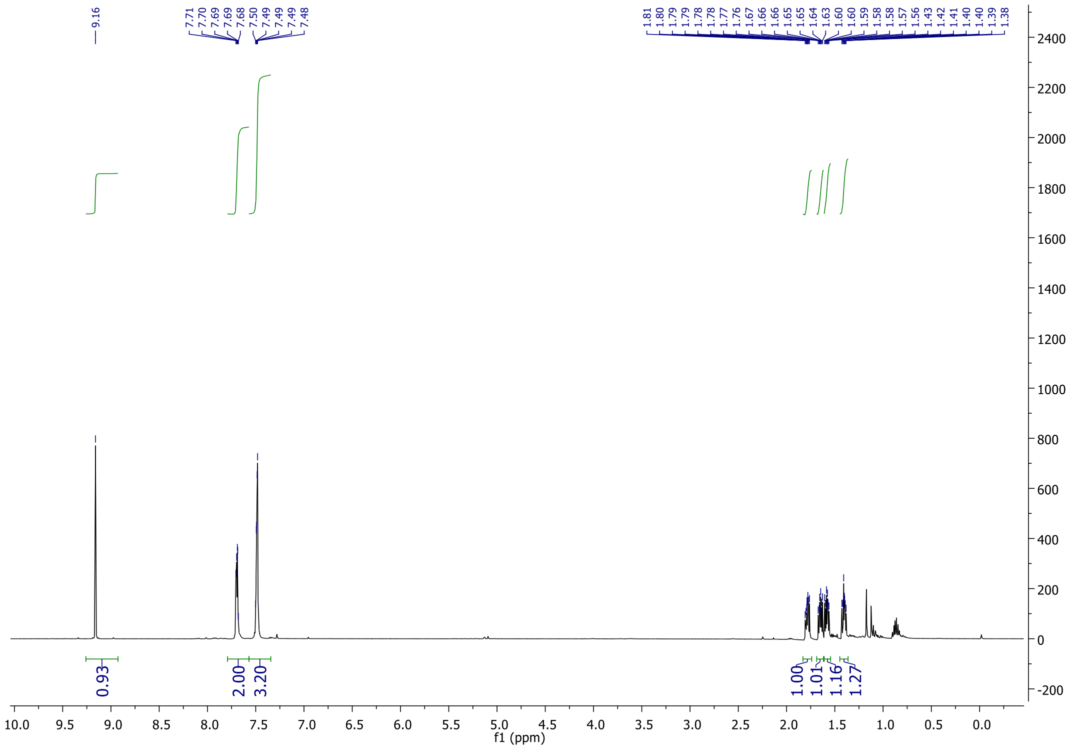

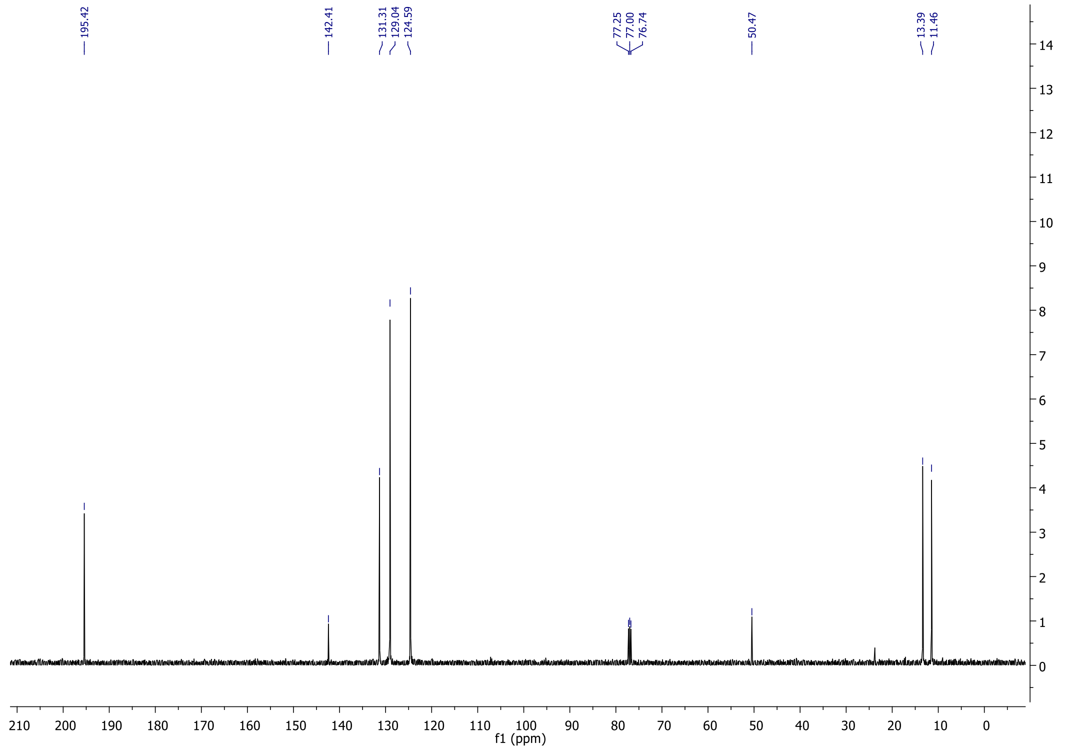

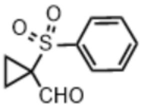

5 1-(phenylsulfonyl)cyclopropanecarbaldehyde

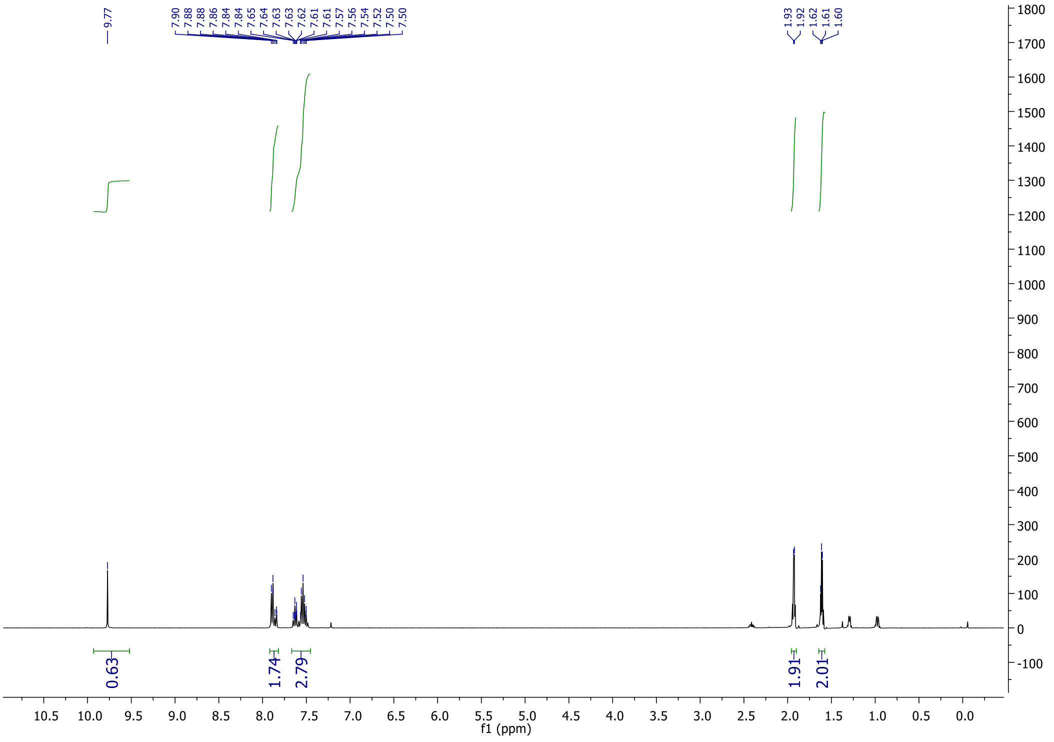

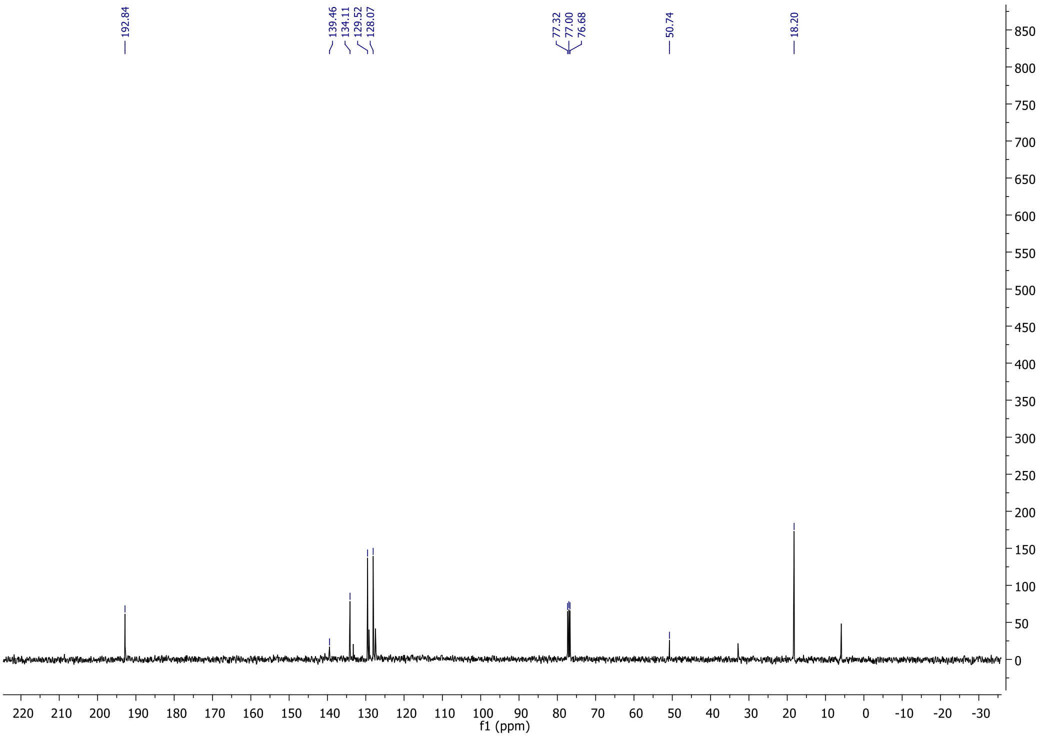

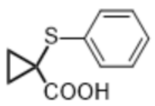

6 1-(phenylthio)cyclopropanecarboxylic acid

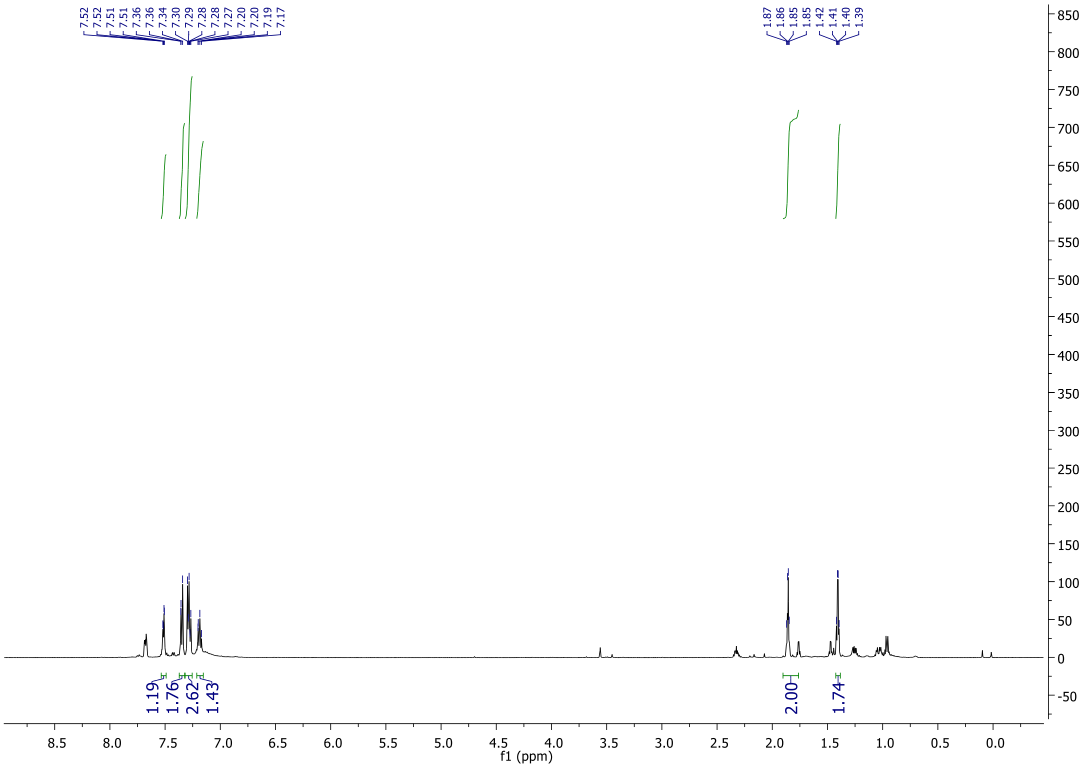

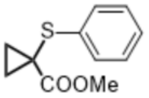

7 methyl 1-(phenylthio)cyclopropanecarboxylate

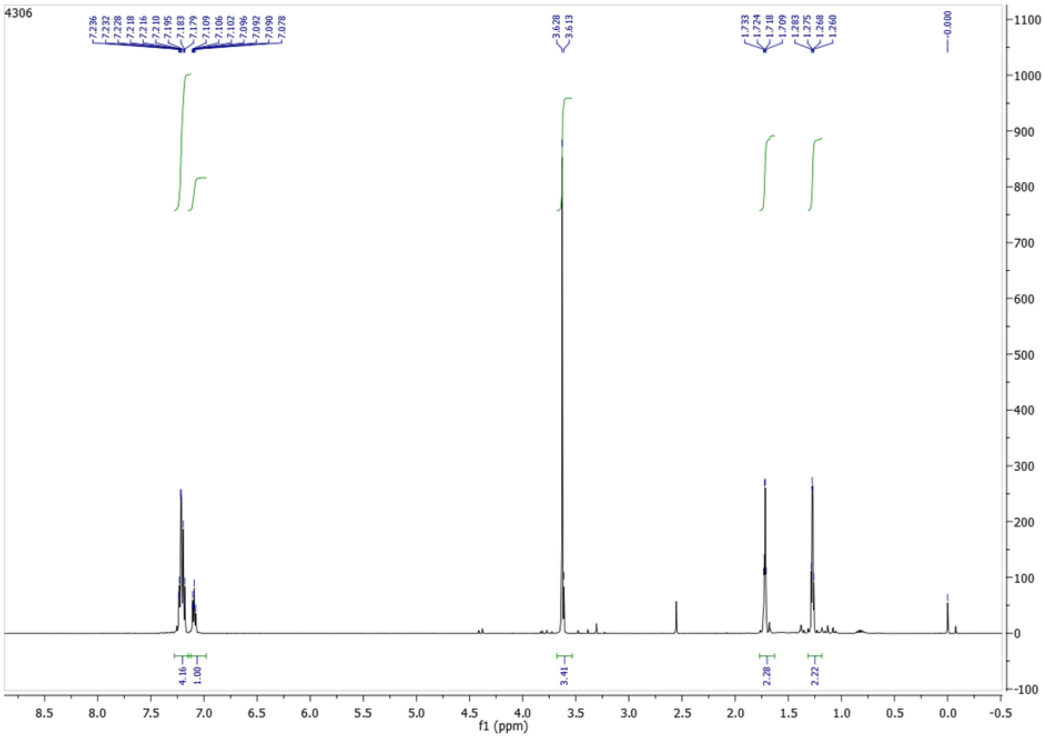

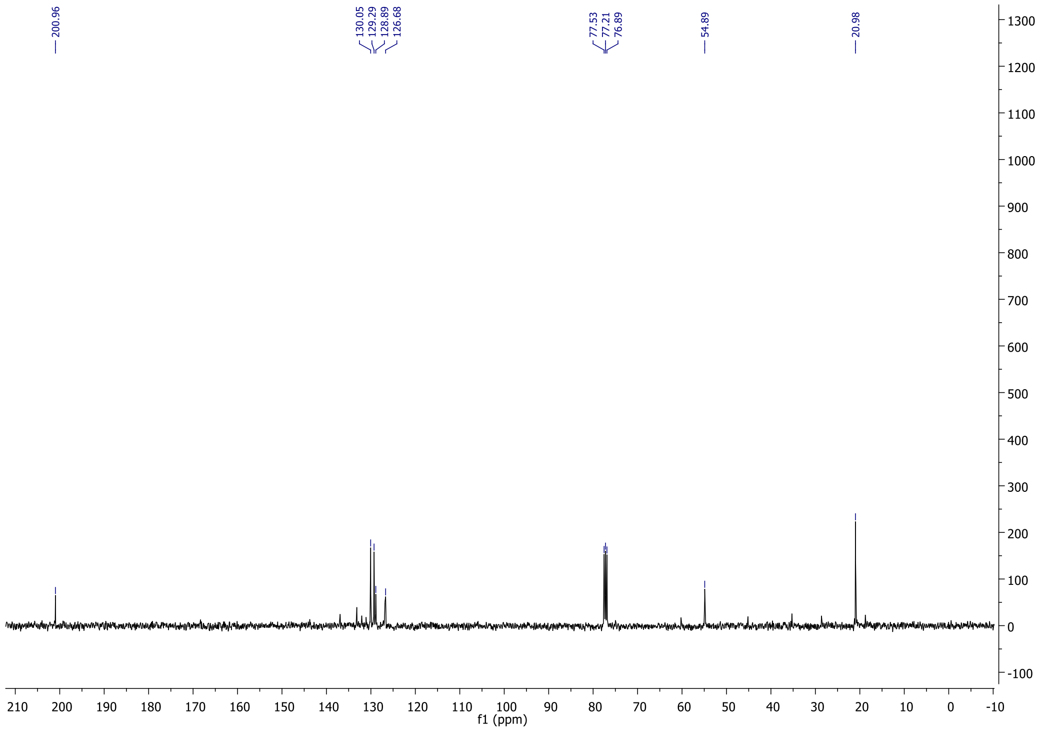

8. References and notes

1) Kazakevich, Y. V.; Lobrutto, R. *HPLC for Pharmaceutical Scientists*; John Wiley & Sons, Inc.: Hoboken, New Jersey, 2007
2) P.F. Siril, H.E. Cross, D.R. Brown*, J. Mol. Catal. A: Chem*., 2008, 279, 63-68.
3) Mu. Naushad, Z.A. ALOthman, M.R. Khana, N.J. ALQahtani, I.H. ALSohaimi. *J. Industrial Eng. Chem.,* **2014**, 20, 4393-4400.
4) S. Jin Oh, J. Park, J.G. Na, Y.K. Oh, Y.K. Chang, *RSC Adv*., **2015**, 5, 47983-47989.
5) S. Ye, C. Ding, C. Li, *Adv. Inorg. Chem*., **2019**, *74*, 2019, 3-59.

micromeritics®

Sample: campione Cagliari macinato
Operator:
Submitter:
File: C:\ASAP 2020 Plus\data\Chiappone\2022-1...\000-223.SMP

Started:	19/10/2022 11:35:41	Analysis adsorptive:	N2
Completed:	19/10/2022 16:31:25	Analysis bath temp.:	77,188 K
Report time:	20/10/2022 18:25:04	Thermal correction:	No
Sample mass:	0,3526 g	Ambient free space:	15,7578 cm³ Measured
Analysis free space:	45,2933 cm³	Equilibration interval:	10 s
Low pressure dose:	None	Sample density:	1,000 g/cm³
Automatic degas:	Yes		

Sample prep: Stage	Temperature (°C)	Ramp Rate (°C/min)	Time (min)
1	30	10	10

Summary Report

Surface Area

Single point surface area at p/p° = 0,249708467: 4,6473 m²/g

BET Surface Area: 4,8426 m²/g

Langmuir Surface Area: 44,3943 m²/g

t-Plot Micropore Area: 0,0744 m²/g

t-Plot external surface area: 4,7682 m²/g

BJH Adsorption cumulative surface area of pores between 1,7000 nm and 300,0000 nm width: 4,0304 m²/g

BJH Desorption cumulative surface area of pores between 1,7000 nm and 300,0000 nm width: 3,9755 m²/g

D-H Adsorption cumulative surface area of pores between 1,7000 nm and 300,0000 nm width: 3,9505 m²/g

D-H Desorption cumulative surface area of pores between 1,7000 nm and 300,0000 nm width: 3,8925 m²/g

Pore Volume

Single point adsorption total pore volume of pores less than 40,3122 nm width at p/p° = 0,950000000: 0,010168 cm³/g

Single point desorption total pore volume of pores less than 40,3122 nm width at p/p° = 0,950000000: 0,014914 cm³/g

t-Plot micropore volume: -0,000020 cm³/g

BJH Adsorption cumulative volume of pores between 1,7000 nm and 300,0000 nm width: 0,037288 cm³/g

BJH Desorption cumulative volume of pores between 1,7000 nm and 300,0000 nm width: 0,037100 cm³/g

D-H Adsorption cumulative volume of pores between 1,7000 nm and 300,0000 nm width: 0,037118 cm³/g

D-H Desorption cumulative volume of pores

Sample: campione Cagliari macinato
Operator:
Submitter:
File: C:\ASAP 2020 Plus\data\Chiappone\2022-1...\000-223.SMP

Started:	19/10/2022 11:35:41	Analysis adsorptive:	N2
Completed:	19/10/2022 16:31:25	Analysis bath temp.:	77,188 K
Report time:	20/10/2022 18:25:04	Thermal correction:	No
Sample mass:	0,3526 g	Ambient free space:	15,7578 cm³ Measured
Analysis free space:	45,2933 cm³	Equilibration interval:	10 s
Low pressure dose:	None	Sample density:	1,000 g/cm³
Automatic degas:	Yes		

Sample prep: Stage	Temperature (°C)	Ramp Rate (°C/min)	Time (min)
1	30	10	10

Pore Volume

between 1,7000 nm and 300,0000 nm width: 0,036903 cm³/g

Pore Size

Adsorption average pore diameter (4V/A by BET): 8,3986 nm

Desorption average pore diameter (4V/A by BET): 12,3194 nm

BJH Adsorption average pore width (4V/A): 37,0067 nm

BJH Desorption average pore width (4V/A): 37,3295 nm

D-H Adsorption average pore width (4V/A): 37,5833 nm

D-H Desorption average pore width (4V/A): 37,9220 nm

Freundlich

Qm·C: 0,0882 ± 0,0207 cm³/g STP

m: 1,6853 ± 0,2715

Temkin

q·alpha/Qm: 0,280312 ± 0,096339 kJ/mol·(cm³/g STP)

A: 0,0218 ± 0,0512 mmHg

Nanoparticle Size:

Average Particle Size 1.239,0035 nm

Horvath-Kawazoe

Maximum pore volume at p/p° = 0,179888289: 0,002005 cm³/g

Median pore width: 0,7889 nm

MP-Method

Cumulative surface area of pores between
1,96327 nm and 1,96327 nm hydraulic radius: 0,0000 m²/g

Cumulative pore volume of pores between

Sample: campione Cagliari macinato
Operator:
Submitter:
File: C:\ASAP 2020 Plus\data\Chiappone\2022-1...\000-223.SMP

Started:	19/10/2022 11:35:41	Analysis adsorptive:	N2
Completed:	19/10/2022 16:31:25	Analysis bath temp.:	77,188 K
Report time:	20/10/2022 18:25:04	Thermal correction:	No
Sample mass:	0,3526 g	Ambient free space:	15,7578 cm³ Measured
Analysis free space:	45,2933 cm³	Equilibration interval:	10 s
Low pressure dose:	None	Sample density:	1,000 g/cm³
Automatic degas:	Yes		

Sample prep: Stage	Temperature (°C)	Ramp Rate (°C/min)	Time (min)
1	30	10	10

MP-Method

1,96327 nm and 1,96327 nm hydraulic radius: 0,000000 cm³/g

Average pore hydraulic radius (V/A): 0,00000 nm

Sample: campione Cagliari macinato
Operator:
Submitter:
File: C:\ASAP 2020 Plus\data\Chiappone\2022-1...\000-223.SMP

Started:	19/10/2022 11:35:41	Analysis adsorptive:	N2
Completed:	19/10/2022 16:31:25	Analysis bath temp.:	77,188 K
Report time:	20/10/2022 18:25:04	Thermal correction:	No
Sample mass:	0,3526 g	Ambient free space:	15,7578 cm³ Measured
Analysis free space:	45,2933 cm³	Equilibration interval:	10 s
Low pressure dose:	None	Sample density:	1,000 g/cm³
Automatic degas:	Yes		

Sample prep: Stage	Temperature (°C)	Ramp Rate (°C/min)	Time (min)
1	30	10	10

Isotherm Tabular Report

Relative Pressure (p/p°)	Absolute Pressure (mmHg)	Quantity Adsorbed (cm³/g STP)	Elapsed Time (h:min)	Saturation Pressure (mmHg)
			01:31	747.010742
0.011463180	8.555197	0.6789	01:49	746.319702
0.033786418	25.212904	0.8733	01:52	746.243774
0.068677282	51.249722	1.0232	01:54	746.239807
0.079940468	59.651485	1.0618	01:56	746.198853
0.100049911	74.653831	1.1201	01:59	746.165894
0.120504693	89.912277	1.1715	02:01	746.130920
0.140480737	104.813515	1.2151	02:03	746.105957
0.159878107	119.280426	1.2582	02:05	746.071045
0.179888289	134.200638	1.2963	02:07	746.022095
0.200381282	149.487457	1.3365	02:10	746.015076
0.249708467	186.282547	1.4231	02:12	746.000122
0.300568445	224.212997	1.5032	02:14	745.963196
0.350704638	261.594879	1.5874	02:16	745.912231
0.401172050	299.235931	1.6693	02:18	745.904236
0.451228599	336.567017	1.7547	02:21	745.890259
0.501302383	373.881531	1.8446	02:23	745.820374
0.551384683	411.206940	1.9417	02:25	745.771423
0.601216792	448.366119	2.0440	02:27	745.764465
0.651562704	485.951324	2.1607	02:30	745.824341
0.701638381	523.206482	2.2953	02:32	745.692505
0.751620244	560.521118	2.4638	02:34	745.750427
0.801605580	597.780090	2.6811	02:37	745.728455
0.821077936	612.285645	2.8020	02:39	745.709534
0.851339039	634.834595	3.0346	02:41	745.689514
0.875950060	653.130798	3.3207	02:43	745.625610
0.900785272	671.631470	3.7930	02:46	745.606628
0.925324796	689.895996	4.6745	02:49	745.571716
0.949165249	707.694458	6.4900	02:52	745.596680
0.971491871	724.288696	10.5363	02:58	745.542725
0.981754511	731.840942	14.2059	03:04	745.441895
0.990077856	737.964417	19.1602	03:11	745.359985
0.994727279	741.368286	24.3458	03:19	745.298035
0.985858928	734.685913	20.6075	03:25	745.224182
0.977057333	728.027222	16.5585	03:31	745.122314
0.968199114	721.408386	13.4947	03:37	745.103333
0.948730941	706.843811	9.4527	03:44	745.041382
0.927893090	691.310425	6.9240	03:48	745.032410
0.902313792	672.220581	5.1545	03:52	744.996460
0.876533438	652.990662	4.1393	03:55	744.969482

Sample: campione Cagliari macinato
Operator:
Submitter:
File: C:\ASAP 2020 Plus\data\Chiappone\2022-1...\000-223.SMP

Started:	19/10/2022 11:35:41	Analysis adsorptive:	N2
Completed:	19/10/2022 16:31:25	Analysis bath temp.:	77,188 K
Report time:	20/10/2022 18:25:04	Thermal correction:	No
Sample mass:	0,3526 g	Ambient free space:	15,7578 cm³ Measured
Analysis free space:	45,2933 cm³	Equilibration interval:	10 s
Low pressure dose:	None	Sample density:	1,000 g/cm³
Automatic degas:	Yes		

Sample prep: Stage	Temperature (°C)	Ramp Rate (°C/min)	Time (min)
1	30	10	10

Isotherm Tabular Report

Relative Pressure (p/p°)	Absolute Pressure (mmHg)	Quantity Adsorbed (cm³/g STP)	Elapsed Time (h:min)	Saturation Pressure (mmHg)
0.850921480	633.879944	3.5321	03:57	744.933533
0.825097887	614.635681	3.1572	04:00	744.924561
0.799824169	595.811890	2.9126	04:02	744.928589
0.748994636	557.941528	2.6143	04:04	744.920593
0.699390626	520.963928	2.4242	04:06	744.882629
0.649343422	483.685944	2.2770	04:08	744.884644
0.599136154	446.272949	2.1556	04:11	744.860657
0.549312877	409.151123	2.0577	04:13	744.841675
0.499387315	371.969971	1.9610	04:16	744.852661
0.449446438	334.769592	1.8705	04:18	744.848694
0.398771023	297.011322	1.7859	04:20	744.816711
0.348918623	259.875885	1.7025	04:22	744.803711
0.299239082	222.872589	1.6238	04:24	744.797729
0.249363158	185.725372	1.5426	04:26	744.798767
0.199605126	148.663849	1.4548	04:29	744.789734
0.139601128	103.971817	1.3458	04:32	744.777771
0.100013469	74.486710	1.2505	04:35	744.766785

Sample: campione Cagliari macinato
Operator:
Submitter:
File: C:\ASAP 2020 Plus\data\Chiappone\2022-1...\000-223.SMP

Started:	19/10/2022 11:35:41	Analysis adsorptive:	N2
Completed:	19/10/2022 16:31:25	Analysis bath temp.:	77,188 K
Report time:	20/10/2022 18:25:04	Thermal correction:	No
Sample mass:	0,3526 g	Ambient free space:	15,7578 cm³ Measured
Analysis free space:	45,2933 cm³	Equilibration interval:	10 s
Low pressure dose:	None	Sample density:	1,000 g/cm³
Automatic degas:	Yes		

Sample prep: Stage	Temperature (°C)	Ramp Rate (°C/min)	Time (min)
1	30	10	10

Isotherm Linear Plot

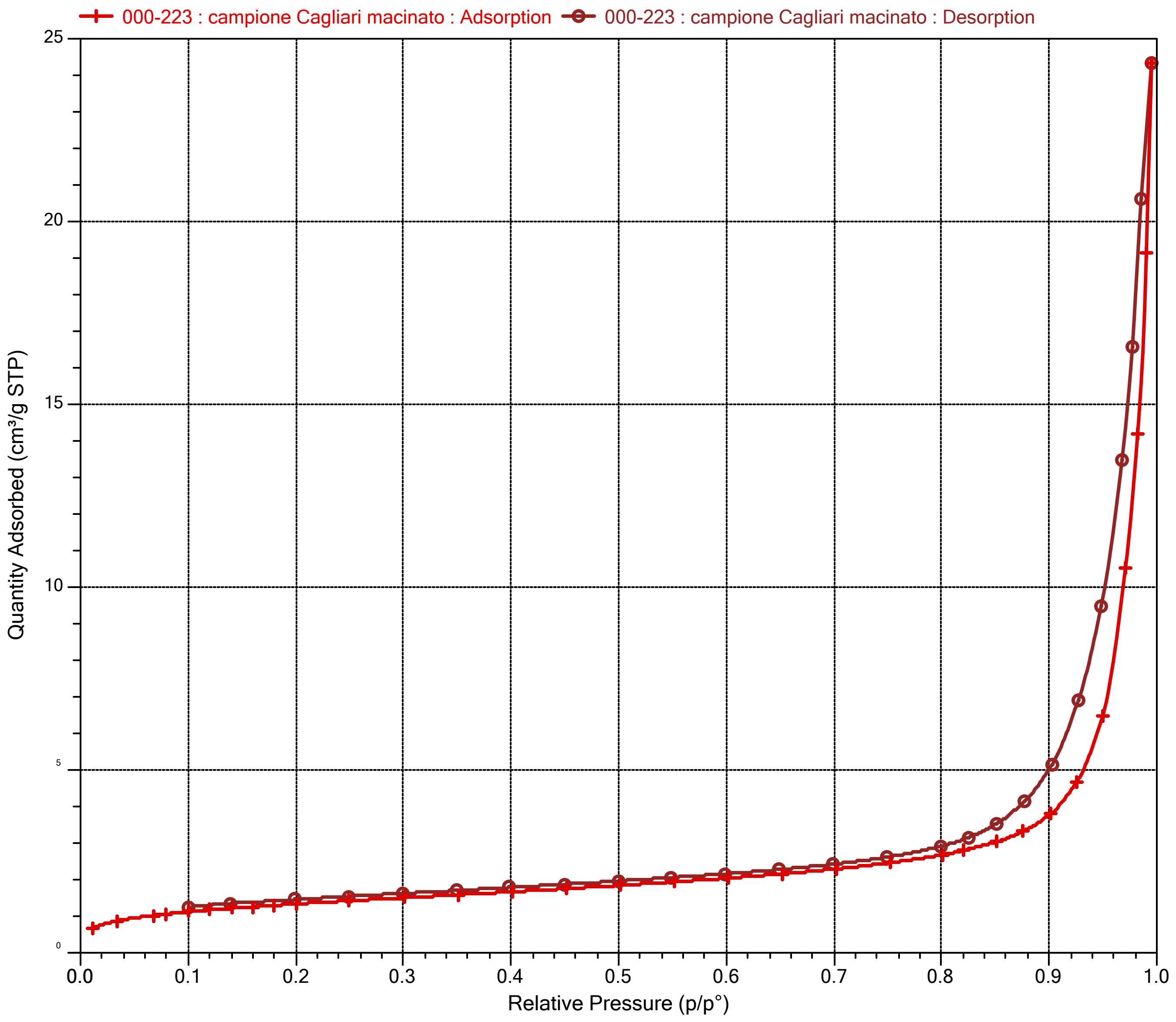

Sample: campione Cagliari macinato
Operator:
Submitter:
File: C:\ASAP 2020 Plus\data\Chiappone\2022-1...\000-223.SMP

Started:	19/10/2022 11:35:41	Analysis adsorptive:	N2
Completed:	19/10/2022 16:31:25	Analysis bath temp.:	77,188 K
Report time:	20/10/2022 18:25:04	Thermal correction:	No
Sample mass:	0,3526 g	Ambient free space:	15,7578 cm³ Measured
Analysis free space:	45,2933 cm³	Equilibration interval:	10 s
Low pressure dose:	None	Sample density:	1,000 g/cm³
Automatic degas:	Yes		

Sample prep: Stage	Temperature (°C)	Ramp Rate (°C/min)	Time (min)
1	30	10	10

Isotherm Log Plot

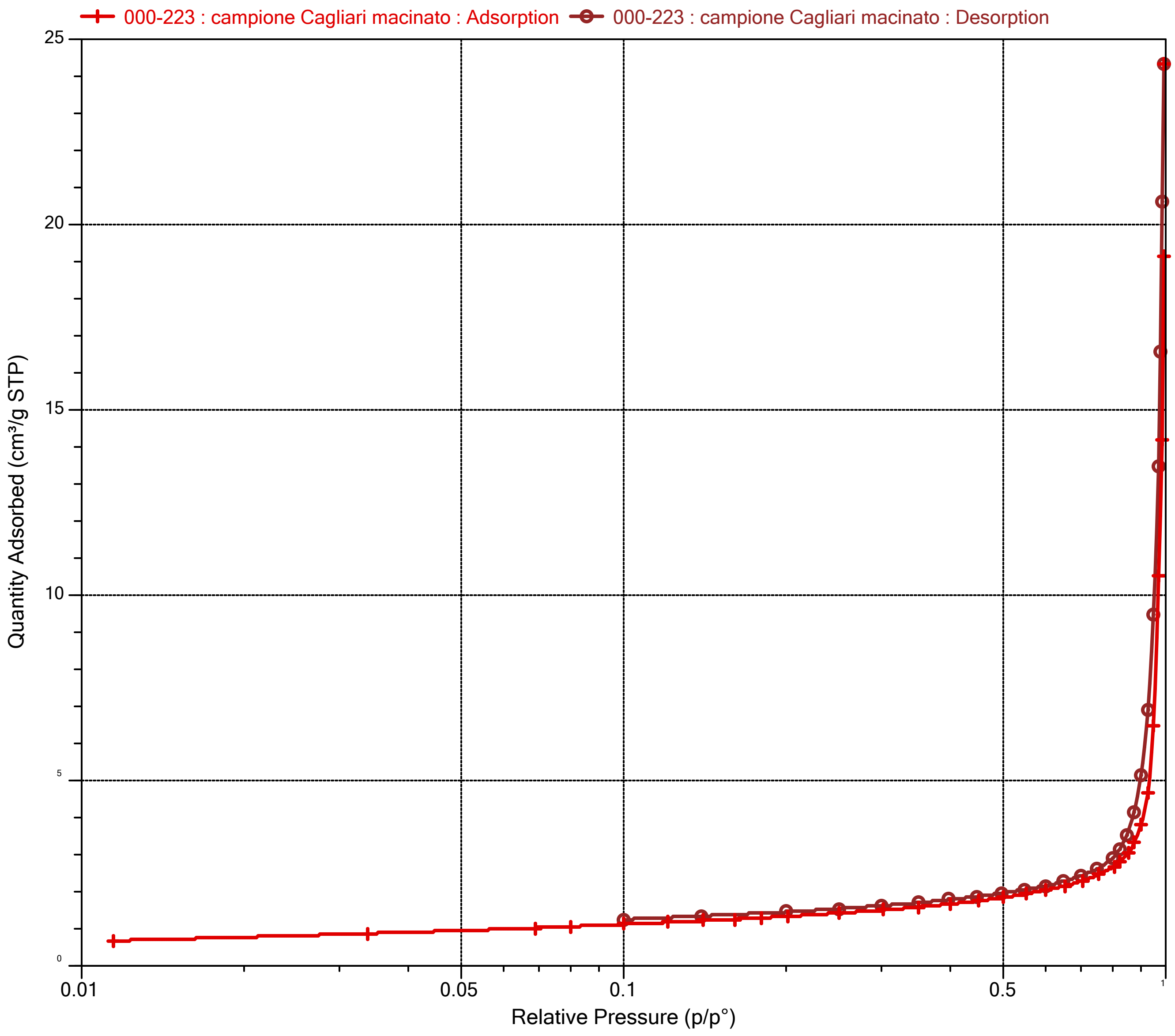

Sample: campione Cagliari macinato
Operator:
Submitter:
File: C:\ASAP 2020 Plus\data\Chiappone\2022-1...\000-223.SMP

Started:	19/10/2022 11:35:41	Analysis adsorptive:	N2
Completed:	19/10/2022 16:31:25	Analysis bath temp.:	77,188 K
Report time:	20/10/2022 18:25:04	Thermal correction:	No
Sample mass:	0,3526 g	Ambient free space:	15,7578 cm³ Measured
Analysis free space:	45,2933 cm³	Equilibration interval:	10 s
Low pressure dose:	None	Sample density:	1,000 g/cm³
Automatic degas:	Yes		

Sample prep: Stage	Temperature (°C)	Ramp Rate (°C/min)	Time (min)
1	30	10	10

Isotherm Linear Absolute Plot

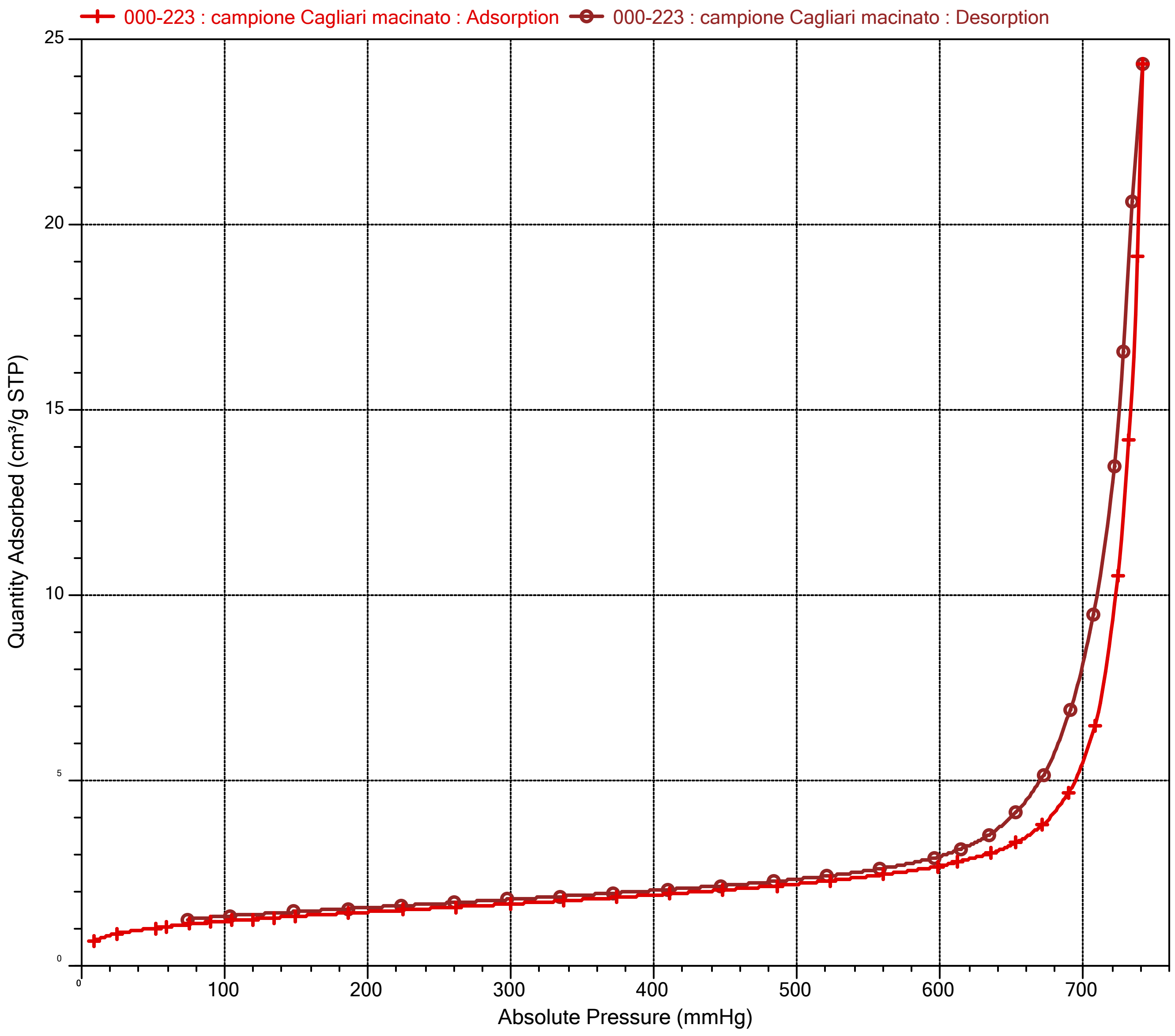

Sample: campione Cagliari macinato
Operator:
Submitter:
File: C:\ASAP 2020 Plus\data\Chiappone\2022-1...\000-223.SMP

Started:	19/10/2022 11:35:41	Analysis adsorptive:	N2
Completed:	19/10/2022 16:31:25	Analysis bath temp.:	77,188 K
Report time:	20/10/2022 18:25:04	Thermal correction:	No
Sample mass:	0,3526 g	Ambient free space:	15,7578 cm³ Measured
Analysis free space:	45,2933 cm³	Equilibration interval:	10 s
Low pressure dose:	None	Sample density:	1,000 g/cm³
Automatic degas:	Yes		

Sample prep: Stage	Temperature (°C)	Ramp Rate (°C/min)	Time (min)
1	30	10	10

Isotherm Log Absolute Plot

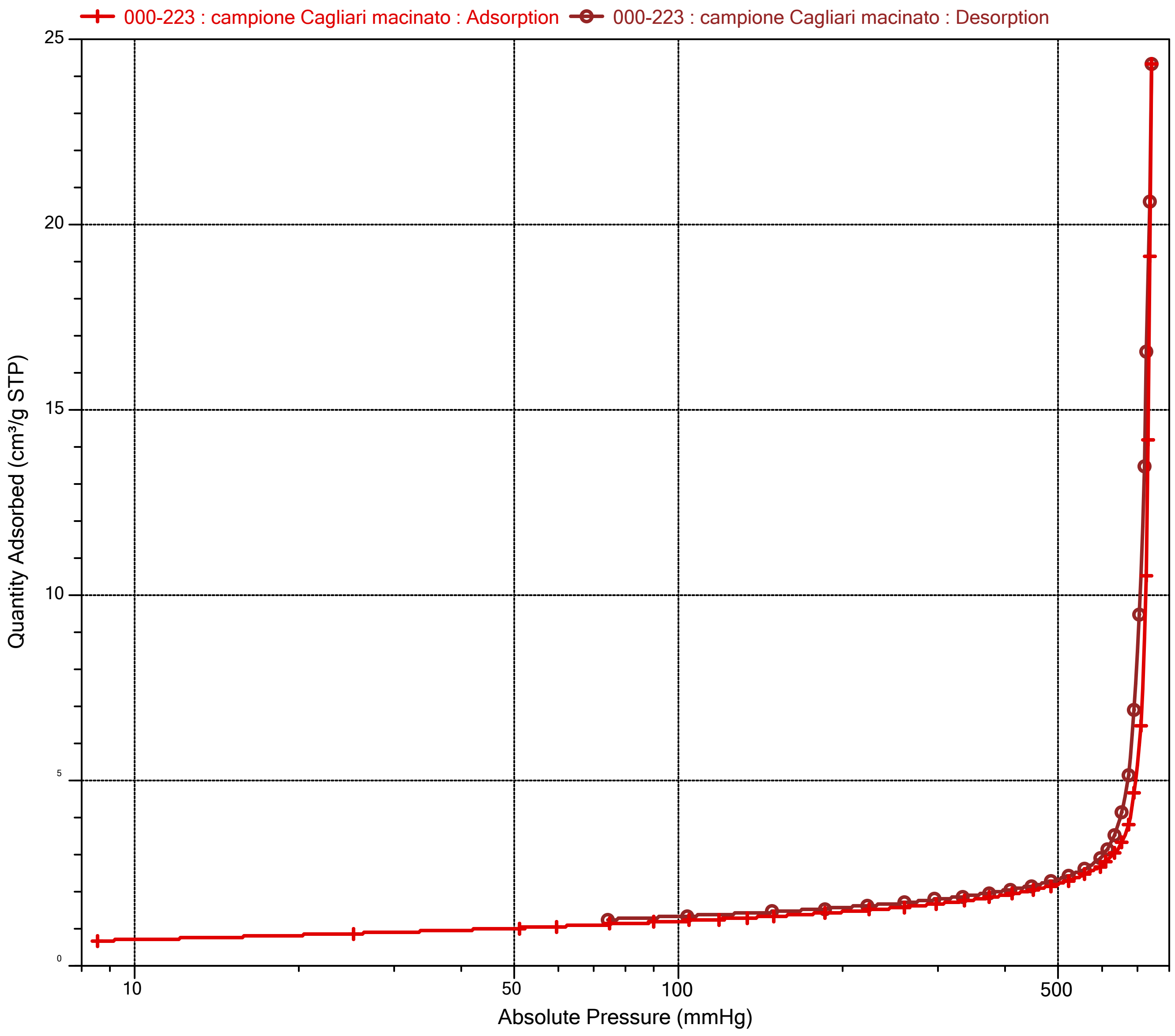

Sample: campione Cagliari macinato
Operator:
Submitter:
File: C:\ASAP 2020 Plus\data\Chiappone\2022-1...\000-223.SMP

Started:	19/10/2022 11:35:41	Analysis adsorptive:	N2
Completed:	19/10/2022 16:31:25	Analysis bath temp.:	77,188 K
Report time:	20/10/2022 18:25:04	Thermal correction:	No
Sample mass:	0,3526 g	Ambient free space:	15,7578 cm³ Measured
Analysis free space:	45,2933 cm³	Equilibration interval:	10 s
Low pressure dose:	None	Sample density:	1,000 g/cm³
Automatic degas:	Yes		

Sample prep: Stage	Temperature (°C)	Ramp Rate (°C/min)	Time (min)
1	30	10	10

Isotherm Pressure Composition

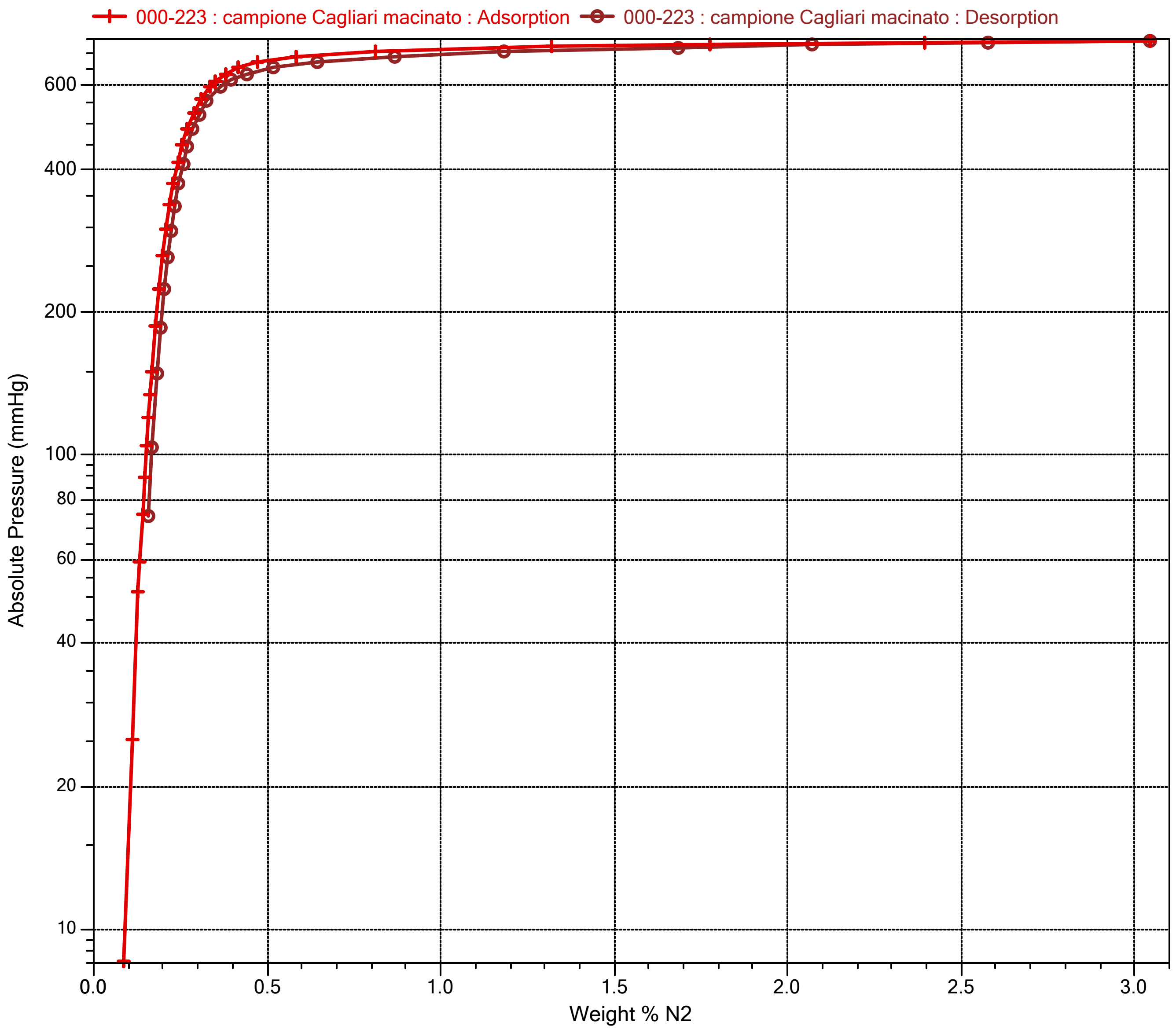

Sample: campione Cagliari macinato
Operator:
Submitter:
File: C:\ASAP 2020 Plus\data\Chiappone\2022-1...\000-223.SMP

Started:	19/10/2022 11:35:41	Analysis adsorptive:	N2
Completed:	19/10/2022 16:31:25	Analysis bath temp.:	77,188 K
Report time:	20/10/2022 18:25:04	Thermal correction:	No
Sample mass:	0,3526 g	Ambient free space:	15,7578 cm³ Measured
Analysis free space:	45,2933 cm³	Equilibration interval:	10 s
Low pressure dose:	None	Sample density:	1,000 g/cm³
Automatic degas:	Yes		

Sample prep: Stage	Temperature (°C)	Ramp Rate (°C/min)	Time (min)
1	30	10	10

BET Report

BET surface area: 4,8426 ± 0,0324 m²/g
Slope: 0,888625 ± 0,005942 g/cm³ STP
Y-intercept: 0,010185 ± 0,000920 g/cm³ STP
C: 88,248337
Qm: 1,1126 cm³/g STP
Correlation coefficient: 0,9998435
Molecular cross-sectional area: 0,1620 nm²

Relative Pressure (p/p°)	Quantity Adsorbed (cm³/g STP)	1/[Q(p°/p - 1)]
0.068677282	1.0232	0.072068
0.079940468	1.0618	0.081829
0.100049911	1.1201	0.099252
0.120504693	1.1715	0.116962
0.140480737	1.2151	0.134504
0.159878107	1.2582	0.151248
0.179888289	1.2963	0.169205
0.200381282	1.3365	0.187501
0.249708467	1.4231	0.233873

Sample: campione Cagliari macinato
Operator:
Submitter:
File: C:\ASAP 2020 Plus\data\Chiappone\2022-1...\000-223.SMP

Started:	19/10/2022 11:35:41	Analysis adsorptive:	N2
Completed:	19/10/2022 16:31:25	Analysis bath temp.:	77,188 K
Report time:	20/10/2022 18:25:04	Thermal correction:	No
Sample mass:	0,3526 g	Ambient free space:	15,7578 cm³ Measured
Analysis free space:	45,2933 cm³	Equilibration interval:	10 s
Low pressure dose:	None	Sample density:	1,000 g/cm³
Automatic degas:	Yes		

Sample prep: Stage	Temperature (°C)	Ramp Rate (°C/min)	Time (min)
1	30	10	10

BET Surface Area Plot

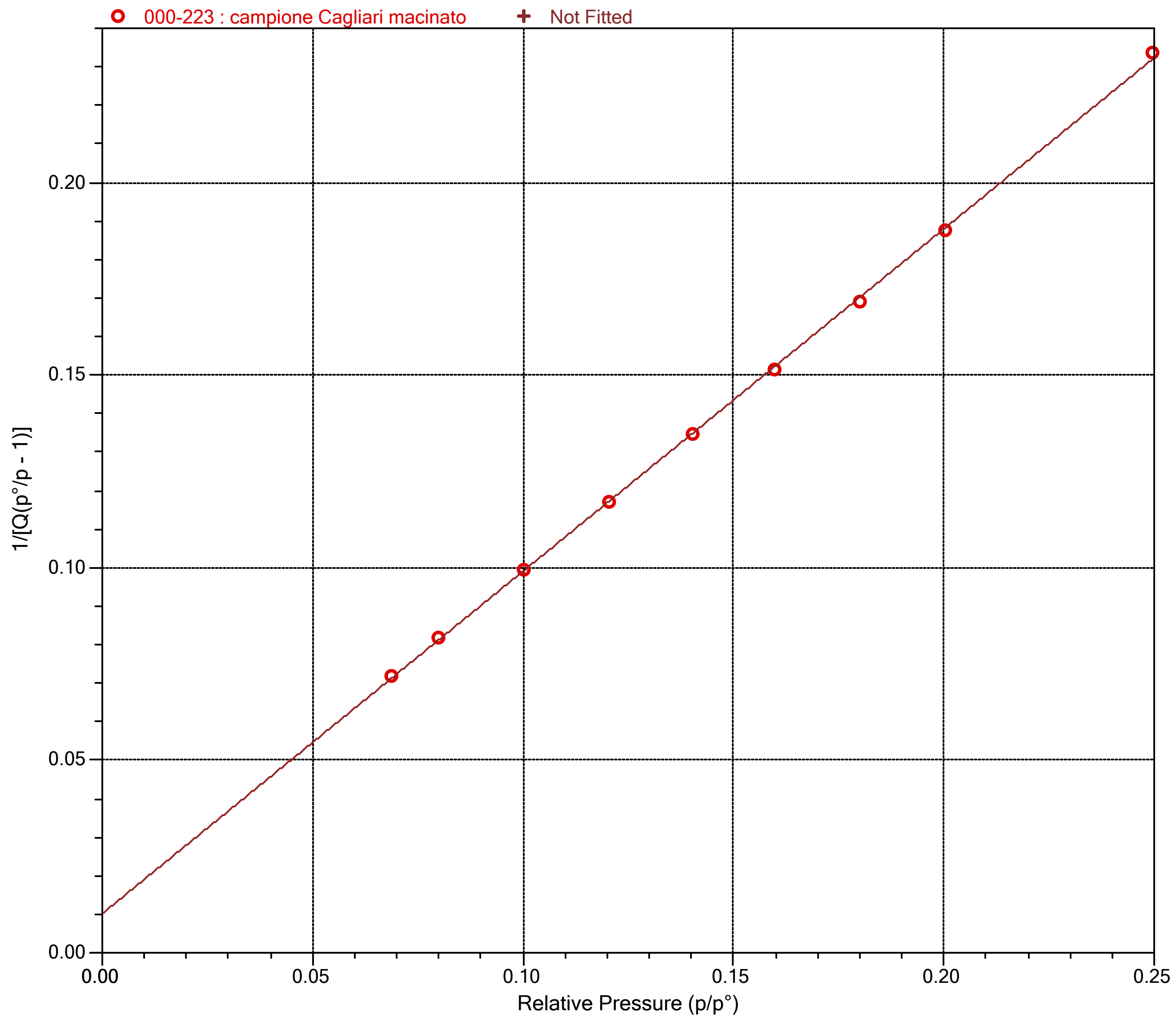

Sample: campione Cagliari macinato
Operator:
Submitter:
File: C:\ASAP 2020 Plus\data\Chiappone\2022-1...\000-223.SMP

Started: 19/10/2022 11:35:41
Completed: 19/10/2022 16:31:25
Report time: 20/10/2022 18:25:04
Sample mass: 0,3526 g
Analysis free space: 45,2933 cm³
Low pressure dose: None
Automatic degas: Yes

Analysis adsorptive: N2
Analysis bath temp.: 77,188 K
Thermal correction: No
Ambient free space: 15,7578 cm³ Measured
Equilibration interval: 10 s
Sample density: 1,000 g/cm³

Sample prep: Stage	Temperature (°C)	Ramp Rate (°C/min)	Time (min)
1	30	10	10

Langmuir Report

Langmuir surface area: 44,3943 ± 21,4265 m²/g
Slope: 0,098044 ± 0,047320 g/cm³ STP
Y-intercept: 95,913 ± 22,006 g/cm³ STP·mmHg
b: 0,001022 1/mmHg
Qm: 10,1995 cm³/g STP
Correlation coefficient: 0,353814
Molecular cross-sectional area: 0,1620 nm²

Pressure (mmHg)	Quantity Adsorbed (cm³/g STP)	p/Q (g/cm³ STP·mmHg)
8.555197	0.6789	12.601
25.212904	0.8733	28.870
51.249722	1.0232	50.086
59.651485	1.0618	56.179
74.653831	1.1201	66.649
89.912277	1.1715	76.753
104.813515	1.2151	86.256
119.280426	1.2582	94.801
134.200638	1.2963	103.523
149.487457	1.3365	111.850
186.282547	1.4231	130.903
224.212997	1.5032	149.156
261.594879	1.5874	164.793
299.235931	1.6693	179.259
336.567017	1.7547	191.812
373.881531	1.8446	202.688
411.206940	1.9417	211.782
448.366119	2.0440	219.358
485.951324	2.1607	224.908
523.206482	2.2953	227.945
560.521118	2.4638	227.498
597.780090	2.6811	222.960
612.285645	2.8020	218.517
634.834595	3.0346	209.200
653.130798	3.3207	196.687
671.631470	3.7930	177.069
689.895996	4.6745	147.586
707.694458	6.4900	109.045
724.288696	10.5363	68.742
731.840942	14.2059	51.517
737.964417	19.1602	38.515
741.368286	24.3458	30.452

Sample: campione Cagliari macinato
Operator:
Submitter:
File: C:\ASAP 2020 Plus\data\Chiappone\2022-1...\000-223.SMP

Started:	19/10/2022 11:35:41	Analysis adsorptive:	N2
Completed:	19/10/2022 16:31:25	Analysis bath temp.:	77,188 K
Report time:	20/10/2022 18:25:04	Thermal correction:	No
Sample mass:	0,3526 g	Ambient free space:	15,7578 cm³ Measured
Analysis free space:	45,2933 cm³	Equilibration interval:	10 s
Low pressure dose:	None	Sample density:	1,000 g/cm³
Automatic degas:	Yes		

Sample prep: Stage	Temperature (°C)	Ramp Rate (°C/min)	Time (min)
1	30	10	10

Langmuir Surface Area Plot

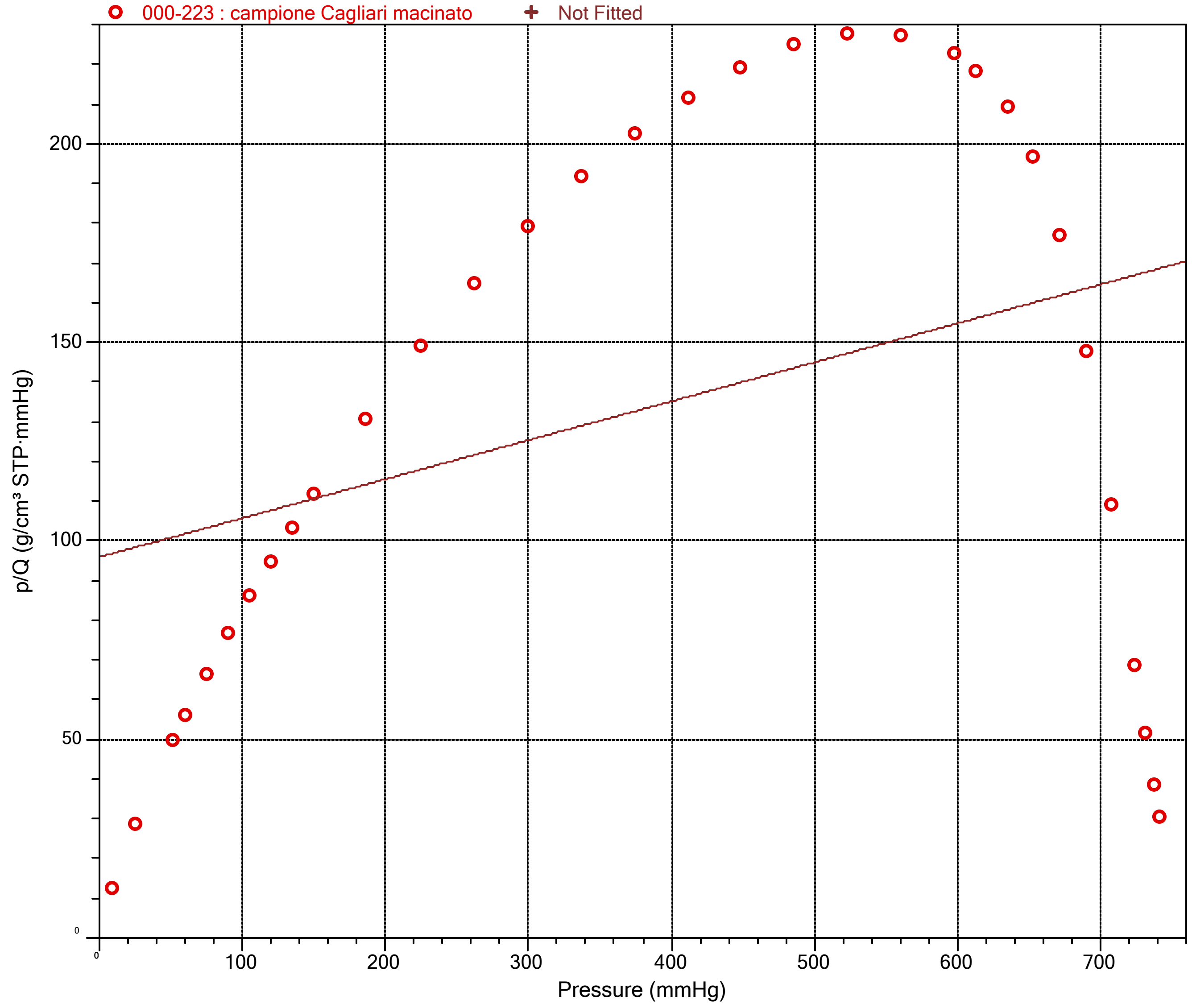

Sample: campione Cagliari macinato
Operator:
Submitter:
File: C:\ASAP 2020 Plus\data\Chiappone\2022-1...\000-223.SMP

Started:	19/10/2022 11:35:41	Analysis adsorptive:	N2
Completed:	19/10/2022 16:31:25	Analysis bath temp.:	77,188 K
Report time:	20/10/2022 18:25:04	Thermal correction:	No
Sample mass:	0,3526 g	Ambient free space:	15,7578 cm³ Measured
Analysis free space:	45,2933 cm³	Equilibration interval:	10 s
Low pressure dose:	None	Sample density:	1,000 g/cm³
Automatic degas:	Yes		

Sample prep: Stage	Temperature (°C)	Ramp Rate (°C/min)	Time (min)
1	30	10	10

t-Plot Report

Micropore volume:	-0,000020 cm³/g
Micropore area:	*
External surface area:	4,7682 m²/g
Slope:	3,082619 ± 0,062266 cm³/g·nm STP
Y-intercept:	-0,013200 ± 0,025317 cm³/g STP
Correlation coefficient:	0,998778
Surface area correction factor:	1,000
Density conversion factor:	0,0015468
Total surface area (BET):	4,8426 m²/g
Thickness range:	0,35000 to 0,50000 nm
Thickness equation:	Harkins and Jura

Thickness Curve

t = [13.99 / (0.034 - log(p/p°))] ^ 0.5

t-Plot Report - Data

Relative Pressure (p/p°)	Statistical Thickness (nm)	Quantity Adsorbed (cm³/g STP)	Fitted
0.068677282	0.34184	1.0232	
0.079940468	0.35167	1.0618	*
0.100049911	0.36787	1.1201	*
0.120504693	0.38315	1.1715	*
0.140480737	0.39728	1.2151	*
0.159878107	0.41050	1.2582	*
0.179888289	0.42378	1.2963	*
0.200381282	0.43713	1.3365	*
0.249708467	0.46880	1.4231	*
0.300568445	0.50159	1.5032	
0.350704638	0.53485	1.5874	
0.401172050	0.56995	1.6693	
0.451228599	0.60708	1.7547	
0.501302383	0.64729	1.8446	
0.551384683	0.69153	1.9417	
0.601216792	0.74074	2.0440	
0.651562704	0.79736	2.1607	

* The micropore area is not reported because either the micropore volume is negative or the calculated external surface area is larger than the total surface area.

Sample: campione Cagliari macinato
Operator:
Submitter:
File: C:\ASAP 2020 Plus\data\Chiappone\2022-1...\000-223.SMP

Started:	19/10/2022 11:35:41	Analysis adsorptive:	N2
Completed:	19/10/2022 16:31:25	Analysis bath temp.:	77,188 K
Report time:	20/10/2022 18:25:04	Thermal correction:	No
Sample mass:	0,3526 g	Ambient free space:	15,7578 cm³ Measured
Analysis free space:	45,2933 cm³	Equilibration interval:	10 s
Low pressure dose:	None	Sample density:	1,000 g/cm³
Automatic degas:	Yes		

Sample prep: Stage	Temperature (°C)	Ramp Rate (°C/min)	Time (min)
1	30	10	10

t-Plot

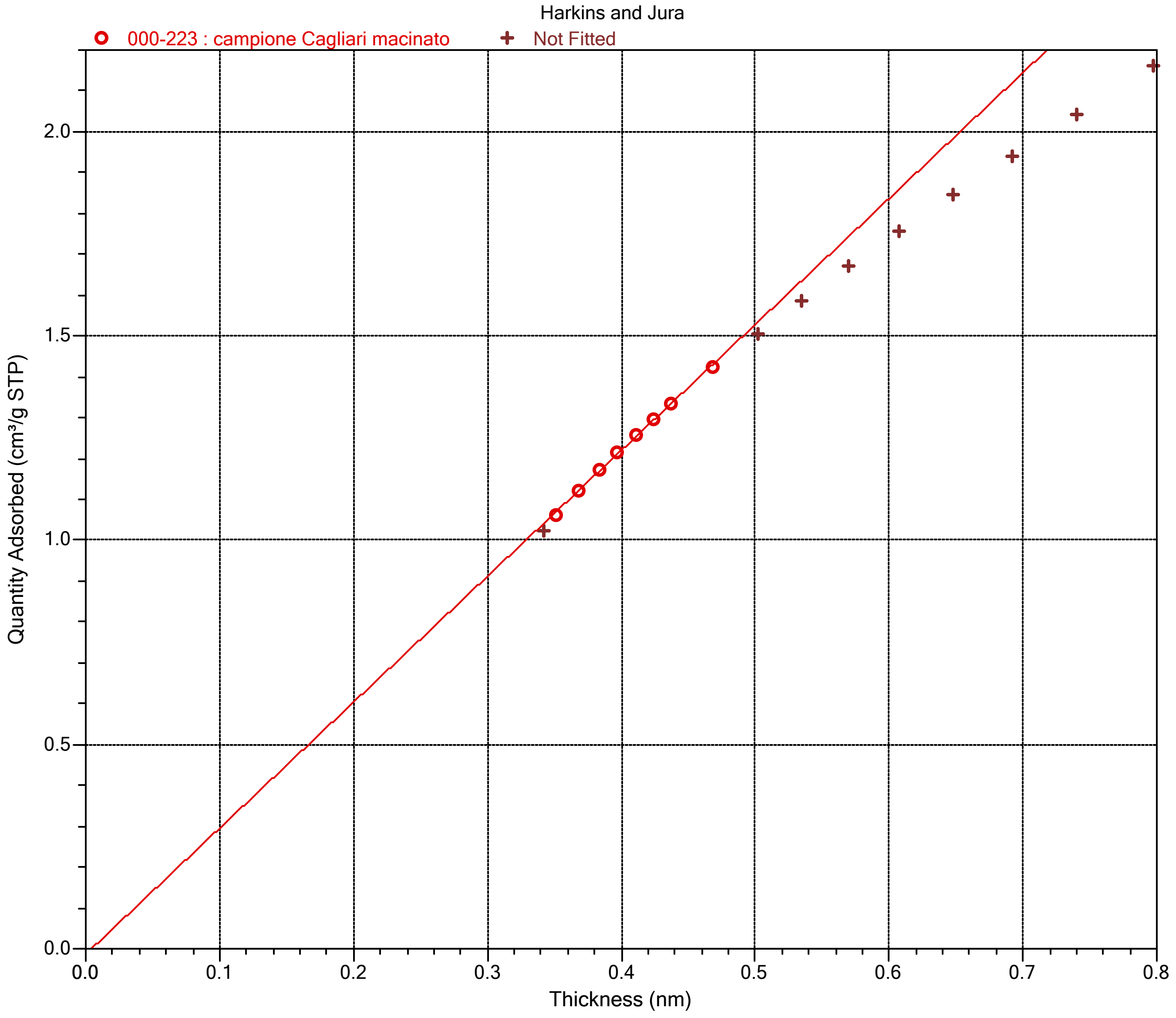

Sample: campione Cagliari macinato
Operator:
Submitter:
File: C:\ASAP 2020 Plus\data\Chiappone\2022-1...\000-223.SMP

Started:	19/10/2022 11:35:41	Analysis adsorptive:	N2
Completed:	19/10/2022 16:31:25	Analysis bath temp.:	77,188 K
Report time:	20/10/2022 18:25:04	Thermal correction:	No
Sample mass:	0,3526 g	Ambient free space:	15,7578 cm³ Measured
Analysis free space:	45,2933 cm³	Equilibration interval:	10 s
Low pressure dose:	None	Sample density:	1,000 g/cm³
Automatic degas:	Yes		

Sample prep: Stage	Temperature (°C)	Ramp Rate (°C/min)	Time (min)
1	30	10	10

BJH Adsorption Pore Distribution Report

Faas Correction
Harkins and Jura
$t = [13.99 / (0.034 - \log(p/p°))] ^ 0.5$

Width range: 1,7000 to 300,0000 nm
Adsorbate property factor: 0,95300 nm
Density conversion factor: 0,0015468
Fraction of pores open at both ends: 0,00

Pore Width Range (nm)	Average Width (nm)	Incremental Pore Volume (cm³/g)	Cumulative Pore Volume (cm³/g)	Incremental Pore Area (m²/g)	Cumulative Pore Area (m²/g)
364.5 - 195.0	232.2	0.008292	0.008292	0.143	0.143
195.0 - 107.2	127.1	0.008111	0.016402	0.255	0.398
107.2 - 69.4	80.3	0.006161	0.022563	0.307	0.705
69.4 - 39.7	46.7	0.007068	0.029631	0.606	1.311
39.7 - 27.4	31.3	0.003202	0.032833	0.410	1.720
27.4 - 20.9	23.2	0.001510	0.034343	0.260	1.980
20.9 - 16.9	18.4	0.000748	0.035091	0.162	2.143
16.9 - 14.2	15.3	0.000402	0.035493	0.105	2.248
14.2 - 11.8	12.8	0.000280	0.035773	0.088	2.336
11.8 - 10.7	11.2	0.000135	0.035908	0.048	2.384
10.7 - 8.6	9.4	0.000188	0.036096	0.080	2.465
8.6 - 7.1	7.7	0.000132	0.036228	0.069	2.533
7.1 - 6.0	6.5	0.000091	0.036319	0.056	2.590
6.0 - 5.2	5.6	0.000078	0.036398	0.056	2.646
5.2 - 4.6	4.9	0.000070	0.036467	0.057	2.703
4.6 - 4.1	4.3	0.000077	0.036545	0.072	2.775
4.1 - 3.6	3.8	0.000074	0.036619	0.078	2.853
3.6 - 3.2	3.4	0.000075	0.036693	0.088	2.942
3.2 - 2.9	3.0	0.000073	0.036766	0.096	3.037
2.9 - 2.6	2.7	0.000092	0.036858	0.135	3.172
2.6 - 2.3	2.4	0.000075	0.036933	0.124	3.296
2.3 - 2.1	2.2	0.000108	0.037041	0.200	3.496
2.1 - 2.0	2.0	0.000060	0.037102	0.120	3.616
2.0 - 1.9	1.9	0.000050	0.037151	0.104	3.720
1.9 - 1.8	1.8	0.000073	0.037224	0.162	3.882
1.8 - 1.7	1.7	0.000064	0.037288	0.149	4.030

Sample: campione Cagliari macinato
Operator:
Submitter:
File: C:\ASAP 2020 Plus\data\Chiappone\2022-1...\000-223.SMP

Started:	19/10/2022 11:35:41	Analysis adsorptive:	N2
Completed:	19/10/2022 16:31:25	Analysis bath temp.:	77,188 K
Report time:	20/10/2022 18:25:04	Thermal correction:	No
Sample mass:	0,3526 g	Ambient free space:	15,7578 cm³ Measured
Analysis free space:	45,2933 cm³	Equilibration interval:	10 s
Low pressure dose:	None	Sample density:	1,000 g/cm³
Automatic degas:	Yes		

Sample prep: Stage	Temperature (°C)	Ramp Rate (°C/min)	Time (min)
1	30	10	10

BJH Adsorption Cumulative Pore Volume (Larger)

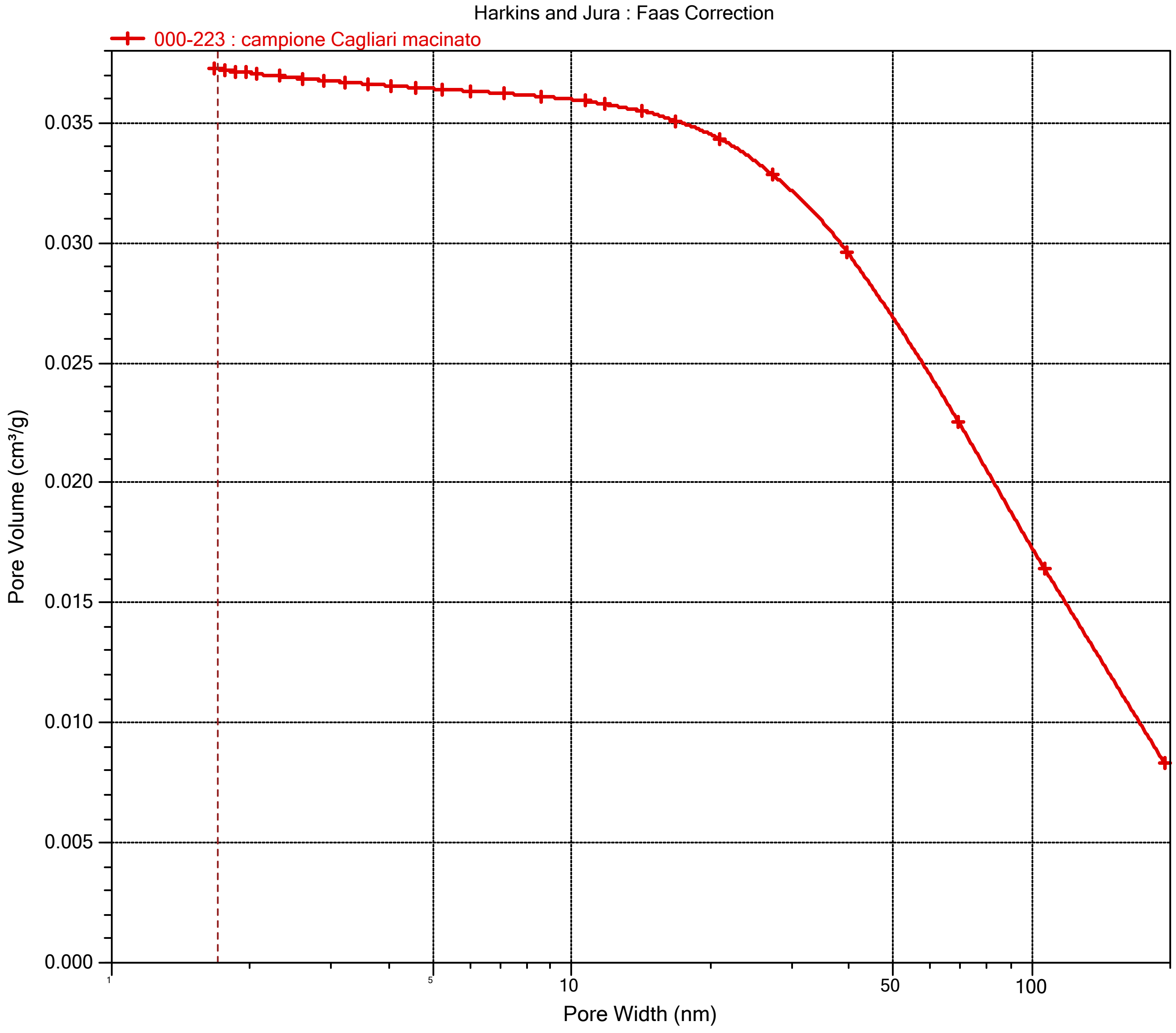

Sample: campione Cagliari macinato
Operator:
Submitter:
File: C:\ASAP 2020 Plus\data\Chiappone\2022-1...\000-223.SMP

Started:	19/10/2022 11:35:41	Analysis adsorptive:	N2
Completed:	19/10/2022 16:31:25	Analysis bath temp.:	77,188 K
Report time:	20/10/2022 18:25:04	Thermal correction:	No
Sample mass:	0,3526 g	Ambient free space:	15,7578 cm³ Measured
Analysis free space:	45,2933 cm³	Equilibration interval:	10 s
Low pressure dose:	None	Sample density:	1,000 g/cm³
Automatic degas:	Yes		

Sample prep: Stage	Temperature (°C)	Ramp Rate (°C/min)	Time (min)
1	30	10	10

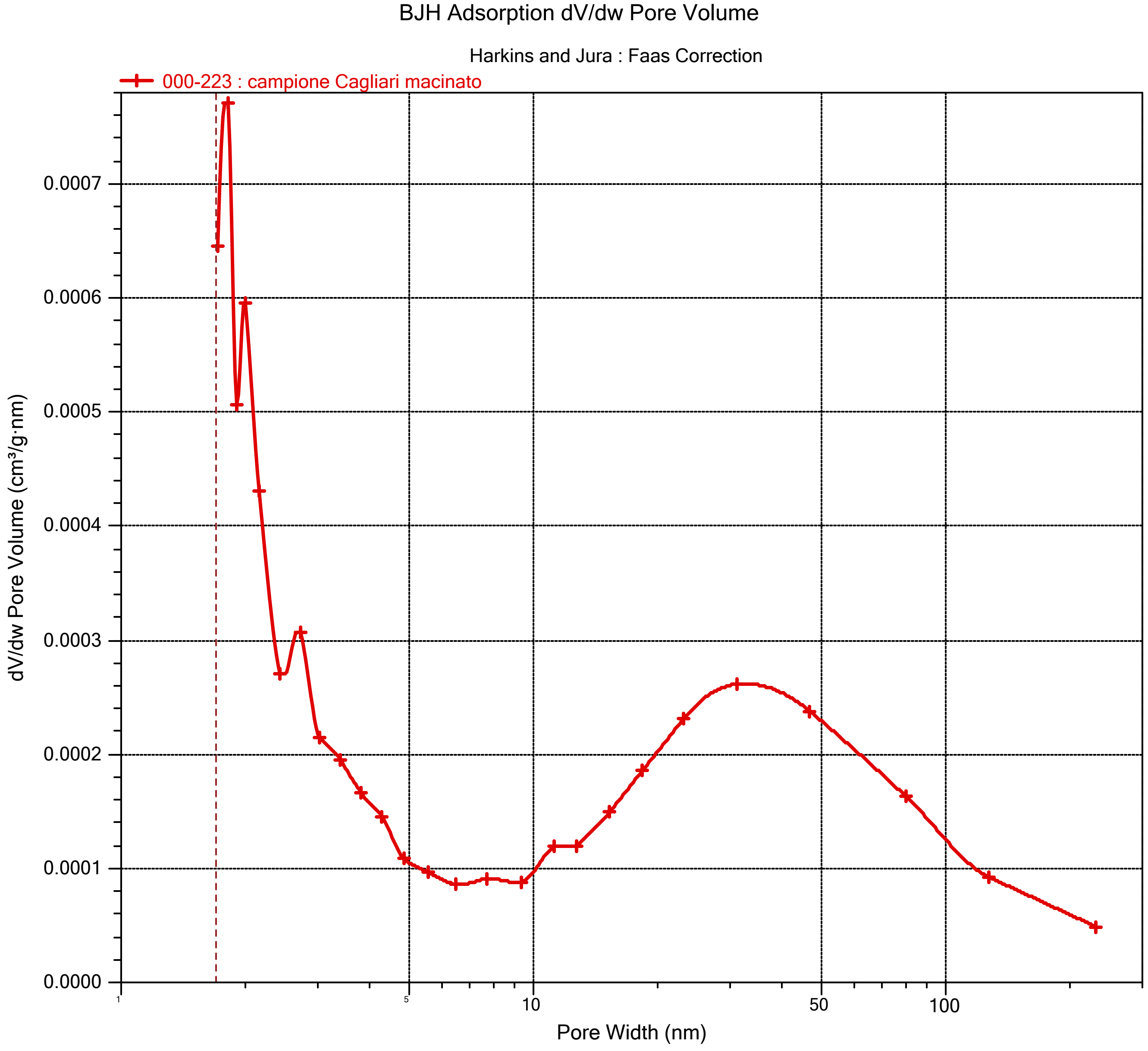

Sample: campione Cagliari macinato
Operator:
Submitter:
File: C:\ASAP 2020 Plus\data\Chiappone\2022-1...\000-223.SMP

Started:	19/10/2022 11:35:41	Analysis adsorptive:	N2
Completed:	19/10/2022 16:31:25	Analysis bath temp.:	77,188 K
Report time:	20/10/2022 18:25:04	Thermal correction:	No
Sample mass:	0,3526 g	Ambient free space:	15,7578 cm³ Measured
Analysis free space:	45,2933 cm³	Equilibration interval:	10 s
Low pressure dose:	None	Sample density:	1,000 g/cm³
Automatic degas:	Yes		

Sample prep: Stage	Temperature (°C)	Ramp Rate (°C/min)	Time (min)
1	30	10	10

BJH Desorption Pore Distribution Report

Faas Correction
Harkins and Jura
$t = [13.99 / (0.034 - \log(p/p°))] ^ 0.5$

Width range: 1,7000 to 300,0000 nm
Adsorbate property factor: 0,95300 nm
Density conversion factor: 0,0015468
Fraction of pores open at both ends: 0,00

Pore Width Range (nm)	Average Width (nm)	Incremental Pore Volume (cm³/g)	Cumulative Pore Volume (cm³/g)	Incremental Pore Area (m²/g)	Cumulative Pore Area (m²/g)
364.4 - 137.6	165.1	0.006052	0.006052	0.147	0.147
137.6 - 85.7	99.9	0.006721	0.012773	0.269	0.416
85.7 - 62.4	70.3	0.005201	0.017974	0.296	0.712
62.4 - 39.3	45.7	0.007097	0.025071	0.622	1.333
39.3 - 28.4	32.0	0.004546	0.029617	0.568	1.901
28.4 - 21.2	23.7	0.003208	0.032825	0.542	2.443
21.2 - 16.9	18.6	0.001799	0.034625	0.388	2.831
16.9 - 14.1	15.2	0.001016	0.035640	0.266	3.097
14.1 - 12.1	12.9	0.000556	0.036196	0.172	3.269
12.1 - 10.6	11.2	0.000305	0.036501	0.109	3.378
10.6 - 8.5	9.3	0.000247	0.036748	0.107	3.484
8.5 - 7.0	7.6	0.000075	0.036823	0.040	3.524
7.0 - 6.0	6.4	0.000024	0.036847	0.015	3.539
6.0 - 5.2	5.5	0.000003	0.036849	0.002	3.541
5.2 - 4.0	4.3	0.000005	0.036854	0.005	3.545
4.0 - 3.6	3.8	0.000012	0.036866	0.012	3.558
3.6 - 3.2	3.4	0.000009	0.036875	0.011	3.568
3.2 - 2.9	3.0	0.000027	0.036902	0.036	3.604
2.9 - 2.6	2.7	0.000022	0.036924	0.032	3.636
2.6 - 2.3	2.4	0.000038	0.036961	0.062	3.698
2.3 - 2.1	2.2	0.000066	0.037027	0.122	3.820
2.1 - 1.8	1.9	0.000073	0.037100	0.156	3.975

Sample: campione Cagliari macinato
Operator:
Submitter:
File: C:\ASAP 2020 Plus\data\Chiappone\2022-1...\000-223.SMP

Started:	19/10/2022 11:35:41	Analysis adsorptive:	N2
Completed:	19/10/2022 16:31:25	Analysis bath temp.:	77,188 K
Report time:	20/10/2022 18:25:04	Thermal correction:	No
Sample mass:	0,3526 g	Ambient free space:	15,7578 cm³ Measured
Analysis free space:	45,2933 cm³	Equilibration interval:	10 s
Low pressure dose:	None	Sample density:	1,000 g/cm³
Automatic degas:	Yes		

Sample prep: Stage	Temperature (°C)	Ramp Rate (°C/min)	Time (min)
1	30	10	10

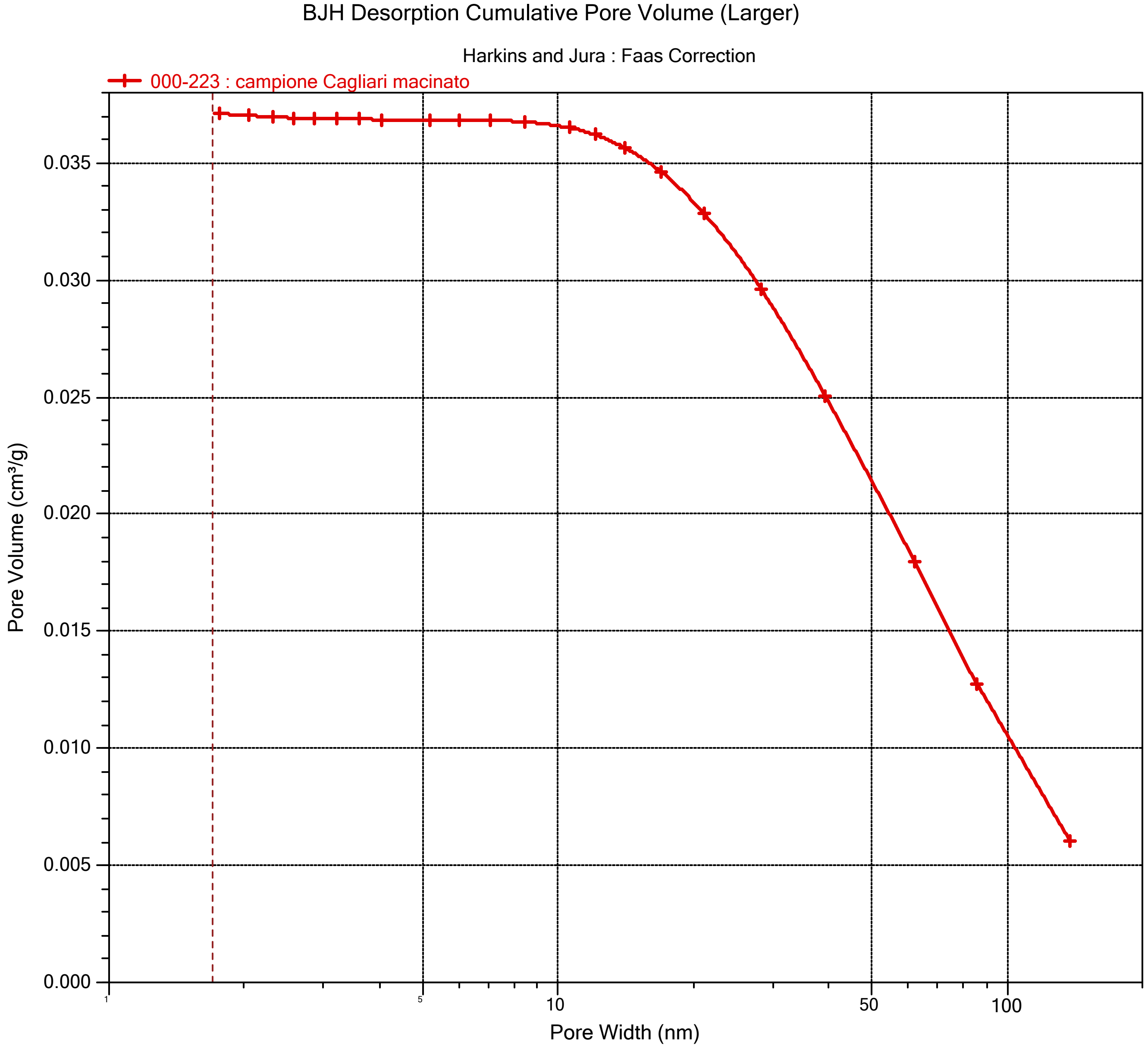

Sample: campione Cagliari macinato
Operator:
Submitter:
File: C:\ASAP 2020 Plus\data\Chiappone\2022-1...\000-223.SMP

Started:	19/10/2022 11:35:41	Analysis adsorptive:	N2
Completed:	19/10/2022 16:31:25	Analysis bath temp.:	77,188 K
Report time:	20/10/2022 18:25:04	Thermal correction:	No
Sample mass:	0,3526 g	Ambient free space:	15,7578 cm³ Measured
Analysis free space:	45,2933 cm³	Equilibration interval:	10 s
Low pressure dose:	None	Sample density:	1,000 g/cm³
Automatic degas:	Yes		

Sample prep: Stage	Temperature (°C)	Ramp Rate (°C/min)	Time (min)
1	30	10	10

BJH Desorption dV/dw Pore Volume

Harkins and Jura : Faas Correction

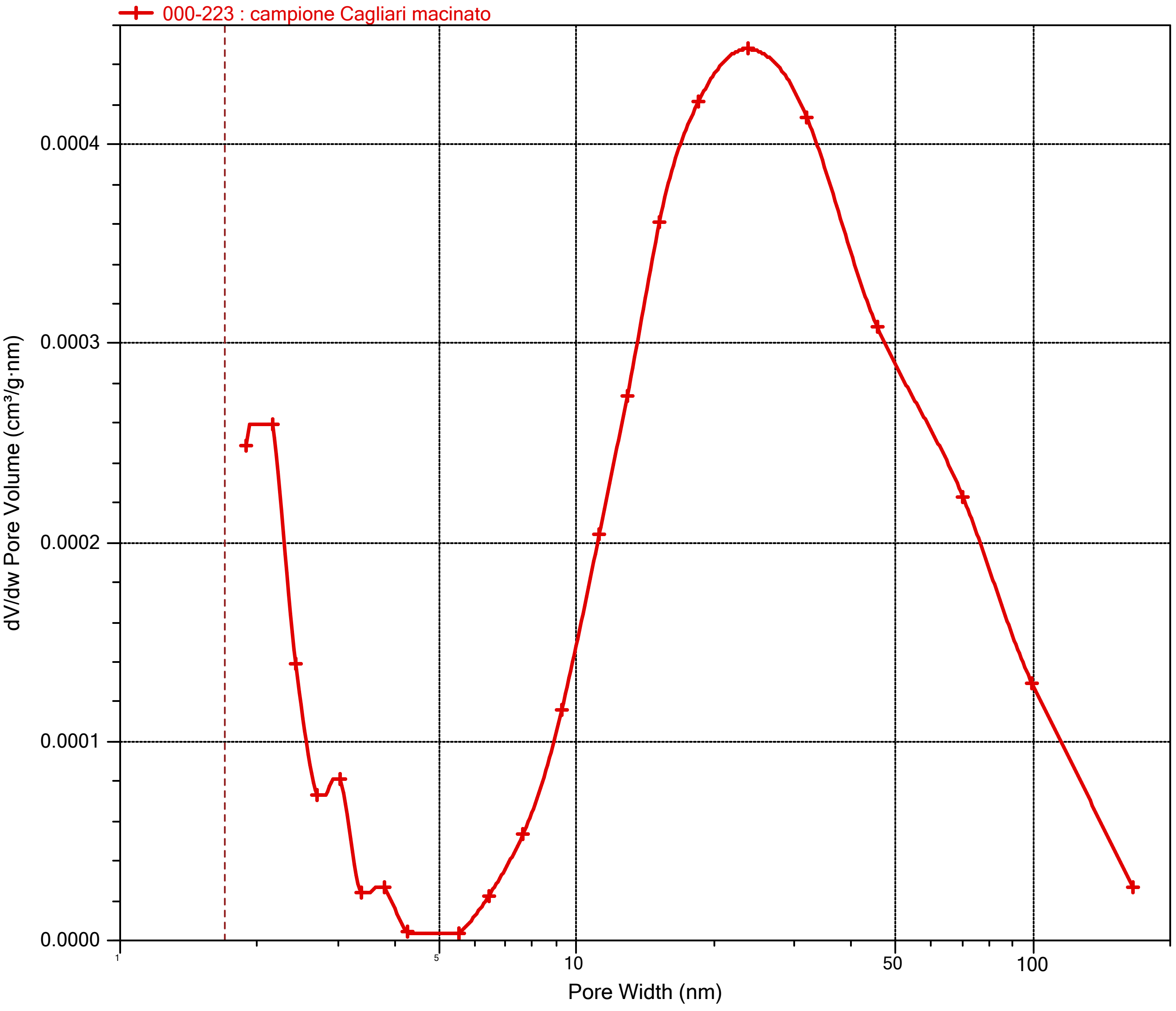

Sample: campione Cagliari macinato
Operator:
Submitter:
File: C:\ASAP 2020 Plus\data\Chiappone\2022-1...\000-223.SMP

Started:	19/10/2022 11:35:41	Analysis adsorptive:	N2
Completed:	19/10/2022 16:31:25	Analysis bath temp.:	77,188 K
Report time:	20/10/2022 18:25:04	Thermal correction:	No
Sample mass:	0,3526 g	Ambient free space:	15,7578 cm³ Measured
Analysis free space:	45,2933 cm³	Equilibration interval:	10 s
Low pressure dose:	None	Sample density:	1,000 g/cm³
Automatic degas:	Yes		

Sample prep: Stage	Temperature (°C)	Ramp Rate (°C/min)	Time (min)
1	30	10	10

DFT Pore Size Reports

Primary Data

4070- Unable to load deconvolution model Invalid.

Sample: campione Cagliari macinato
Operator:
Submitter:
File: C:\ASAP 2020 Plus\data\Chiappone\2022-1...\000-223.SMP

Started:	19/10/2022 11:35:41	Analysis adsorptive:	N2
Completed:	19/10/2022 16:31:25	Analysis bath temp.:	77,188 K
Report time:	20/10/2022 18:25:04	Thermal correction:	No
Sample mass:	0,3526 g	Ambient free space:	15,7578 cm³ Measured
Analysis free space:	45,2933 cm³	Equilibration interval:	10 s
Low pressure dose:	None	Sample density:	1,000 g/cm³
Automatic degas:	Yes		

Sample prep: Stage	Temperature (°C)	Ramp Rate (°C/min)	Time (min)
1	30	10	10

DFT Surface Energy Reports

Primary Data

4070- Unable to load deconvolution model Invalid.

micromeritics®

Sample: resine Cagliari pre-macinazione
Operator:
Submitter:
File: C:\ASAP 2020 Plus\data\Chiappone\2022-1...\000-225.SMP

Started:	24/10/2022 09:45:14	Analysis adsorptive:	N2
Completed:	24/10/2022 18:12:00	Analysis bath temp.:	77,159 K
Report time:	24/10/2022 20:01:54	Thermal correction:	No
Sample mass:	0,4650 g	Ambient free space:	27,2106 cm³ Measured
Analysis free space:	81,8113 cm³	Equilibration interval:	10 s
Low pressure dose:	None	Sample density:	1,000 g/cm³
Automatic degas:	Yes		

Sample prep: Stage	Temperature (°C)	Ramp Rate (°C/min)	Time (min)
1	30	10	10

Summary Report

Surface Area

Single point surface area at p/p° = 0,249877845: 27,5781 m²/g

BET Surface Area: 28,6015 m²/g

Langmuir Surface Area: 535,7181 m²/g

t-Plot Micropore Area: 1,2312 m²/g

t-Plot external surface area: 27,3703 m²/g

BJH Adsorption cumulative surface area of pores between 1,7000 nm and 300,0000 nm width: 24,8568 m²/g

BJH Desorption cumulative surface area of pores between 1,7000 nm and 300,0000 nm width: 30,6124 m²/g

D-H Adsorption cumulative surface area of pores between 1,7000 nm and 300,0000 nm width: 24,5523 m²/g

D-H Desorption cumulative surface area of pores between 1,7000 nm and 300,0000 nm width: 29,8710 m²/g

Pore Volume

Single point adsorption total pore volume of pores less than 40,3122 nm width at p/p° = 0,950000000: 0,078042 cm³/g

Single point desorption total pore volume of pores less than 40,3122 nm width at p/p° = 0,950000000: 0,184959 cm³/g

t-Plot micropore volume: 0,000298 cm³/g

BJH Adsorption cumulative volume of pores between 1,7000 nm and 300,0000 nm width: 0,215483 cm³/g

BJH Desorption cumulative volume of pores between 1,7000 nm and 300,0000 nm width: 0,217399 cm³/g

D-H Adsorption cumulative volume of pores between 1,7000 nm and 300,0000 nm width: 0,214571 cm³/g

D-H Desorption cumulative volume of pores

Sample: resine Cagliari pre-macinazione
Operator:
Submitter:
File: C:\ASAP 2020 Plus\data\Chiappone\2022-1...\000-225.SMP

Started:	24/10/2022 09:45:14	Analysis adsorptive:	N2
Completed:	24/10/2022 18:12:00	Analysis bath temp.:	77,159 K
Report time:	24/10/2022 20:01:54	Thermal correction:	No
Sample mass:	0,4650 g	Ambient free space:	27,2106 cm³ Measured
Analysis free space:	81,8113 cm³	Equilibration interval:	10 s
Low pressure dose:	None	Sample density:	1,000 g/cm³
Automatic degas:	Yes		

Sample prep: Stage	Temperature (°C)	Ramp Rate (°C/min)	Time (min)
1	30	10	10

Pore Volume

between 1,7000 nm and 300,0000 nm width: 0,219121 cm³/g

Pore Size

Adsorption average pore diameter (4V/A by BET): 10,9144 nm

Desorption average pore diameter (4V/A by BET): 25,8671 nm

BJH Adsorption average pore width (4V/A): 34,6760 nm

BJH Desorption average pore width (4V/A): 28,4066 nm

D-H Adsorption average pore width (4V/A): 34,9574 nm

D-H Desorption average pore width (4V/A): 29,3423 nm

Freundlich

Qm·C: 0,3865 ± 0,0996 cm³/g STP

m: 1,4920 ± 0,2311

Temkin

q·alpha/Qm: 0,036607 ± 0,010925 kJ/mol·(cm³/g STP)

A: 0,0203 ± 0,0419 mmHg

Nanoparticle Size:

Average Particle Size 209,7791 nm

Horvath-Kawazoe

Maximum pore volume at p/p° = 0,179942753: 0,011928 cm³/g

Median pore width: 0,7761 nm

MP-Method

Cumulative surface area of pores between
1,89666 nm and 1,89666 nm hydraulic radius: 0,0000 m²/g

Cumulative pore volume of pores between

Sample: resine Cagliari pre-macinazione
Operator:
Submitter:
File: C:\ASAP 2020 Plus\data\Chiappone\2022-1...\000-225.SMP

Started:	24/10/2022 09:45:14	Analysis adsorptive:	N2
Completed:	24/10/2022 18:12:00	Analysis bath temp.:	77,159 K
Report time:	24/10/2022 20:01:54	Thermal correction:	No
Sample mass:	0,4650 g	Ambient free space:	27,2106 cm³ Measured
Analysis free space:	81,8113 cm³	Equilibration interval:	10 s
Low pressure dose:	None	Sample density:	1,000 g/cm³
Automatic degas:	Yes		

Sample prep: Stage	Temperature (°C)	Ramp Rate (°C/min)	Time (min)
1	30	10	10

MP-Method

1,89666 nm and 1,89666 nm hydraulic radius: 0,000000 cm³/g

Average pore hydraulic radius (V/A): 0,00000 nm

Sample: resine Cagliari pre-macinazione
Operator:
Submitter:
File: C:\ASAP 2020 Plus\data\Chiappone\2022-1...\000-225.SMP

Started:	24/10/2022 09:45:14	Analysis adsorptive:	N2
Completed:	24/10/2022 18:12:00	Analysis bath temp.:	77,159 K
Report time:	24/10/2022 20:01:54	Thermal correction:	No
Sample mass:	0,4650 g	Ambient free space:	27,2106 cm³ Measured
Analysis free space:	81,8113 cm³	Equilibration interval:	10 s
Low pressure dose:	None	Sample density:	1,000 g/cm³
Automatic degas:	Yes		

Sample prep: Stage	Temperature (°C)	Ramp Rate (°C/min)	Time (min)
1	30	10	10

Isotherm Tabular Report

Relative Pressure (p/p°)	Absolute Pressure (mmHg)	Quantity Adsorbed (cm³/g STP)	Elapsed Time (h:min)	Saturation Pressure (mmHg)
			01:17	744.014038
0.010325733	7.684563	3.9811	01:37	744.214722
0.030151447	22.435867	5.1231	01:40	744.105835
0.065211970	48.522526	6.0868	01:44	744.073914
0.079628795	59.245651	6.3611	01:46	744.022949
0.100255334	74.600983	6.7047	01:48	744.109863
0.119977546	89.278870	6.9822	01:51	744.129822
0.140335176	104.428848	7.2456	01:53	744.138794
0.160395450	119.346550	7.4869	01:55	744.076904
0.179942753	133.891785	7.7117	01:58	744.079895
0.200404409	149.121490	7.9326	02:00	744.102844
0.249877845	185.936066	8.4467	02:02	744.107849
0.301219408	224.137924	8.9586	02:05	744.101868
0.351548232	261.575745	9.4673	02:07	744.067871
0.401182812	298.494019	9.9784	02:10	744.034912
0.451247824	335.705383	10.5169	02:12	743.949036
0.501321768	372.961365	11.0927	02:15	743.956055
0.551509705	410.304504	11.7071	02:17	743.966064
0.601477567	447.472290	12.3827	02:20	743.955078
0.651666999	484.788818	13.1553	02:23	743.921082
0.701675807	521.965515	14.0705	02:26	743.884155
0.751356749	558.938110	15.2306	02:29	743.905090
0.801005877	595.883545	16.9001	02:33	743.919067
0.821696069	611.328735	17.8894	02:36	743.984009
0.850785444	632.926575	19.8150	02:40	743.932068
0.875133065	650.938171	22.3095	02:45	743.816223
0.899472539	668.980286	26.3163	02:52	743.747314
0.923037342	686.279785	33.1115	03:01	743.501648
0.944354949	702.075684	45.3409	03:12	743.444702
0.962178157	715.373352	67.5952	03:30	743.493652
0.968536147	720.179810	81.8660	03:42	743.575562
0.975048333	724.679321	101.7588	04:02	743.223999
0.980299234	728.184448	122.4877	04:25	742.818542
0.983743039	730.651245	132.0595	04:40	742.725708
0.988803642	734.224243	140.4132	04:57	742.537964
0.973889494	723.118774	139.5939	05:01	742.505981
0.957042628	710.368103	132.5184	05:13	742.253357
0.947026856	702.753174	110.1270	05:31	742.062561
0.942708258	699.538147	89.7878	05:46	742.051575
0.934877335	693.391113	67.1028	06:10	741.692078

Sample: resine Cagliari pre-macinazione
Operator:
Submitter:
File: C:\ASAP 2020 Plus\data\Chiappone\2022-1...\000-225.SMP

Started:	24/10/2022 09:45:14	Analysis adsorptive:	N2
Completed:	24/10/2022 18:12:00	Analysis bath temp.:	77,159 K
Report time:	24/10/2022 20:01:54	Thermal correction:	No
Sample mass:	0,4650 g	Ambient free space:	27,2106 cm³ Measured
Analysis free space:	81,8113 cm³	Equilibration interval:	10 s
Low pressure dose:	None	Sample density:	1,000 g/cm³
Automatic degas:	Yes		

Sample prep: Stage	Temperature (°C)	Ramp Rate (°C/min)	Time (min)
1	30	10	10

Isotherm Tabular Report

Relative Pressure (p/p°)	Absolute Pressure (mmHg)	Quantity Adsorbed (cm³/g STP)	Elapsed Time (h:min)	Saturation Pressure (mmHg)
0.929256511	689.147949	58.2165	06:24	741.612183
0.906389353	672.129639	38.1797	06:46	741.546265
0.870708549	645.717651	25.9250	07:03	741.600220
0.845817091	627.197327	21.9115	07:10	741.528320
0.816648258	605.574341	18.9695	07:16	741.536316
0.803306893	595.634705	18.0223	07:20	741.478394
0.749405336	555.670837	15.5270	07:24	741.482361
0.699621013	518.790161	14.2010	07:27	741.530273
0.650655650	482.463348	13.2657	07:30	741.503357
0.599337509	444.412567	12.4571	07:33	741.506348
0.549150094	407.197174	11.7657	07:36	741.504333
0.499131913	370.083069	11.1466	07:38	741.453430
0.449286253	333.108215	10.5723	07:40	741.416443
0.398859874	295.701355	10.0243	07:43	741.366516
0.348925372	258.723755	9.5031	07:45	741.487366
0.299235834	221.848816	9.0015	07:47	741.384521
0.249335397	184.868835	8.5012	07:50	741.446411
0.199566816	147.951157	7.9859	07:53	741.361511
0.139705208	103.565788	7.3060	07:57	741.316589
0.099980786	74.120613	6.7675	08:02	741.348572

Sample: resine Cagliari pre-macinazione
Operator:
Submitter:
File: C:\ASAP 2020 Plus\data\Chiappone\2022-1...\000-225.SMP

Started:	24/10/2022 09:45:14	Analysis adsorptive:	N2
Completed:	24/10/2022 18:12:00	Analysis bath temp.:	77,159 K
Report time:	24/10/2022 20:01:54	Thermal correction:	No
Sample mass:	0,4650 g	Ambient free space:	27,2106 cm³ Measured
Analysis free space:	81,8113 cm³	Equilibration interval:	10 s
Low pressure dose:	None	Sample density:	1,000 g/cm³
Automatic degas:	Yes		

Sample prep: Stage	Temperature (°C)	Ramp Rate (°C/min)	Time (min)
1	30	10	10

Isotherm Linear Plot

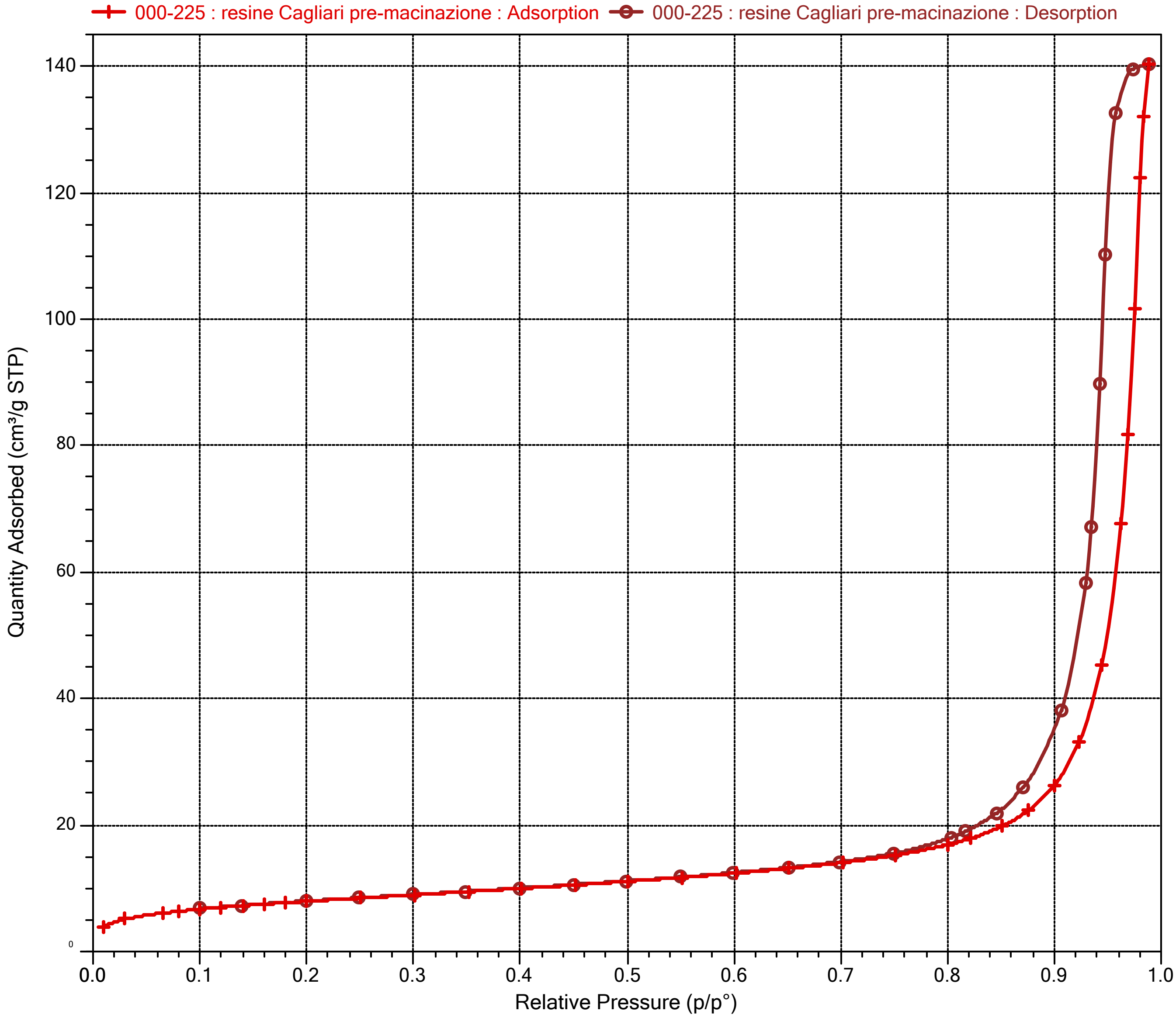

Sample: resine Cagliari pre-macinazione
Operator:
Submitter:
File: C:\ASAP 2020 Plus\data\Chiappone\2022-1...\000-225.SMP

Started:	24/10/2022 09:45:14	Analysis adsorptive:	N2
Completed:	24/10/2022 18:12:00	Analysis bath temp.:	77,159 K
Report time:	24/10/2022 20:01:54	Thermal correction:	No
Sample mass:	0,4650 g	Ambient free space:	27,2106 cm³ Measured
Analysis free space:	81,8113 cm³	Equilibration interval:	10 s
Low pressure dose:	None	Sample density:	1,000 g/cm³
Automatic degas:	Yes		

Sample prep: Stage	Temperature (°C)	Ramp Rate (°C/min)	Time (min)
1	30	10	10

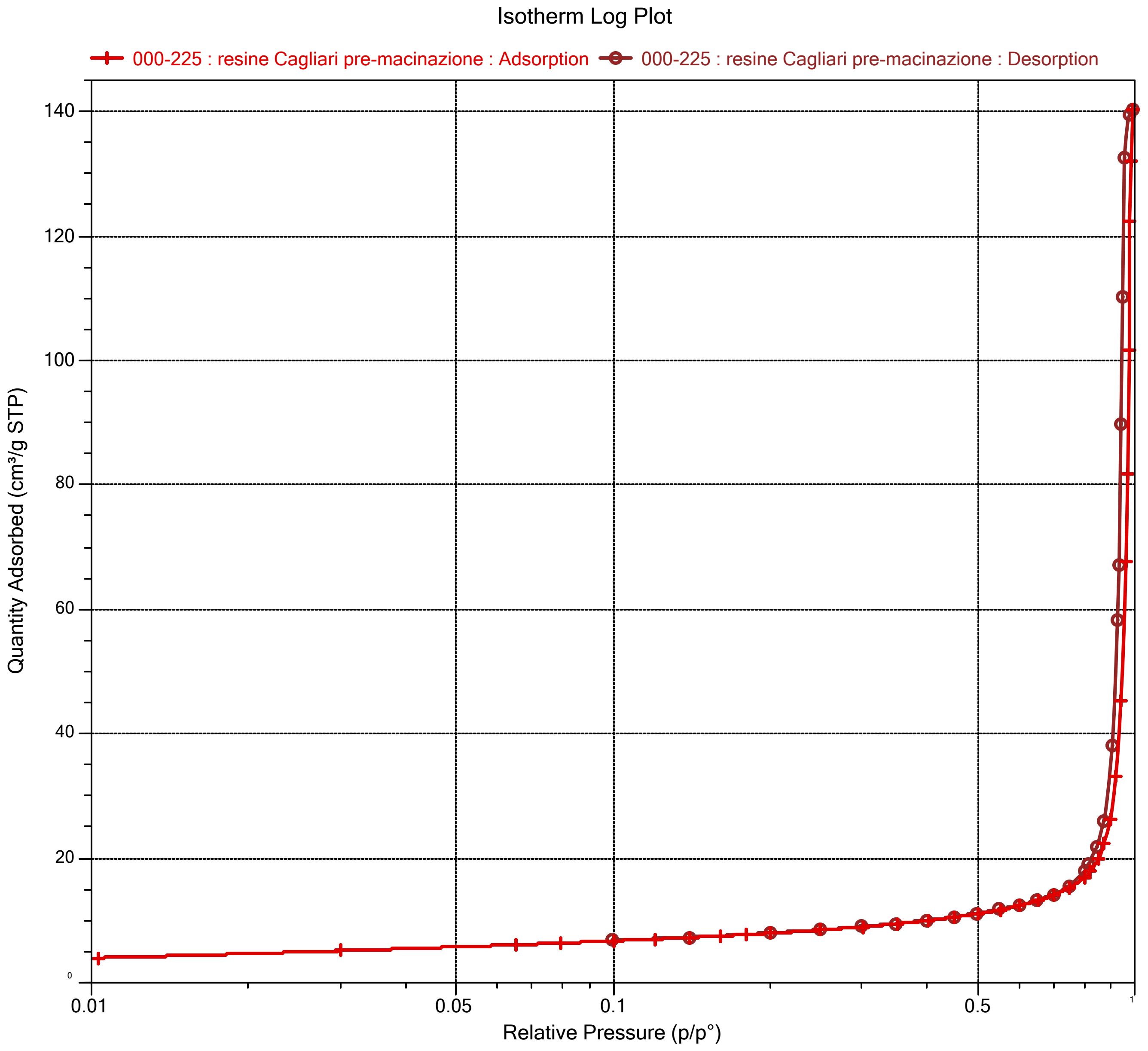

micromeritics®

Sample: resine Cagliari pre-macinazione
Operator:
Submitter:
File: C:\ASAP 2020 Plus\data\Chiappone\2022-1...\000-225.SMP

Started:	24/10/2022 09:45:14	Analysis adsorptive:	N2
Completed:	24/10/2022 18:12:00	Analysis bath temp.:	77,159 K
Report time:	24/10/2022 20:01:54	Thermal correction:	No
Sample mass:	0,4650 g	Ambient free space:	27,2106 cm³ Measured
Analysis free space:	81,8113 cm³	Equilibration interval:	10 s
Low pressure dose:	None	Sample density:	1,000 g/cm³
Automatic degas:	Yes		

Sample prep: Stage	Temperature (°C)	Ramp Rate (°C/min)	Time (min)
1	30	10	10

Isotherm Linear Absolute Plot

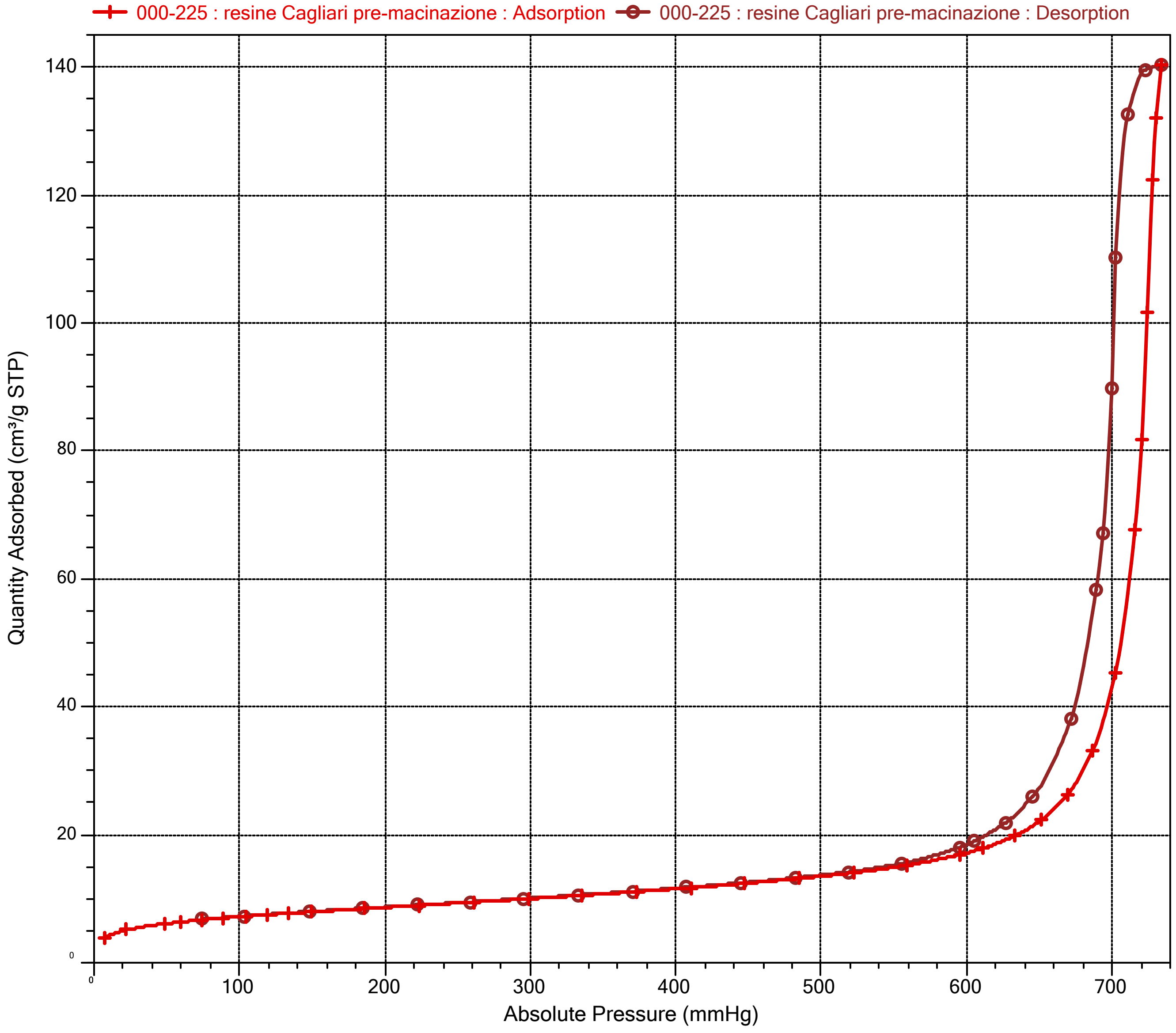

Sample: resine Cagliari pre-macinazione
Operator:
Submitter:
File: C:\ASAP 2020 Plus\data\Chiappone\2022-1...\000-225.SMP

Started:	24/10/2022 09:45:14	Analysis adsorptive:	N2
Completed:	24/10/2022 18:12:00	Analysis bath temp.:	77,159 K
Report time:	24/10/2022 20:01:54	Thermal correction:	No
Sample mass:	0,4650 g	Ambient free space:	27,2106 cm³ Measured
Analysis free space:	81,8113 cm³	Equilibration interval:	10 s
Low pressure dose:	None	Sample density:	1,000 g/cm³
Automatic degas:	Yes		

Sample prep: Stage	Temperature (°C)	Ramp Rate (°C/min)	Time (min)
1	30	10	10

Isotherm Log Absolute Plot

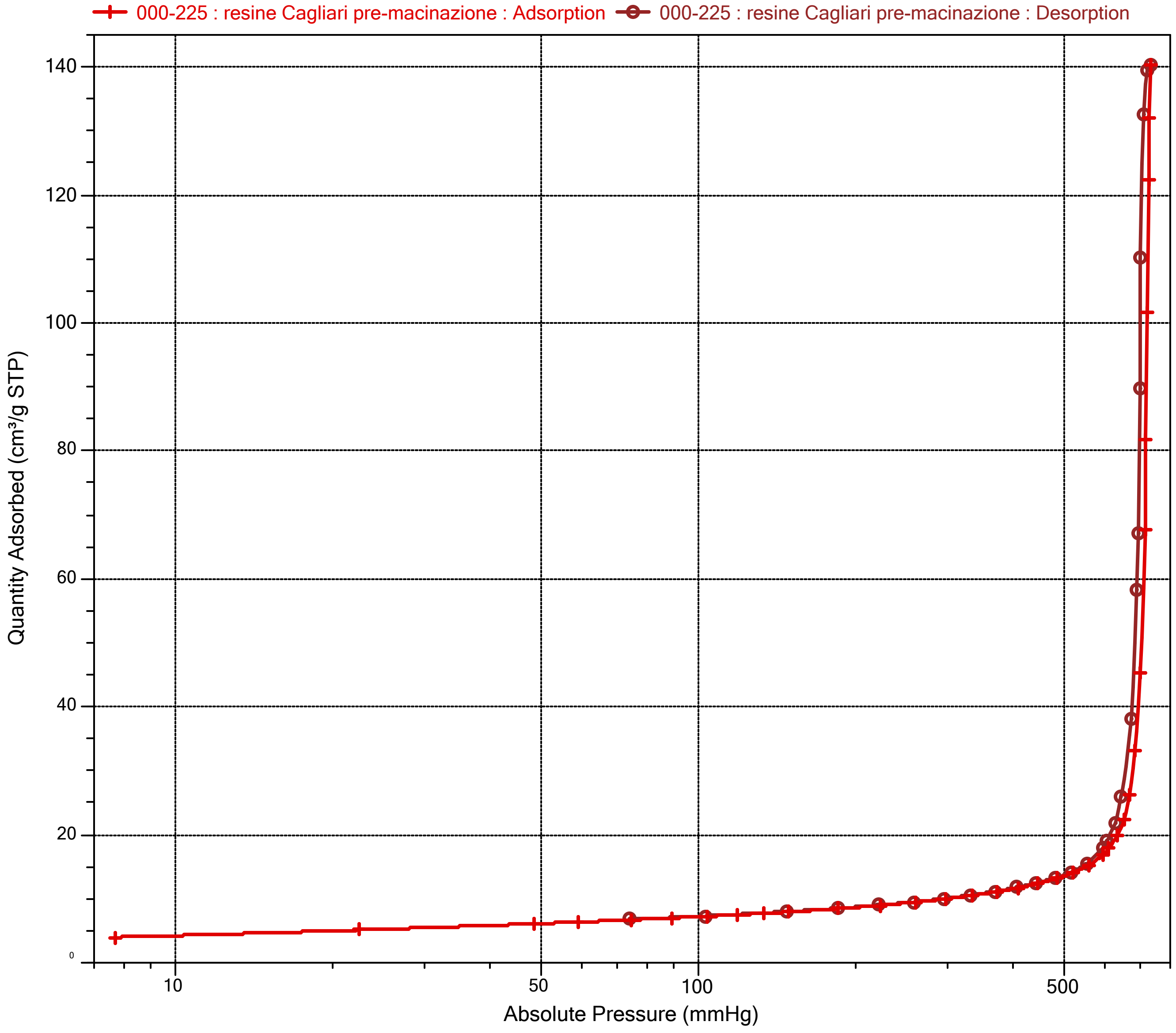

Sample: resine Cagliari pre-macinazione
Operator:
Submitter:
File: C:\ASAP 2020 Plus\data\Chiappone\2022-1...\000-225.SMP

Started:	24/10/2022 09:45:14	Analysis adsorptive:	N2
Completed:	24/10/2022 18:12:00	Analysis bath temp.:	77,159 K
Report time:	24/10/2022 20:01:54	Thermal correction:	No
Sample mass:	0,4650 g	Ambient free space:	27,2106 cm³ Measured
Analysis free space:	81,8113 cm³	Equilibration interval:	10 s
Low pressure dose:	None	Sample density:	1,000 g/cm³
Automatic degas:	Yes		

Sample prep: Stage	Temperature (°C)	Ramp Rate (°C/min)	Time (min)
1	30	10	10

Isotherm Pressure Composition

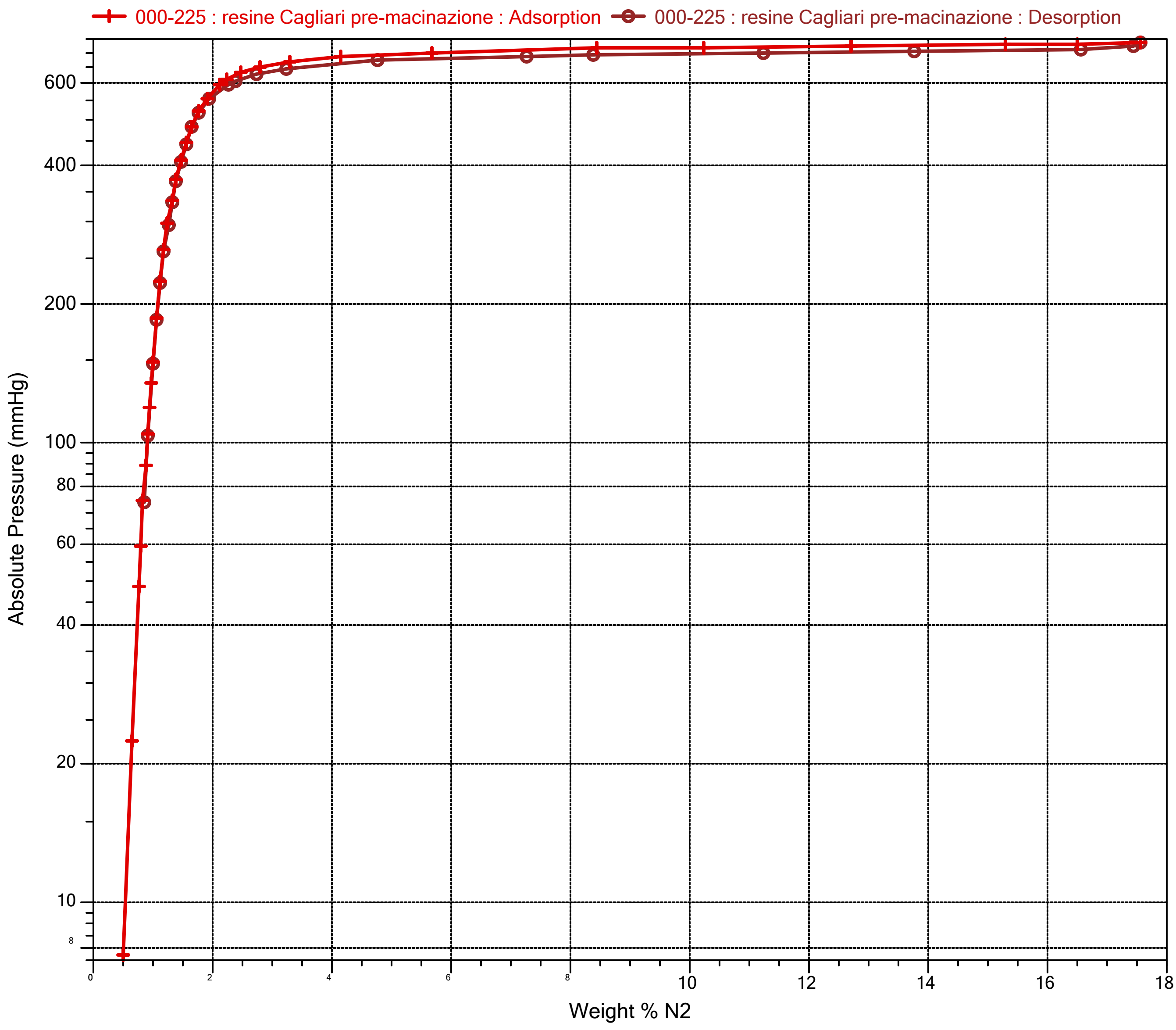

Sample: resine Cagliari pre-macinazione
Operator:
Submitter:
File: C:\ASAP 2020 Plus\data\Chiappone\2022-1...\000-225.SMP

Started:	24/10/2022 09:45:14	Analysis adsorptive:	N2
Completed:	24/10/2022 18:12:00	Analysis bath temp.:	77,159 K
Report time:	24/10/2022 20:01:54	Thermal correction:	No
Sample mass:	0,4650 g	Ambient free space:	27,2106 cm³ Measured
Analysis free space:	81,8113 cm³	Equilibration interval:	10 s
Low pressure dose:	None	Sample density:	1,000 g/cm³
Automatic degas:	Yes		

Sample prep: Stage	Temperature (°C)	Ramp Rate (°C/min)	Time (min)
1	30	10	10

BET Report

BET surface area: 28,6015 ± 0,1825 m²/g
Slope: 0,150686 ± 0,000960 g/cm³ STP
Y-intercept: 0,001494 ± 0,000148 g/cm³ STP
C: 101,859846
Qm: 6,5712 cm³/g STP
Correlation coefficient: 0,9998581
Molecular cross-sectional area: 0,1620 nm²

Relative Pressure (p/p°)	Quantity Adsorbed (cm³/g STP)	1/[Q(p°/p - 1)]
0.065211970	6.0868	0.011461
0.079628795	6.3611	0.013601
0.100255334	6.7047	0.016619
0.119977546	6.9822	0.019526
0.140335176	7.2456	0.022530
0.160395450	7.4869	0.025516
0.179942753	7.7117	0.028454
0.200404409	7.9326	0.031595
0.249877845	8.4467	0.039437

Sample: resine Cagliari pre-macinazione
Operator:
Submitter:
File: C:\ASAP 2020 Plus\data\Chiappone\2022-1...\000-225.SMP

Started:	24/10/2022 09:45:14	Analysis adsorptive:	N2
Completed:	24/10/2022 18:12:00	Analysis bath temp.:	77,159 K
Report time:	24/10/2022 20:01:54	Thermal correction:	No
Sample mass:	0,4650 g	Ambient free space:	27,2106 cm³ Measured
Analysis free space:	81,8113 cm³	Equilibration interval:	10 s
Low pressure dose:	None	Sample density:	1,000 g/cm³
Automatic degas:	Yes		

Sample prep: Stage	Temperature (°C)	Ramp Rate (°C/min)	Time (min)
1	30	10	10

BET Surface Area Plot

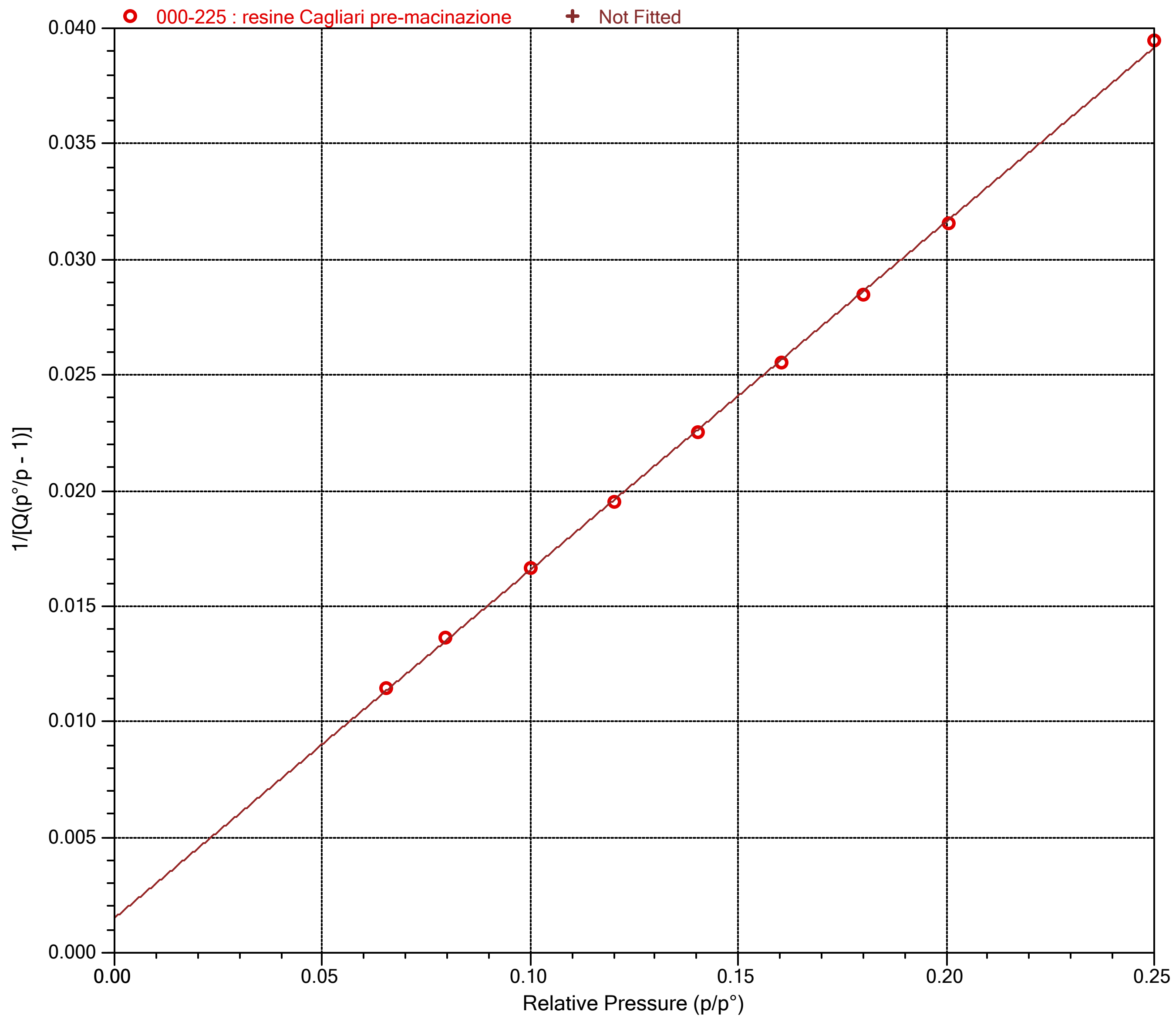

Sample: resine Cagliari pre-macinazione
Operator:
Submitter:
File: C:\ASAP 2020 Plus\data\Chiappone\2022-1...\000-225.SMP

Started:	24/10/2022 09:45:14	Analysis adsorptive:	N2
Completed:	24/10/2022 18:12:00	Analysis bath temp.:	77,159 K
Report time:	24/10/2022 20:01:54	Thermal correction:	No
Sample mass:	0,4650 g	Ambient free space:	27,2106 cm³ Measured
Analysis free space:	81,8113 cm³	Equilibration interval:	10 s
Low pressure dose:	None	Sample density:	1,000 g/cm³
Automatic degas:	Yes		

Sample prep: Stage	Temperature (°C)	Ramp Rate (°C/min)	Time (min)
1	30	10	10

Langmuir Report

Langmuir surface area: 535,7181 ± 513,4384 m²/g
Slope: 0,008125 ± 0,007787 g/cm³ STP
Y-intercept: 17,328 ± 3,751 g/cm³ STP·mmHg
b: 0,000469 1/mmHg
Qm: 123,0806 cm³/g STP
Correlation coefficient: 0,181388
Molecular cross-sectional area: 0,1620 nm²

Pressure (mmHg)	Quantity Adsorbed (cm³/g STP)	p/Q (g/cm³ STP·mmHg)
7.684563	3.9811	1.930
22.435867	5.1231	4.379
48.522526	6.0868	7.972
59.245651	6.3611	9.314
74.600983	6.7047	11.127
89.278870	6.9822	12.787
104.428848	7.2456	14.413
119.346550	7.4869	15.941
133.891785	7.7117	17.362
149.121490	7.9326	18.799
185.936066	8.4467	22.013
224.137924	8.9586	25.019
261.575745	9.4673	27.630
298.494019	9.9784	29.914
335.705383	10.5169	31.921
372.961365	11.0927	33.622
410.304504	11.7071	35.048
447.472290	12.3827	36.137
484.788818	13.1553	36.851
521.965515	14.0705	37.096
558.938110	15.2306	36.698
595.883545	16.9001	35.259
611.328735	17.8894	34.173
632.926575	19.8150	31.942
650.938171	22.3095	29.178
668.980286	26.3163	25.421
686.279785	33.1115	20.726
702.075684	45.3409	15.484
715.373352	67.5952	10.583
720.179810	81.8660	8.797
724.679321	101.7588	7.122
728.184448	122.4877	5.945

Sample: resine Cagliari pre-macinazione
Operator:
Submitter:
File: C:\ASAP 2020 Plus\data\Chiappone\2022-1...\000-225.SMP

Started:	24/10/2022 09:45:14	Analysis adsorptive:	N2
Completed:	24/10/2022 18:12:00	Analysis bath temp.:	77,159 K
Report time:	24/10/2022 20:01:54	Thermal correction:	No
Sample mass:	0,4650 g	Ambient free space:	27,2106 cm³ Measured
Analysis free space:	81,8113 cm³	Equilibration interval:	10 s
Low pressure dose:	None	Sample density:	1,000 g/cm³
Automatic degas:	Yes		

Sample prep: Stage	Temperature (°C)	Ramp Rate (°C/min)	Time (min)
1	30	10	10

Pressure (mmHg)	Quantity Adsorbed (cm³/g STP)	p/Q (g/cm³ STP·mmHg)
730.651245	132.0595	5.533
734.224243	140.4132	5.229

Sample: resine Cagliari pre-macinazione
Operator:
Submitter:
File: C:\ASAP 2020 Plus\data\Chiappone\2022-1...\000-225.SMP

Started:	24/10/2022 09:45:14	Analysis adsorptive:	N2
Completed:	24/10/2022 18:12:00	Analysis bath temp.:	77,159 K
Report time:	24/10/2022 20:01:54	Thermal correction:	No
Sample mass:	0,4650 g	Ambient free space:	27,2106 cm³ Measured
Analysis free space:	81,8113 cm³	Equilibration interval:	10 s
Low pressure dose:	None	Sample density:	1,000 g/cm³
Automatic degas:	Yes		

Sample prep: Stage	Temperature (°C)	Ramp Rate (°C/min)	Time (min)
1	30	10	10

Langmuir Surface Area Plot

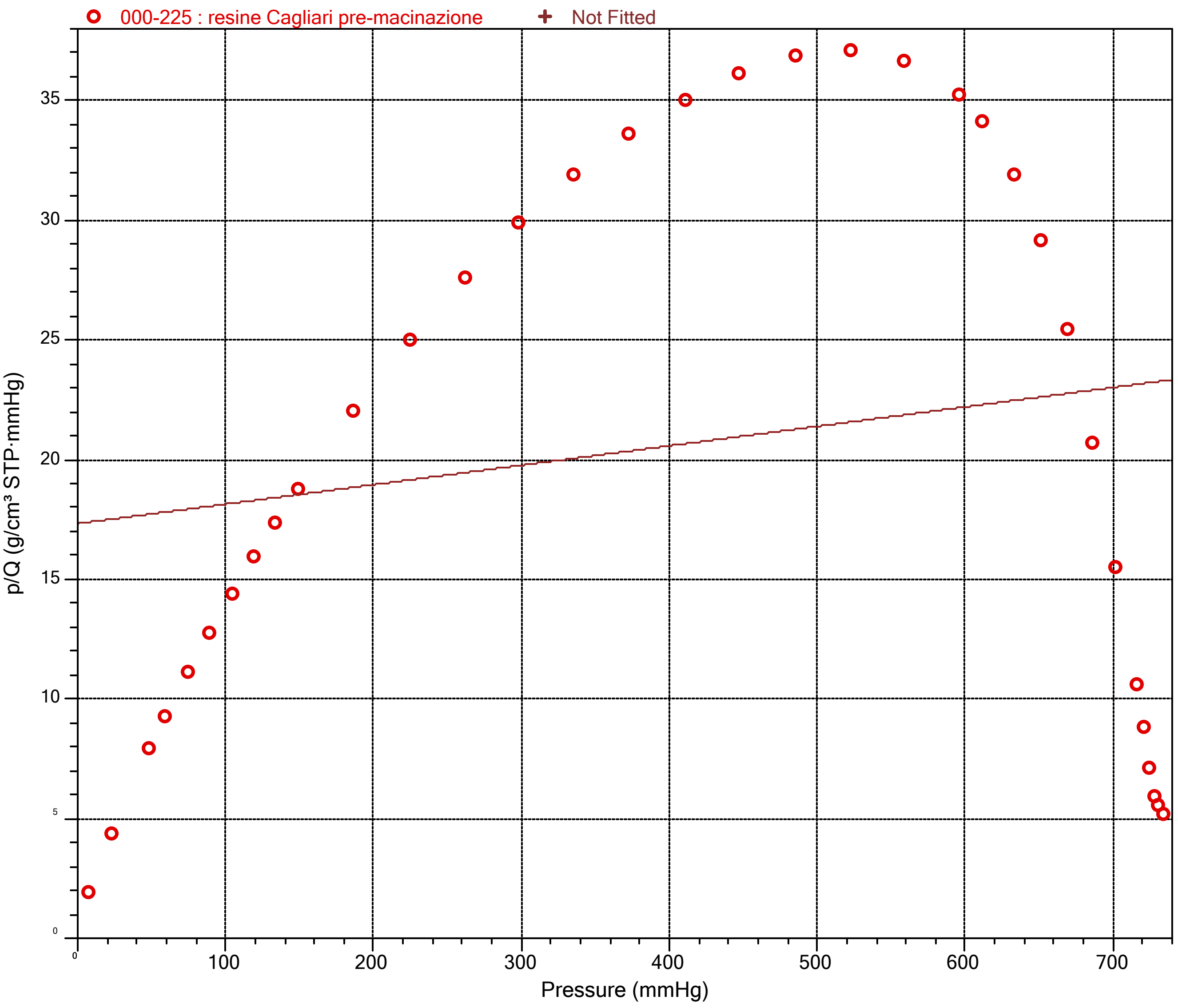

Sample: resine Cagliari pre-macinazione
Operator:
Submitter:
File: C:\ASAP 2020 Plus\data\Chiappone\2022-1...\000-225.SMP

Started:	24/10/2022 09:45:14	Analysis adsorptive:	N2
Completed:	24/10/2022 18:12:00	Analysis bath temp.:	77,159 K
Report time:	24/10/2022 20:01:54	Thermal correction:	No
Sample mass:	0,4650 g	Ambient free space:	27,2106 cm³ Measured
Analysis free space:	81,8113 cm³	Equilibration interval:	10 s
Low pressure dose:	None	Sample density:	1,000 g/cm³
Automatic degas:	Yes		

Sample prep: Stage	Temperature (°C)	Ramp Rate (°C/min)	Time (min)
1	30	10	10

Freundlich Tabular Report

Qm·C: 0,3865 ± 0,0996 cm³/g STP
m: 1,4920 ± 0,2311
Correlation coefficient: 0,752104

Absolute Pressure (mmHg)	Quantity Adsorbed (cm³/g STP)	log(p)	log(Q)
7.684563	3.9811	0.88562	0.6000
22.435867	5.1231	1.35094	0.7095
48.522526	6.0868	1.68594	0.7844
59.245651	6.3611	1.77266	0.8035
74.600983	6.7047	1.87274	0.8264
89.278870	6.9822	1.95075	0.8440
104.428848	7.2456	2.01882	0.8601
119.346550	7.4869	2.07681	0.8743
133.891785	7.7117	2.12675	0.8872
149.121490	7.9326	2.17354	0.8994
185.936066	8.4467	2.26936	0.9267
224.137924	8.9586	2.35052	0.9522
261.575745	9.4673	2.41760	0.9762
298.494019	9.9784	2.47494	0.9991
335.705383	10.5169	2.52596	1.0219
372.961365	11.0927	2.57166	1.0450
410.304504	11.7071	2.61311	1.0684
447.472290	12.3827	2.65077	1.0928
484.788818	13.1553	2.68555	1.1191
521.965515	14.0705	2.71764	1.1483
558.938110	15.2306	2.74736	1.1827
595.883545	16.9001	2.77516	1.2279
611.328735	17.8894	2.78627	1.2526
632.926575	19.8150	2.80135	1.2970
650.938171	22.3095	2.81354	1.3485
668.980286	26.3163	2.82541	1.4202
686.279785	33.1115	2.83650	1.5200
702.075684	45.3409	2.84638	1.6565
715.373352	67.5952	2.85453	1.8299
720.179810	81.8660	2.85744	1.9131
724.679321	101.7588	2.86015	2.0076
728.184448	122.4877	2.86224	2.0881
730.651245	132.0595	2.86371	2.1208
734.224243	140.4132	2.86583	2.1474

Sample: resine Cagliari pre-macinazione
Operator:
Submitter:
File: C:\ASAP 2020 Plus\data\Chiappone\2022-1...\000-225.SMP

Started:	24/10/2022 09:45:14	Analysis adsorptive:	N2
Completed:	24/10/2022 18:12:00	Analysis bath temp.:	77,159 K
Report time:	24/10/2022 20:01:54	Thermal correction:	No
Sample mass:	0,4650 g	Ambient free space:	27,2106 cm³ Measured
Analysis free space:	81,8113 cm³	Equilibration interval:	10 s
Low pressure dose:	None	Sample density:	1,000 g/cm³
Automatic degas:	Yes		

Sample prep: Stage	Temperature (°C)	Ramp Rate (°C/min)	Time (min)
1	30	10	10

Freundlich Plot

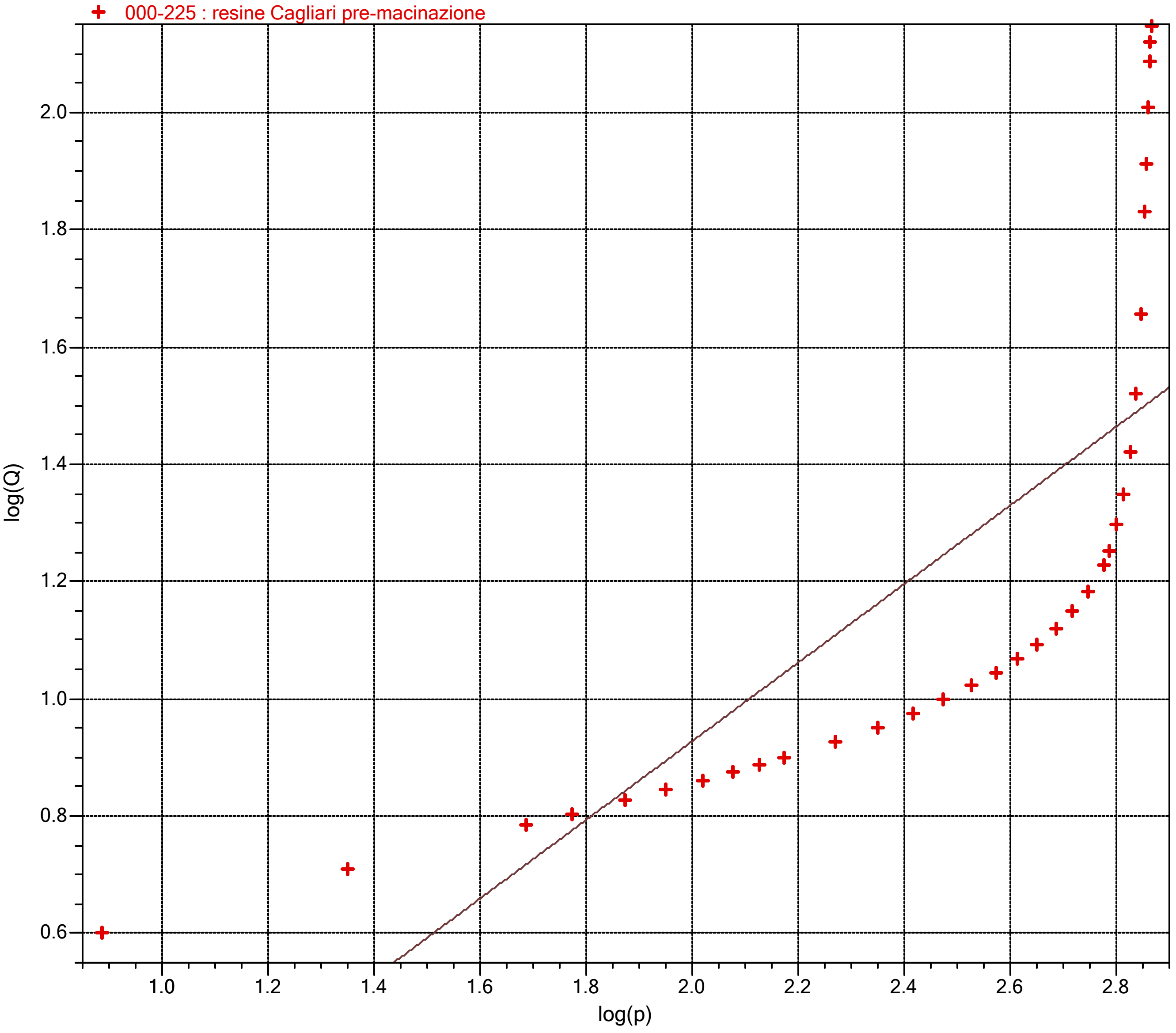

Sample: resine Cagliari pre-macinazione
Operator:
Submitter:
File: C:\ASAP 2020 Plus\data\Chiappone\2022-1...\000-225.SMP

Started:	24/10/2022 09:45:14	Analysis adsorptive:	N2
Completed:	24/10/2022 18:12:00	Analysis bath temp.:	77,159 K
Report time:	24/10/2022 20:01:54	Thermal correction:	No
Sample mass:	0,4650 g	Ambient free space:	27,2106 cm³ Measured
Analysis free space:	81,8113 cm³	Equilibration interval:	10 s
Low pressure dose:	None	Sample density:	1,000 g/cm³
Automatic degas:	Yes		

Sample prep: Stage	Temperature (°C)	Ramp Rate (°C/min)	Time (min)
1	30	10	10

t-Plot Report

Micropore volume:	0,000298 cm³/g
Micropore area:	1,2312 m²/g
External surface area:	27,3703 m²/g
Slope:	17,694798 ± 0,317361 cm³/g·nm STP
Y-intercept:	0,192358 ± 0,129039 cm³/g STP
Correlation coefficient:	0,999036
Surface area correction factor:	1,000
Density conversion factor:	0,0015468
Total surface area (BET):	28,6015 m²/g
Thickness range:	0,35000 to 0,50000 nm
Thickness equation:	Harkins and Jura

Thickness Curve

$$t = [\ 13.99 / (\ 0.034 - \log(p/p°)\ )\ ]\ \hat{}\ 0.5$$

t-Plot Report - Data

Relative Pressure (p/p°)	Statistical Thickness (nm)	Quantity Adsorbed (cm³/g STP)	Fitted
0.065211970	0.33868	6.0868	
0.079628795	0.35140	6.3611	·
0.100255334	0.36803	6.7047	·
0.119977546	0.38276	6.9822	·
0.140335176	0.39718	7.2456	·
0.160395450	0.41085	7.4869	·
0.179942753	0.42382	7.7117	·
0.200404409	0.43715	7.9326	·
0.249877845	0.46891	8.4467	·
0.301219408	0.50201	8.9586	
0.351548232	0.53542	9.4673	
0.401182812	0.56996	9.9784	
0.451247824	0.60709	10.5169	
0.501321768	0.64731	11.0927	
0.551509705	0.69165	11.7071	
0.601477567	0.74101	12.3827	
0.651666999	0.79749	13.1553	

Sample: resine Cagliari pre-macinazione
Operator:
Submitter:
File: C:\ASAP 2020 Plus\data\Chiappone\2022-1...\000-225.SMP

Started:	24/10/2022 09:45:14	Analysis adsorptive:	N2
Completed:	24/10/2022 18:12:00	Analysis bath temp.:	77,159 K
Report time:	24/10/2022 20:01:54	Thermal correction:	No
Sample mass:	0,4650 g	Ambient free space:	27,2106 cm³ Measured
Analysis free space:	81,8113 cm³	Equilibration interval:	10 s
Low pressure dose:	None	Sample density:	1,000 g/cm³
Automatic degas:	Yes		

Sample prep: Stage	Temperature (°C)	Ramp Rate (°C/min)	Time (min)
1	30	10	10

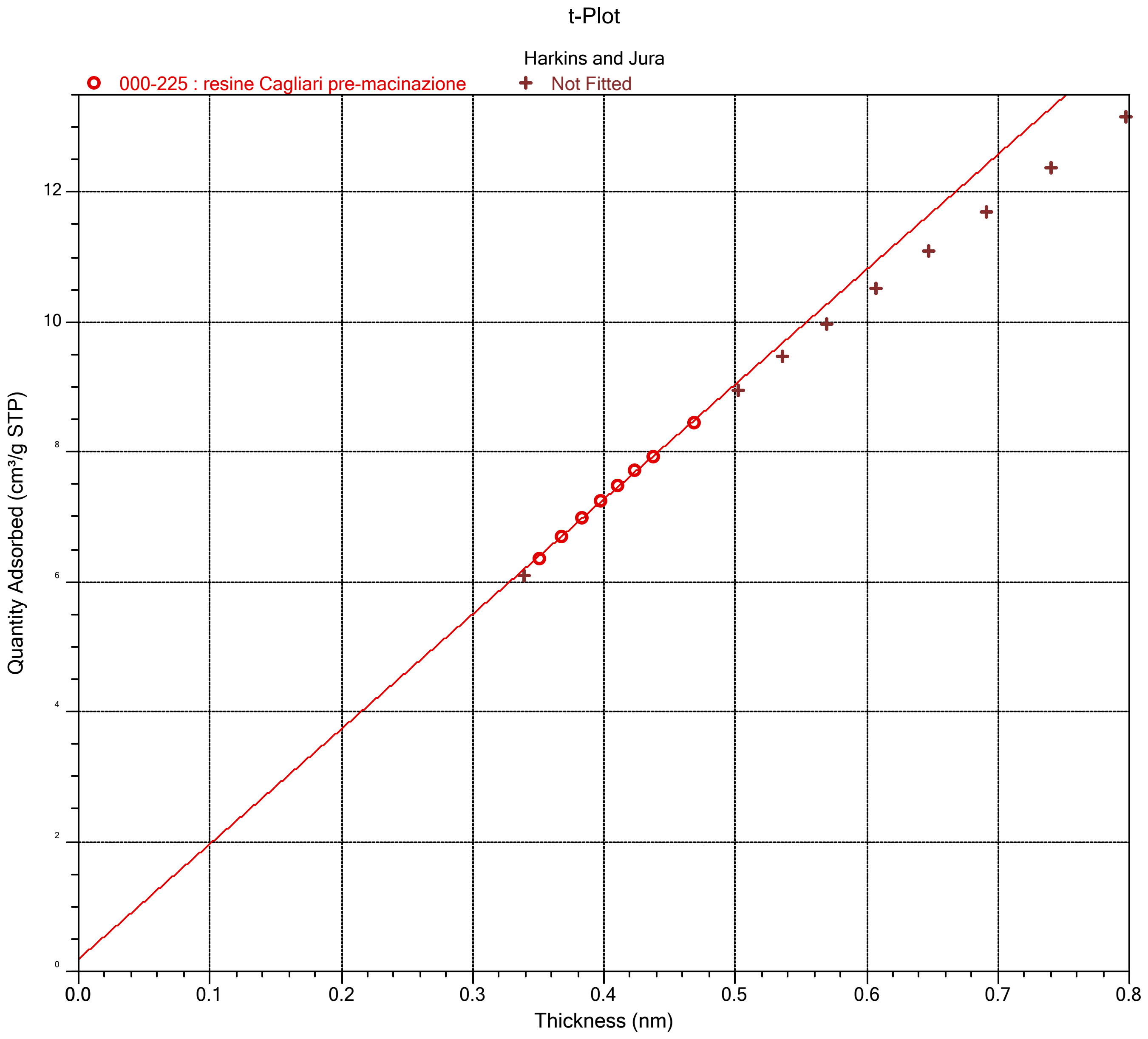

Sample: resine Cagliari pre-macinazione
Operator:
Submitter:
File: C:\ASAP 2020 Plus\data\Chiappone\2022-1...\000-225.SMP

Started:	24/10/2022 09:45:14	Analysis adsorptive:	N2
Completed:	24/10/2022 18:12:00	Analysis bath temp.:	77,159 K
Report time:	24/10/2022 20:01:54	Thermal correction:	No
Sample mass:	0,4650 g	Ambient free space:	27,2106 cm³ Measured
Analysis free space:	81,8113 cm³	Equilibration interval:	10 s
Low pressure dose:	None	Sample density:	1,000 g/cm³
Automatic degas:	Yes		

Sample prep: Stage	Temperature (°C)	Ramp Rate (°C/min)	Time (min)
1	30	10	10

BJH Adsorption Pore Distribution Report

Faas Correction
Harkins and Jura
t = [13.99 / (0.034 - log(p/p°))] ^ 0.5

Width range: 1,7000 to 300,0000 nm
Adsorbate property factor: 0,95300 nm
Density conversion factor: 0,0015468
Fraction of pores open at both ends: 0,00

Pore Width Range (nm)	Average Width (nm)	Incremental Pore Volume (cm³/g)	Cumulative Pore Volume (cm³/g)	Incremental Pore Area (m²/g)	Cumulative Pore Area (m²/g)
173.1 - 120.0	137.0	0.013646	0.013646	0.398	0.398
120.0 - 99.4	107.7	0.015840	0.029487	0.588	0.987
99.4 - 79.0	86.8	0.034788	0.064275	1.603	2.590
79.0 - 63.0	69.2	0.033903	0.098178	1.961	4.551
63.0 - 52.8	56.9	0.024652	0.122830	1.732	6.282
52.8 - 36.4	41.5	0.039337	0.162167	3.792	10.074
36.4 - 26.7	30.0	0.021762	0.183929	2.906	12.980
26.7 - 20.6	22.8	0.011820	0.195749	2.071	15.051
20.6 - 16.8	18.3	0.006612	0.202361	1.448	16.499
16.8 - 14.1	15.2	0.003765	0.206126	0.992	17.490
14.1 - 11.9	12.8	0.002520	0.208645	0.789	18.279
11.9 - 10.7	11.2	0.001096	0.209741	0.392	18.671
10.7 - 8.5	9.3	0.001435	0.211176	0.614	19.285
8.5 - 7.1	7.7	0.000653	0.211829	0.340	19.625
7.1 - 6.0	6.5	0.000367	0.212196	0.227	19.852
6.0 - 5.2	5.6	0.000258	0.212455	0.186	20.037
5.2 - 4.6	4.9	0.000218	0.212672	0.179	20.216
4.6 - 4.1	4.3	0.000211	0.212883	0.197	20.413
4.1 - 3.6	3.8	0.000245	0.213128	0.258	20.671
3.6 - 3.2	3.4	0.000241	0.213369	0.284	20.955
3.2 - 2.9	3.0	0.000252	0.213621	0.332	21.287
2.9 - 2.6	2.7	0.000288	0.213909	0.423	21.710
2.6 - 2.3	2.4	0.000303	0.214212	0.498	22.208
2.3 - 2.1	2.2	0.000372	0.214584	0.687	22.895
2.1 - 2.0	2.0	0.000172	0.214756	0.342	23.237
2.0 - 1.9	1.9	0.000209	0.214965	0.439	23.675
1.9 - 1.8	1.8	0.000235	0.215200	0.518	24.194
1.8 - 1.7	1.7	0.000284	0.215483	0.663	24.857

Sample: resine Cagliari pre-macinazione
Operator:
Submitter:
File: C:\ASAP 2020 Plus\data\Chiappone\2022-1...\000-225.SMP

Started:	24/10/2022 09:45:14	Analysis adsorptive:	N2
Completed:	24/10/2022 18:12:00	Analysis bath temp.:	77,159 K
Report time:	24/10/2022 20:01:54	Thermal correction:	No
Sample mass:	0,4650 g	Ambient free space:	27,2106 cm³ Measured
Analysis free space:	81,8113 cm³	Equilibration interval:	10 s
Low pressure dose:	None	Sample density:	1,000 g/cm³
Automatic degas:	Yes		

Sample prep: Stage	Temperature (°C)	Ramp Rate (°C/min)	Time (min)
1	30	10	10

BJH Adsorption Cumulative Pore Volume (Larger)

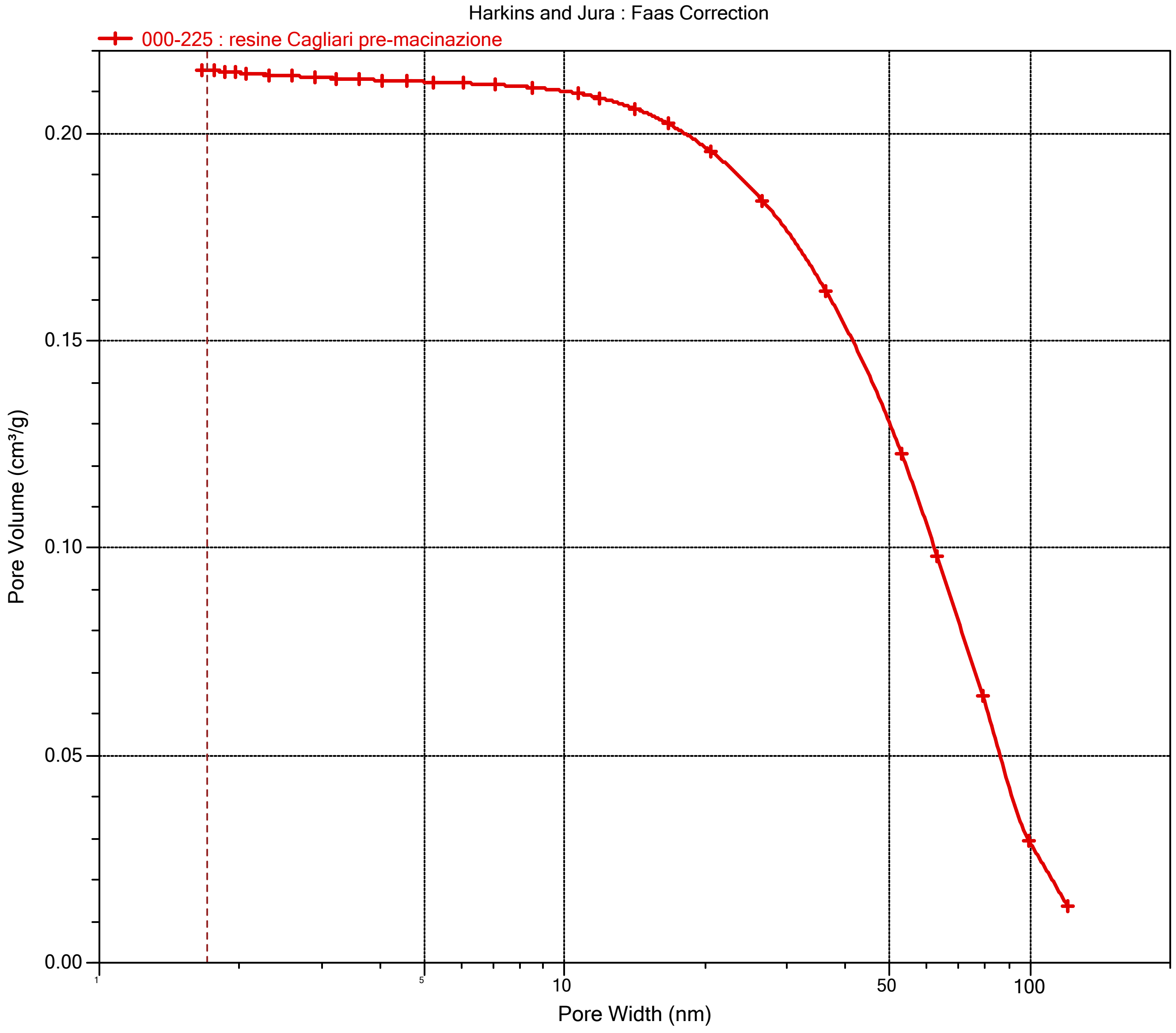

Sample:	resine Cagliari pre-macinazione
Operator:	
Submitter:	
File:	C:\ASAP 2020 Plus\data\Chiappone\2022-1...\000-225.SMP

Started:	24/10/2022 09:45:14	Analysis adsorptive:	N2
Completed:	24/10/2022 18:12:00	Analysis bath temp.:	77,159 K
Report time:	24/10/2022 20:01:54	Thermal correction:	No
Sample mass:	0,4650 g	Ambient free space:	27,2106 cm³ Measured
Analysis free space:	81,8113 cm³	Equilibration interval:	10 s
Low pressure dose:	None	Sample density:	1,000 g/cm³
Automatic degas:	Yes		

Sample prep: Stage	Temperature (°C)	Ramp Rate (°C/min)	Time (min)
1	30	10	10

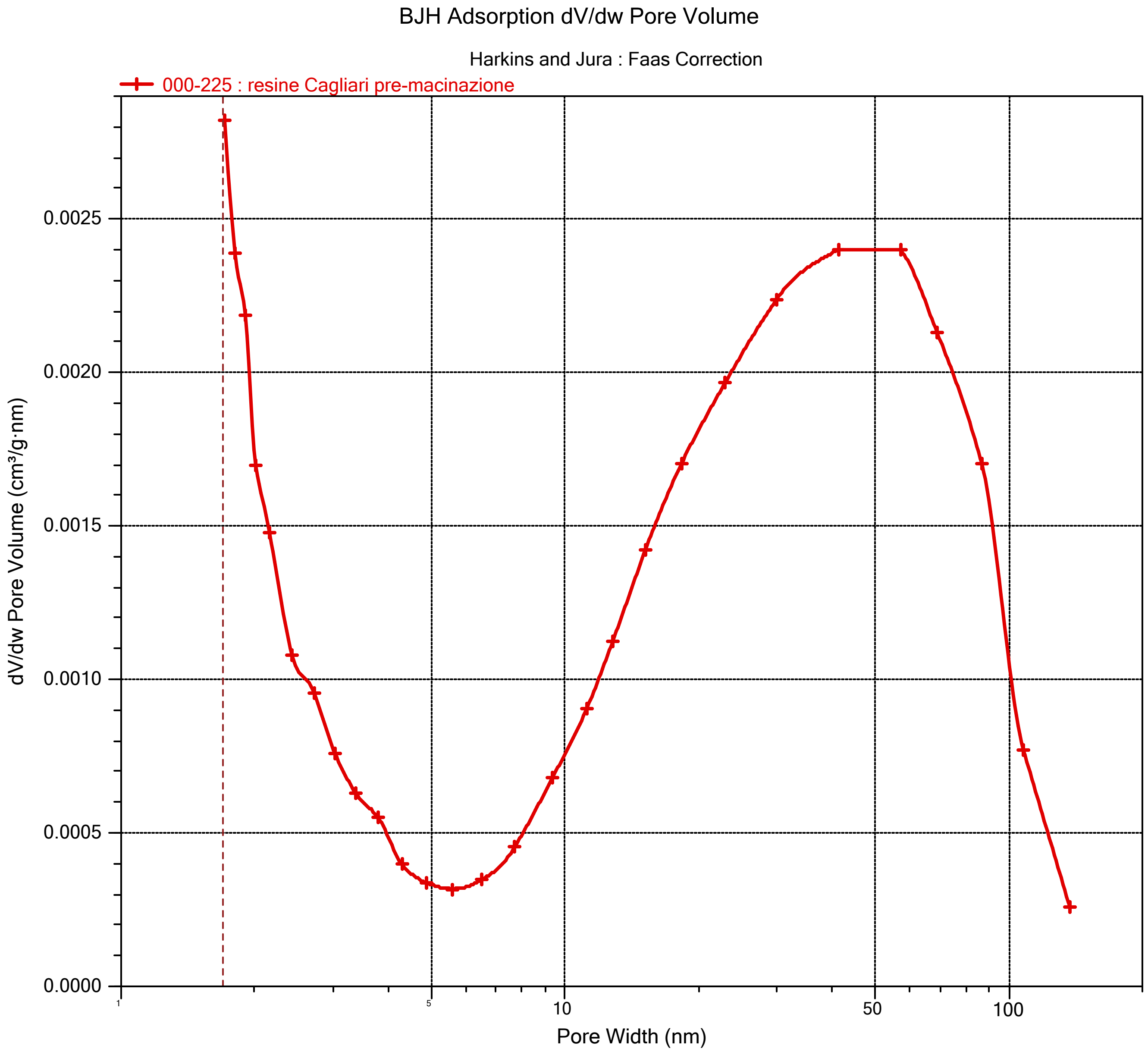

Sample: resine Cagliari pre-macinazione
Operator:
Submitter:
File: C:\ASAP 2020 Plus\data\Chiappone\2022-1...\000-225.SMP

Started: 24/10/2022 09:45:14
Completed: 24/10/2022 18:12:00
Report time: 24/10/2022 20:01:54
Sample mass: 0,4650 g
Analysis free space: 81,8113 cm³
Low pressure dose: None
Automatic degas: Yes

Analysis adsorptive: N2
Analysis bath temp.: 77,159 K
Thermal correction: No
Ambient free space: 27,2106 cm³ Measured
Equilibration interval: 10 s
Sample density: 1,000 g/cm³

Sample prep: Stage	Temperature (°C)	Ramp Rate (°C/min)	Time (min)
1	30	10	10

BJH Desorption Pore Distribution Report

Faas Correction
Harkins and Jura
t = [13.99 / (0.034 - log(p/p°))] ^ 0.5

Width range: 1,7000 to 300,0000 nm
Adsorbate property factor: 0,95300 nm
Density conversion factor: 0,0015468
Fraction of pores open at both ends: 0,00

Pore Width Range (nm)	Average Width (nm)	Incremental Pore Volume (cm³/g)	Cumulative Pore Volume (cm³/g)	Incremental Pore Area (m²/g)	Cumulative Pore Area (m²/g)
172.8 - 75.3	90.3	0.001364	0.001364	0.060	0.060
75.3 - 46.4	54.1	0.012263	0.013627	0.906	0.966
46.4 - 37.9	41.3	0.039958	0.053585	3.873	4.840
37.9 - 35.1	36.4	0.036843	0.090429	4.048	8.888
35.1 - 31.0	32.8	0.041330	0.131759	5.037	13.925
31.0 - 28.7	29.7	0.016135	0.147894	2.170	16.095
28.7 - 21.9	24.3	0.036412	0.184306	5.994	22.088
21.9 - 16.0	18.0	0.021129	0.205435	4.707	26.796
16.0 - 13.4	14.5	0.005937	0.211372	1.641	28.437
13.4 - 11.3	12.2	0.003661	0.215034	1.202	29.640
11.3 - 10.5	10.9	0.000927	0.215960	0.340	29.980
10.5 - 8.2	9.1	0.001439	0.217399	0.633	30.612

Sample: resine Cagliari pre-macinazione
Operator:
Submitter:
File: C:\ASAP 2020 Plus\data\Chiappone\2022-1...\000-225.SMP

Started:	24/10/2022 09:45:14	Analysis adsorptive:	N2
Completed:	24/10/2022 18:12:00	Analysis bath temp.:	77,159 K
Report time:	24/10/2022 20:01:54	Thermal correction:	No
Sample mass:	0,4650 g	Ambient free space:	27,2106 cm³ Measured
Analysis free space:	81,8113 cm³	Equilibration interval:	10 s
Low pressure dose:	None	Sample density:	1,000 g/cm³
Automatic degas:	Yes		

Sample prep: Stage	Temperature (°C)	Ramp Rate (°C/min)	Time (min)
1	30	10	10

BJH Desorption Cumulative Pore Volume (Larger)

Harkins and Jura : Faas Correction

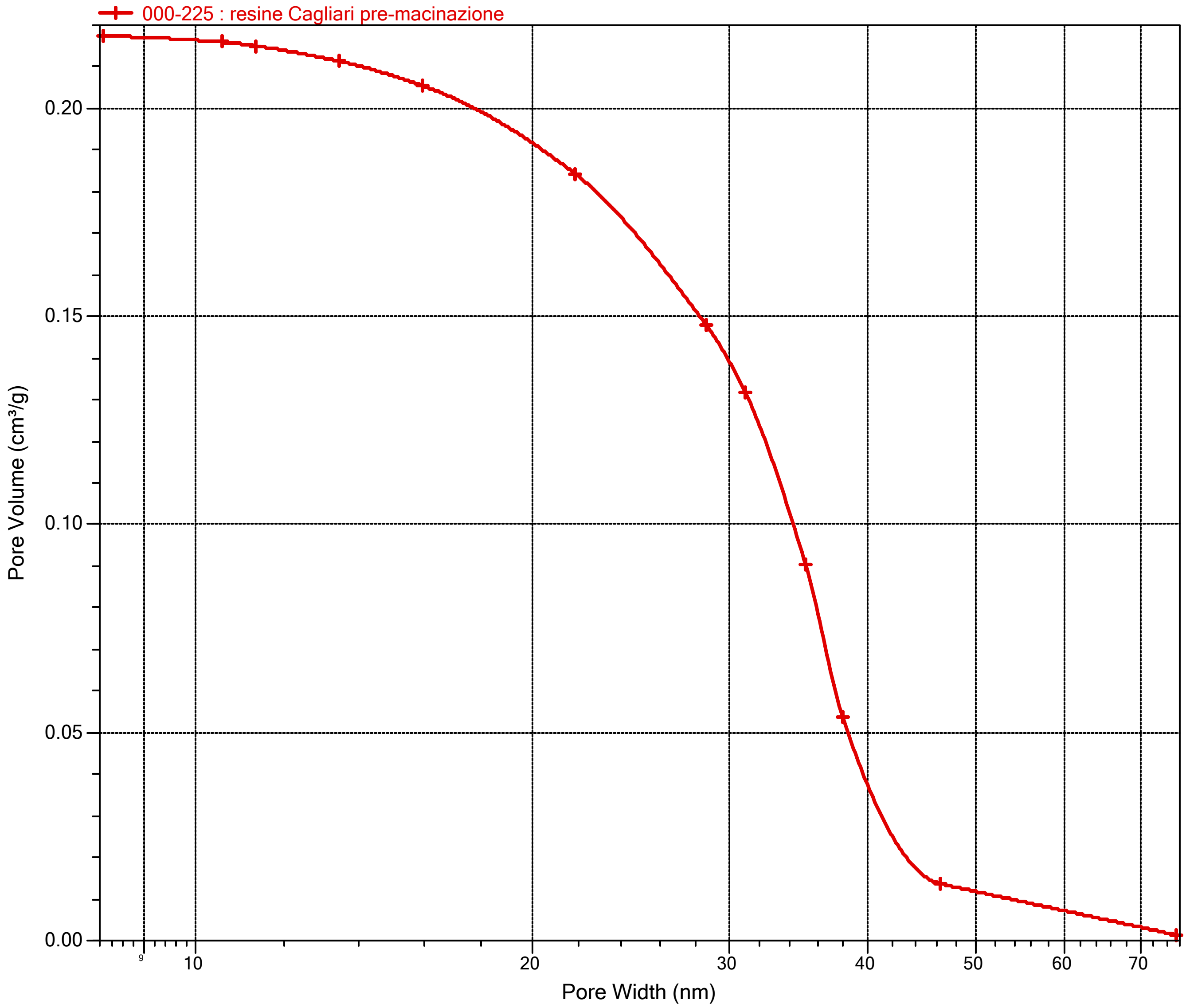

Sample: resine Cagliari pre-macinazione
Operator:
Submitter:
File: C:\ASAP 2020 Plus\data\Chiappone\2022-1...\000-225.SMP

Started:	24/10/2022 09:45:14	Analysis adsorptive:	N2
Completed:	24/10/2022 18:12:00	Analysis bath temp.:	77,159 K
Report time:	24/10/2022 20:01:54	Thermal correction:	No
Sample mass:	0,4650 g	Ambient free space:	27,2106 cm³ Measured
Analysis free space:	81,8113 cm³	Equilibration interval:	10 s
Low pressure dose:	None	Sample density:	1,000 g/cm³
Automatic degas:	Yes		

Sample prep: Stage	Temperature (°C)	Ramp Rate (°C/min)	Time (min)
1	30	10	10

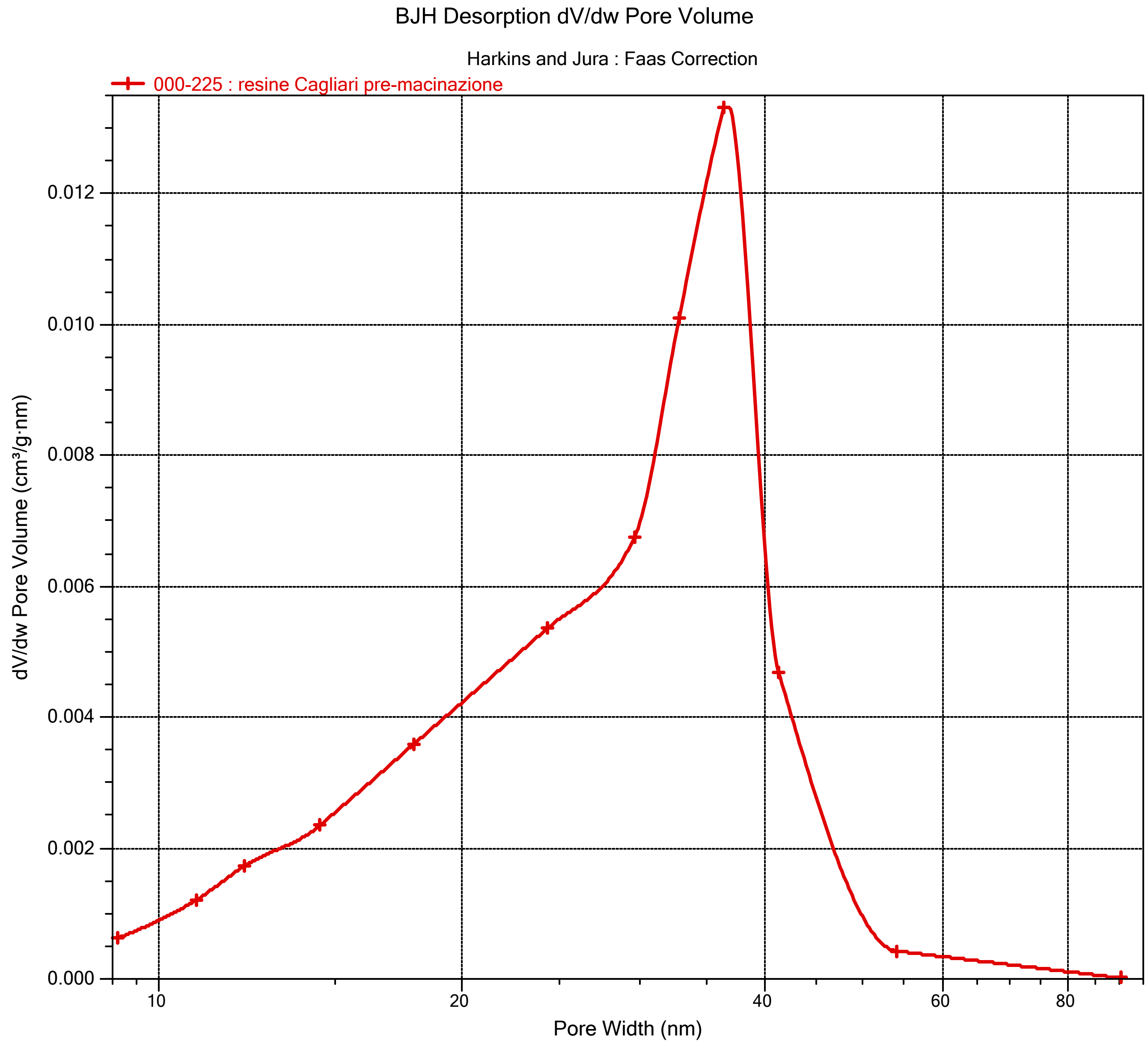

Sample: resine Cagliari pre-macinazione
Operator:
Submitter:
File: C:\ASAP 2020 Plus\data\Chiappone\2022-1...\000-225.SMP

Started:	24/10/2022 09:45:14	Analysis adsorptive:	N2
Completed:	24/10/2022 18:12:00	Analysis bath temp.:	77,159 K
Report time:	24/10/2022 20:01:54	Thermal correction:	No
Sample mass:	0,4650 g	Ambient free space:	27,2106 cm³ Measured
Analysis free space:	81,8113 cm³	Equilibration interval:	10 s
Low pressure dose:	None	Sample density:	1,000 g/cm³
Automatic degas:	Yes		

Sample prep: Stage	Temperature (°C)	Ramp Rate (°C/min)	Time (min)
1	30	10	10

DFT Pore Size Reports

Primary Data
4070- Unable to load deconvolution model Invalid.

Sample: resine Cagliari pre-macinazione
Operator:
Submitter:
File: C:\ASAP 2020 Plus\data\Chiappone\2022-1...\000-225.SMP

Started:	24/10/2022 09:45:14	Analysis adsorptive:	N2
Completed:	24/10/2022 18:12:00	Analysis bath temp.:	77,159 K
Report time:	24/10/2022 20:01:54	Thermal correction:	No
Sample mass:	0,4650 g	Ambient free space:	27,2106 cm³ Measured
Analysis free space:	81,8113 cm³	Equilibration interval:	10 s
Low pressure dose:	None	Sample density:	1,000 g/cm³
Automatic degas:	Yes		

Sample prep: Stage	Temperature (°C)	Ramp Rate (°C/min)	Time (min)
1	30	10	10

DFT Surface Energy Reports

Primary Data

4070- Unable to load deconvolution model Invalid.
